# Exosome-targeted delivery of METTL14 regulates NFATc1 m6A methylation levels to correct osteoclast-induced bone resorption

**DOI:** 10.1038/s41419-023-06263-4

**Published:** 2023-11-13

**Authors:** Jin-Gang Yang, Bao Sun, Zheng Wang, Xing Li, Jia-hui Gao, Jia-jun Qian, Jiang Li, Wen-jia Wei, Ping Zhang, Wei Wang

**Affiliations:** 1 Department of Stomatology, Tongren Hospital, Shanghai Jiao Tong University School of Medicine, No. 1111 Xianxia Road, Shanghai, 200336 China; 2 Department of Oral Pathology, Shanghai Ninth People’s Hospital, Shanghai Jiao Tong University School of Medicine; College of Stomatology, Shanghai Jiao Tong University; National Center for Stomatology; National Clinical Research Center for Oral Diseases; Shanghai Key Laboratory of Stomatology, No. 639 Zhizaoju Road, Shanghai, 200011 China; 3 Concordia Institute for Information Systems Engineering, Concordia University, 1455 De Maisonneuve Blvd. W., Montreal, QC H3G 1M8, Canada; 4 Department of Oral Maxillofacial-Head Neck Oncology, Shanghai Ninth People’s Hospital, Shanghai Jiao Tong University School of Medicine; College of Stomatology, Shanghai Jiao Tong University; National Center for Stomatology; National Clinical Research Center for Oral Diseases; Shanghai Key Laboratory of Stomatology, No. 639 Zhizaoju Road, Shanghai, 200011 China; 5 Department of Ecology, College of Life Sciences, Henan Normal University, No. 46 Jianshe East Road, Xinxiang, Henan Province 453007 China; 6 Department of Oral and Maxillofacial Surgery, Affiliated Hospital of Stomatology, Nanjing Medical University, No. 136 Hanzhong Road, Nanjing, Jiangsu Province 210029 China; 7 Department of General Dentistry, Shanghai Ninth People’s Hospital, Shanghai Jiao Tong University School of Medicine; College of Stomatology, Shanghai Jiao Tong University; National Center for Stomatology; National Clinical Research Center for Oral Diseases; Shanghai Key Laboratory of Stomatology, No. 639 Zhizaoju Road, Shanghai, 200011 China

**Keywords:** Methylation, Protein delivery, RNA modification

## Abstract

Osteoporosis has a profound influence on public health. First-line bisphosphonates often cause osteonecrosis of the jaw meanwhile inhibiting osteoclasts. Therefore, it is important to develop effective treatments. The results of this study showed that the increased level of NFATc1 m6A methylation caused by zoledronic acid (ZOL), with 4249A as the functional site, is highly correlated with the decreased bone resorption of osteoclasts. Upstream, METTL14 regulates osteoclast bone absorption through the methylation functional site of NFATc1. Downstream, YTHDF1 and YTHDF2 show antagonistic effects on the post-transcriptional regulation of NFATc1 after the m6A methylation level is elevated by METTL14. In this study, meRIP-Seq, luciferase reporter assays, meRIP and other methods were used to elucidate the NFATc1 regulatory mechanism of osteoclasts from the perspective of RNA methylation. In addition, EphA2 overexpression on exosomes is an effective biological method for targeted delivery of METTL14 into osteoclasts. Importantly, this study shows that METTL14 released by exosomes can increase the m6A methylation level of NFATc1 to inhibit osteoclasts, help postmenopausal osteoporosis patients preserve bone mass, and avoid triggering osteonecrosis of the jaw, thus becoming a new bioactive molecule for the treatment of osteoporosis.

## Introduction

Along with population ageing, there are more than 9 million new osteoporotic fractures worldwide every year. Postmenopausal osteoporosis patients have a lifetime fracture risk of approximately ~50% [1], while the mortality rate of hip fracture in women within one year is as high as 20–25% [2]. In China, osteoporosis patients accounted for 19.2% of the population over the age of 50 years, and women over the age of 50 years accounted for 32.1%, much higher than the prevalence rate of men. The prevalence rate of osteoporosis in women over the age of 65 years was as high as 51.6%. The cost of treating osteoporotic fractures is expected to reach a staggering $25.43 billion by 2050 in China [3]. Thus, the tremendous public health crisis and social-economic burden brought by osteoporosis [4, 5] is no less than that caused by cancer and cardiovascular and cerebrovascular diseases. Compared with the treatment of osteoporotic fractures, the cost of prevention and treatment of early osteoporosis is relatively low. Correction of the initial symptoms has become the most effective and cost-effective treatment for osteoporosis.

Bisphosphonates are powerful bone resorptive inhibitors [6] and are the first choice for the treatment of osteoporosis, multiple myeloma, bone metastasis and other diseases [7]. These drugs promote bone resorption by inhibiting the mevalonate pathway and promoting apoptosis of osteoclasts [8]. Bisphosphonates are landmark drugs in the treatment of osteoporosis and tumour related bone diseases. However, in decades of clinical application, bisphosphonates have caused serious side effects. Many clinical cases of bisphosphonate-related osteonecrosis of the jaw (BRONJ) have been reported and have drawn widespread attention [9]. Here, we describe the pathogenesis of BRONJ as follows based on previous studies. The binding of bisphosphonates to the jaw not only directly damages bone cells and causes a local inflammatory environment but also prevents osteoblast-induced new bone formation due to the low rate of bone remodelling [10, 11]. In addition, bisphosphonates inhibit osteoclast differentiation, leading to the failure of osteoclasts to degrade necrotic bone tissue, promoting the expansion of lesions and the invasion of odontogenic and periodontal bacteria, and aggravating the local inflammatory environment. However, bisphosphonates inhibit angiogenesis, delay the migration of neutrophils, macrophages and osteoclast precursor cells into the lesion and delay granulation tissue crawling and fibroblast repair [12–15]. Pathogenic bacteria in bone tissue continue to aggravate lesion infection, and the lack of osteoclasts leads to the difficult excretion of sequestrum and pus, resulting in severe osteonecrosis [16–19].

m6A methylation is the most common form of RNA methylation, and as an epigenetic modification, it participates in and regulates many important functions of RNA [20, 21]. m6A methylation is a dynamic and reversible process. The METTL3/METTL14 methyltransferase complex and the WTAP cofactor are primarily involved in the methylation of m6A, and the process of demethylation is primarily completed by the methylases FTO and ALKBH5 [22–26]. In mammals, m6A reader proteins mainly include members of the YTH protein family (YTHDF1, 2, 3 and YTHDC1, 2) and members of the heterogeneous nuclear ribonucleoprotein family (HNRNPA2B1, HNRNPC and HNRNPG) [27–31]. Many human diseases are closely related to m6A modification, such as immune function regulation, fat metabolism, viral replication and infection, neurodevelopment and disorders [32]. m6A methylation is common in all eukaryotic cells. It can be inferred that m6A methylation is involved in every stage of the human biological process [26, 33, 34], and disorder regulation may also lead to the occurrence and development of most diseases [35–37]. All bone metabolic diseases and diseases related to tumour bone metastasis are bidirectionally regulated by osteoblasts and osteoclasts [38, 39]. However, there are few studies of RNA methylation in the osteoclast field compared with the research direction of osteoblast. In 2021, we confirmed that circ_0008542 affected the expression of RANK in osteoclasts through m6A methylation modification, thus changing the function of bone resorption [40]. This result indicated that m6A methylation plays an important role in the pathogenesis of bone metabolic diseases, and molecular-targeted therapies aimed at m6A methylation will supplement or even replace the treatment of traditional drugs in the future.

Based on these conclusions, we speculate that RNA methylation may participate in the process by which bisphosphonates inhibit osteoclasts. Therefore, in this study, ZOL was added in the process of osteoclast differentiation, and m6A immunoprecipitation (meRIP) sequencing and RNA sequencing were performed. The intersecting target genes between the two groups were selected. Kyoto Encyclopedia of Genes and Genomes (KEGG) was used to analyse gene function, metabolic pathways and biological processes. The nuclear factor of activated T cells type C1 (NFATc1) gene, which is highly correlated with osteoclast differentiation, was screened from the results for further study. In this study, we proved the relationship between m6A methylation level of NFATc1 gene and osteoclast-induced bone resorption while identifying m6A methylation functional sites of the gene. It could be regulated osteoclast differentiation to correct abnormal bone resorption in osteoporosis through the functional sites of the NFATc1 gene.

## Results

### High-throughput sequencing and differentially expressed genes

**Fig. 1:**
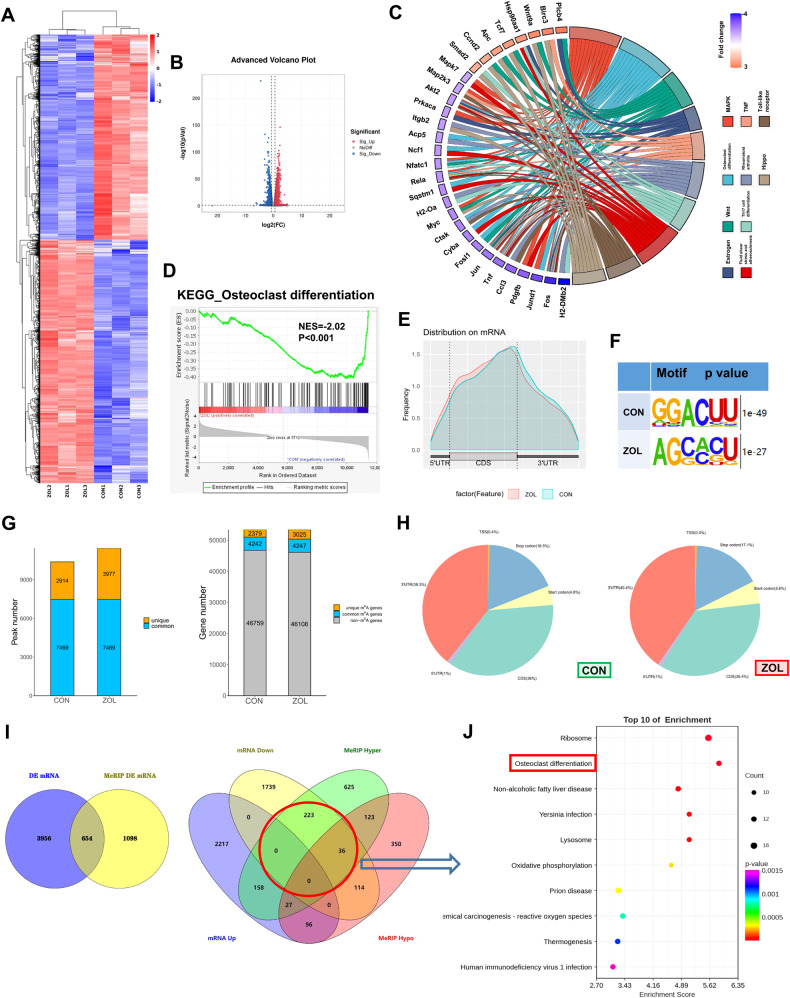
High-throughput sequencing and differentially expressed genes. RAW264.7 cells were separately collected with or without ZOL (5 μM) stimulation after RANKL induction. Total RNA extracted from the abovementioned two groups was subjected to RNA-seq and m6A-seq. **A**, **B** Heatmap and volcano plot showing differentially expressed mRNAs between the CON and ZOL groups. **C** The KEGG terms were visualised in a chord plot. **D** Gene set enrichment analysis (GSEA) plot evaluating the alterations in the osteoclast differentiation process using RNA sequence data. **E** The profiles of m6A enrichment across the mRNA transcriptome in both groups. **F** Predominant consensus motifs identified m6A-seq peaks in both groups. **G** Number of m6A peaks and m6A-modified genes identified in m6A-seq between the CON and ZOL groups. **H** Graphs of the m6A peak distribution showing the proportion of total m6A peaks in both groups. **I** Venn diagrams showing 654 intersecting genes with differential expression and differential m6A-methylation in both groups (left). The differentially expressed genes were classified according to the level of mRNA and m6A methylation (right). **J** A bubble chart showing the enrichment of the biological process of interest using the differentially expressed genes obtained by intersecting genes (in the red circle of Fig. 1I).

**Fig. 2:**
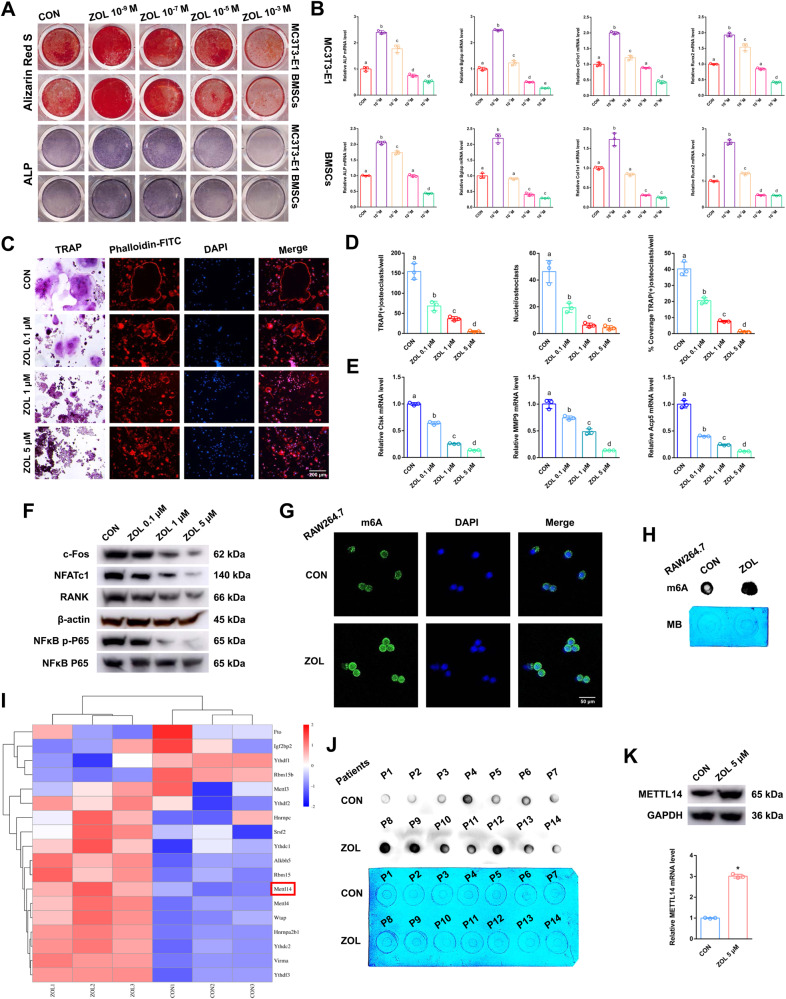
The effects of ZOL on both osteoblasts and osteoclasts. **A** After addition of different concentrations of ZOL, Alizarin Red S or ALP staining was applied to MC3T3-E1 cells and BMSCs after 21 days or 7 days of osteogenic induction. **B** Relative expression levels of ALP, Bglap, Col1α1 and Runx2 in MC3T3-E1 cells and BMSCs at different concentrations of ZOL. **C** TRAP staining and F-actin band staining were applied to detect osteoclast differentiation and bone resorption ability at different concentrations of ZOL. Scale bar: 200 μm. **D** Histograms of the number, coverage rate and nuclei of TRAP-positive osteoclasts at different concentrations of ZOL. **E** Relative expression levels of Ctsk, MMP9 and Acp5 in RAW264.7 cells at different concentrations of ZOL. **F** Protein levels of c-fos, NFATc1, RANK and NFκB p-P65 in RAW264.7 cell lysates at different concentrations of ZOL were analysed by western blots. **G**, **H** Immunofluorescence images and m6A dot blot assay showing the global m6A levels of RAW264.7 cells after ZOL stimulation. Scale bar: 50 μm. **I** Heatmap showing the differentially expressed m6A methylation related enzymes after ZOL stimulation. **J** m6A dot blot assay showing the global m6A levels of postmenopausal women with or without bisphosphonate treatment. **K** After ZOL stimulation, the mRNA and protein expression levels of METTL14 in RAW264.7 cells were detected by RT-qPCR and western blotting, respectively. Data are representative of three independent experiments expressed as the mean ± SD (*p < 0.05). Different letters (a, b, c, d and e) indicate significant differences among multiple groups (*p* < 0.05).

RAW264.7 cells were separately collected with or without ZOL (5 μM; Fig. 2C–F) stimulation after RANKL induction. Total RNA extracted from the abovementioned two groups was subjected to RNA-seq and m6A-seq. KEGG analysis maps molecular datasets from genomics, transcriptomics, proteomics and metabolomics onto the following signalling pathway maps to explain the corresponding molecular biological functions. A heatmap and volcano plot showed differentially expressed mRNA between the CON and ZOL groups (Fig. 1A, B). KEGG analysis showed that the differentially expressed genes were associated with osteoclast differentiation after ZOL stimulation (Fig. 1C). KEGG enrichment analysis of genes with changes in expression after ZOL stimulation indicated the osteoclast differentiation process (Fig. 1D). These m6A modifications were predominantly located in protein-coding transcripts and enriched near the stop codons in both the CON and ZOL groups (Fig. 1E). We mapped the m6A motif with independent biological replicates. The GGACU motif was identified to be highly enriched within the m6A site, consistent with the m6A consensus sequence RRACH (R = G or A; H = A, C or U; Fig. 1F). In general, m6A-seq of the CON and ZOL groups identified 2914 and 3977 unique m6A peaks, respectively, and 2379 and 3025 unique m6A-modified genes, respectively (Fig. 1G). We next investigated the m6A distribution patterns. Similar patterns of m6A distribution were observed in both the CON and ZOL groups (Fig. 1H). RNA-seq and m6A-seq were combined to analyse the intersecting genes. The threshold of differentially expressed genes from RNA-seq and m6A-seq was set to be greater than 1.5 times, and the *P* value was <0.05. The corresponding intersecting genes were selected for further analysis (Fig. 1I) according to the osteoclast phenomena in which the m6A methylation level increased (Fig. 2G, H) and the transcriptional level decreased (Fig. 2E, F) after ZOL stimulation. A bubble chart showing the enrichment of the biological process of interest using the differentially expressed genes obtained by intersecting genes (Fig. 1I) was created (Fig. 1J). The top ten biological functions between the two groups are presented, and osteoclast differentiation function is marked in the red box (Fig. 1J).

### ZOL effects on both osteoblasts and osteoclasts

Alizarin red S (ARS) and Alkaline phosphatase (ALP) staining were applied to detect osteoblast bone formation after different stimulation with concentrations of ZOL. Low concentration stimulation (ZOL 10^–9^ M) promoted osteoblast bone formation, but high concentration stimulation (ZOL more than 10^–5^ M) had the opposite effect in both MC3T3-E1 cells and bone marrow stromal cells (BMSCs) (Fig. 2A). In addition, the expression levels of the bone formation marker genes ALP, Bglap, Col1α1 and Runx2 in MC3T3-E1 cells and BMSCs showed the same tendency based on the RT-qPCR results (Fig. 2B). We evaluated the direct effects of stimulation with different concentrations of ZOL on RANKL-induced osteoclast differentiation. From the results of tartrate-resistant acid phosphatase (TRAP) and F-actin band staining, a decreasing number of osteoclasts with shrunken TRAP-positive cell bodies and F-actin bands and fewer nuclei were notably identified as the concentration increased (Fig. 2C). From the results of the histogram coverage rate, the number and nuclei of TRAP-positive osteoclasts were significantly decreased as the concentration increased (Fig. 2D). RT-qPCR analysis revealed that the expression of Ctsk, MMP9 and Acp5 mRNA induced by RANKL decreased as the concentration increased (Fig. 2E). Western blotting revealed that the expression levels of c-fos, NFATc1, RANK and NFκB p-P65 induced by RANKL were decreased as the concentration increased (Fig. 2F). These results implied that ZOL inhibits RANKL-induced osteoclastic differentiation as the concentration increases, and the inhibitory effect was most obvious when ZOL reached 5 μM.

### ZOL or METTL14 inhibits osteoclast differentiation, and increases the m6A methylation level on osteoclast precursor cells

**Fig. 3:**
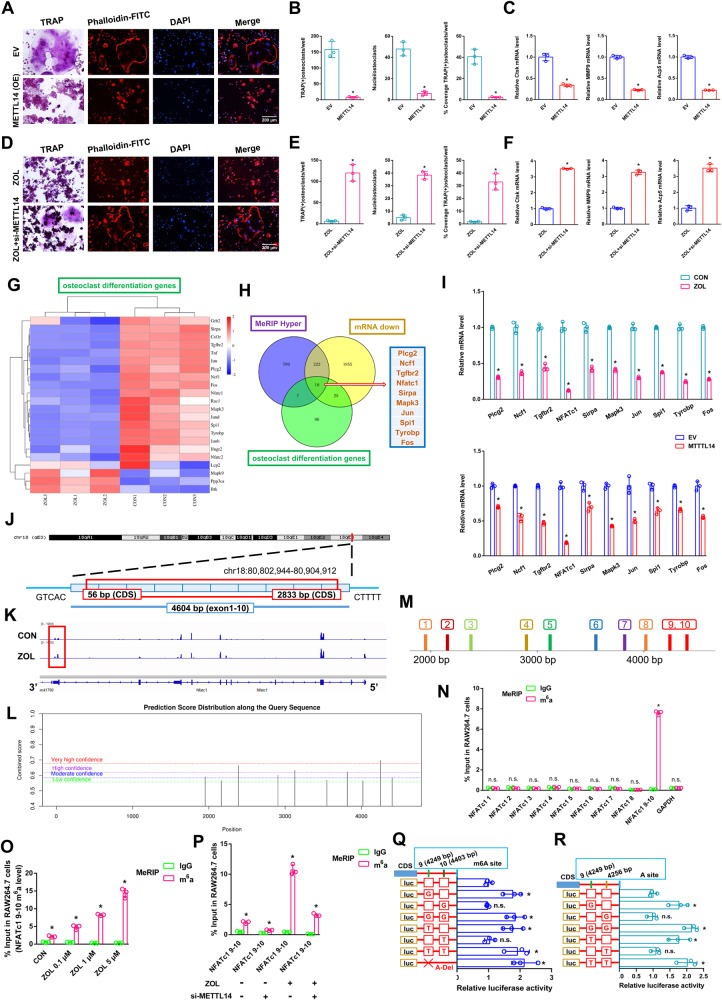
The effects of the NFATc1–9 segment on the process by which METT14 regulates osteoclast differentiation. First, METTL14 was transfected into RAW264.7 cells and prepared for further study. **A** TRAP staining and F-actin band staining were applied to detect osteoclast differentiation between the two groups. Scale bar: 200 μm. **B** Histograms of the number, coverage rate and nuclei of TRAP-positive osteoclasts between the two groups. **C** Relative expression levels of Ctsk, MMP9 and Acp5 in RAW264.7 cells between the two groups. After ZOL stimulation, si-METTL14 was transfected into RAW264.7 cells and prepared for further study. **D** TRAP staining and F-actin band staining were applied to detect osteoclast differentiation between the two groups. Scale bar: 200 μm. **E** Histograms of the number, coverage rate and nuclei of TRAP-positive osteoclasts between the two groups. **F** Relative expression levels of Ctsk, MMP9 and Acp5 in RAW264.7 cells between the two groups. **G** Heatmap showing the differentially expressed genes of osteoclast differentiation after ZOL stimulation. **H** KEGG analysis revealing the differentially expressed genes from three intersections: osteoclast differentiation genes, MeRIP hyper genes and mRNA down genes. **I** RT-qPCR analysis revealing the expression levels of 10 differentially expressed genes after ZOL stimulation or METTL14 overexpression. **J** Schematic diagram showing the genomic location of NFATc1. **K** m6A peak visualisation of meRIP-Seq in NFATc1 transcripts with or without ZOL stimulation. The m6A peaks are in the 3’UTRs of NFATc1. **L** The potential m6A methylation loci of NFATc1 on the SRAMP website. **M** We divided 9 segments of NFATc1 according to the potential m6A methylation loci. **N** m6A-RT-qPCR assay was performed to detect the enrichment of 9 segments of NFATc1 between the anti-m6A group and anti-IgG group after ZOL stimulation. **O** An m6A-RT-qPCR assay was performed to detect the enrichment of the NFATc1–9, 10 segment as the ZOL concentration increased. **P** An m6A-RT-qPCR assay was performed to detect the enrichment of the NFATc1–9, 10 segment under ZOL or si-METTL14 stimulation. **Q**, **R** Two m6A modification sites were validated through step-by-step mutation of luciferase reporters. Data are representative of three independent experiments expressed as the mean ± SD (*p < 0.05).

m6A modification is an important post-transcriptional regulatory mechanism of intracellular mRNA expression. As detected by immunofluorescence assays and dot blot assays, the global m6A methylation level was strongly increased after ZOL stimulation (Fig. 2G, H). The expression of m6A “writers”, “erasers”, and “readers” was evaluated in both the CON and ZOL groups. Among these candidates, METTL14 was markedly increased in the ZOL-stimulated cells, which could be responsible for the increase in m6A methylation levels (Fig. 2I). A dot blot assay using an m6A antibody was performed and revealed that the m6A level was lower in the alveolar bone from postmenopausal women (P1-P7) without bisphosphonate treatment but higher in the alveolar bone from postmenopausal women (P8-P14) with bisphosphonate treatment for at least 1 year (Fig. 2J). RT-qPCR analysis revealed that the expression of METTL14 mRNA increased in the alveolar bone from postmenopausal women (P8-P14) with bisphosphonate treatment (Fig. S1A). This finding indicates that an increase in the m6A methylation level may play an important role in ZOL mediated inhibition of RANKL-induced osteoclastic differentiation. We next added ZOL or transfected METTL14 or si-METTL14 into RAW264.7 cells for further study. METTL14 was substantially increased after ZOL stimulation (Fig. 2K). Moreover, western blotting and RT-qPCR assays verified the successful construction of cell lines (Fig. S1B, C). As shown in the results of the abovementioned experiments (TRAP staining, F-actin band staining and RT-qPCR), upregulation of METTL14 inhibited osteoclast differentiation after we transfected METTL14 into RAW264.7 cells (Fig. 3A–C). In addition, downregulation of METTL14 rescued osteoclast differentiation after ZOL stimulation when we transfected si-METTL14 into RAW264.7 cells (Fig. 3D–F). These results proved that METTL14 plays a pivotal role during RANKL-induced osteoclast differentiation. It plays a similar role as ZOL.

### The NFATc1 gene is involved in the process by which METT14 inhibits osteoclast differentiation, and only the NFATc1–9 segment reveals a high level of m6A methylation

**Fig. 4:**
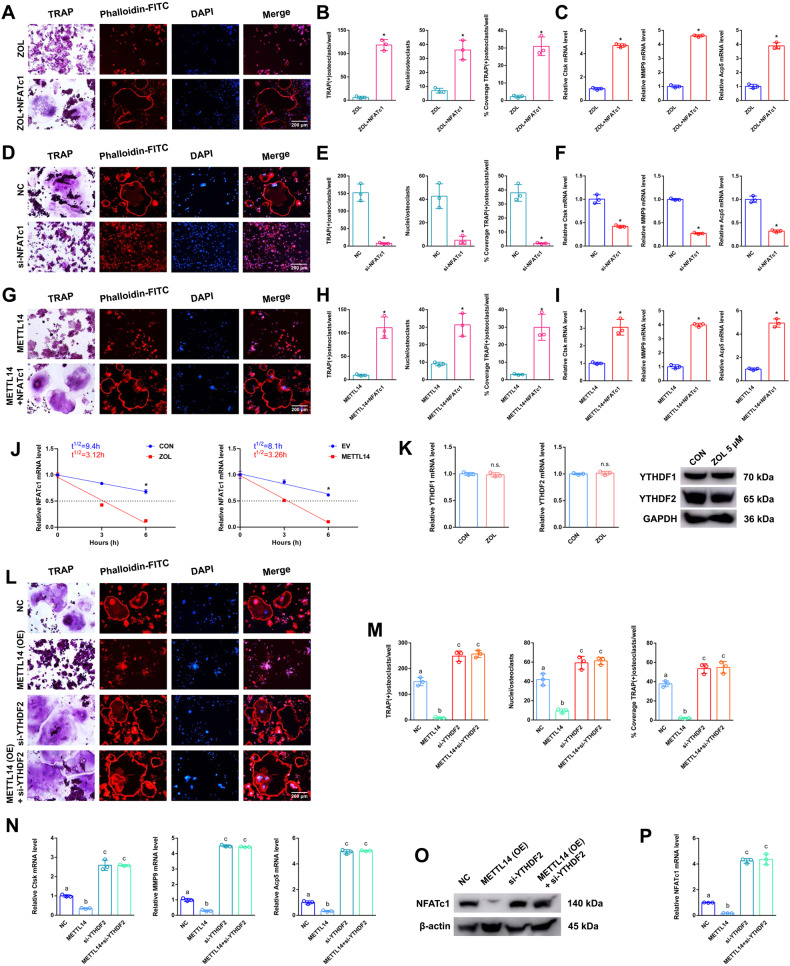
The effects of METTL14 on the post-transcriptional regulation of NFATc1. After ZOL stimulation, NFATc1 was transfected into RAW264.7 cells and prepared for further study. **A** TRAP staining and F-actin band staining were applied to detect osteoclast differentiation between the two groups. Scale bar: 200 μm. **B** Histograms of the number, coverage rate and nuclei of TRAP-positive osteoclasts between the two groups. **C** Relative expression levels of Ctsk, MMP9 and Acp5 in RAW264.7 cells between the two groups. Next, si-NFATc1 was transfected into RAW264.7 cells and prepared for further study. **D** TRAP staining and F-actin band staining were applied to detect osteoclast differentiation between the two groups. Scale bar: 200 μm. **E** Histograms of the number, coverage rate and nuclei of TRAP-positive osteoclasts between the two groups. **F** Relative expression levels of Ctsk, MMP9 and Acp5 in RAW264.7 cells between the two groups. Next, NFATc1 or METTL14 was transfected into RAW264.7 cells separately or simultaneously according to the different groups and prepared for further study. **G** TRAP staining and F-actin band staining were applied to detect osteoclast differentiation between the two groups. Scale bar: 200 μm. **H** Histograms of the number, coverage rate and nuclei of TRAP-positive osteoclasts between the two groups. **I** Relative expression levels of Ctsk, MMP9 and Acp5 in RAW264.7 cells between the two groups. **J** Under ZOL or METTL14 stimulation, the NFATc1 mRNA half-life was estimated according to linear regression analysis. **K** After ZOL stimulation, the mRNA and protein expression levels of YTHDF1 and YTHDF2 in RAW264.7 cells were detected by RT-qPCR and western blotting, respectively. Next, si-YTHDF2 or METTL14 was transfected into RAW264.7 cells separately or simultaneously according to the different groups and prepared for further study. **L** TRAP staining and F-actin band staining were applied to detect osteoclast differentiation among the four groups. Scale bar: 200 μm. **M** Histograms of the number, coverage rate and nuclei of TRAP-positive osteoclasts among the four groups. **N** Relative expression levels of Ctsk, MMP9 and Acp5 in RAW264.7 cells among the four groups. **O**, **P** The mRNA and protein expression levels of NFATc1 in RAW264.7 cells among the four groups were analysed by RT-qPCR and western blotting, respectively. Data are representative of three independent experiments expressed as the mean ± SD (*p < 0.05). Different letters (a, b, c, d and e) indicate significant differences among multiple groups (*p* < 0.05).

A heatmap showed differentially expressed genes related to osteoclast differentiation between the CON and ZOL groups (Fig. 3G). KEGG analysis revealed 10 differentially expressed genes from three intersections: osteoclast differentiation genes, MeRIP hyper genes and mRNA down genes (Fig. 3H). RT-qPCR analysis revealed the expression levels of 10 differentially expressed genes after ZOL stimulation or METTL14 overexpression. NFATc1 expression significantly decreased compared to that of other genes under the above conditions (Fig. 3I). RT-qPCR analysis revealing the expression levels of 10 differentially expressed genes between ZOL group and ZOL+si-METTL14 group. After ZOL+si-M14 stimulation, 10 decreased genes were significantly restored compared with ZOL stimulation (Fig. S1D). NFATc1 is located on chromosome 18: 80, 802, 994-80, 904, 912 (4604 nt), and the genomic structure suggests that NFATc1 consists of 10 exons (Fig. 3J and Table S1). Visualisation analysis indicated that the NFATc1 m6A methylation level in the ZOL group was notably enriched in the CDS-3’ untranslated regions (3’UTRs) of mRNA compared to the CON group (Fig. 3K). We applied the SRAMP website to predict the abundance of m6A methylation loci of NFATc1. Ten potential loci were observed in NFATc1’s overall length (Fig. 3L and S2). To determine the effective m6A methylation segments in NFATc1, we designed 9 pairs of primers that amplified 9 sections of the NFATc1 sequence (Fig. 3M). From the results of m6A-RT-qPCR, only the NFATc1–9, 10 segment revealed a high level of m6A methylation after ZOL stimulation (Fig. 3N). The m6A methylation level of the NFATc1–9, 10 segment increased as the ZOL concentration increased (Fig. 3O). This finding is consistent with the NFATc1 m6A sequencing results (Fig. 3K). To determine the effect of ZOL or si-METTL14 on NFATc1–9, 10 m6A methylation, we added ZOL or transfected si-METTL14 into RAW264.7 cells. After ZOL stimulation, the previously high level of the m6A methylated NFATc1–9, 10 segment was decreased when METTL14 was simultaneously disturbed in RAW264.7 cells based on m6A-RT-qPCR results, indicating that either ZOL or METTL14 regulates the m6A methylation level through the segment of NFATc1–9, 10 (Fig. 3P). Nest, two m6A modification sites were validated via step-by-step mutation of the reporting plasmids. Specifically, different NFATc1 mutation fragments (4249A-G, 4249A-T, 4249A-Delete, 4403A-G, 4403A-T, 4256A-G, 4256A-T) were separately inserted into the luciferase reporter. The results showed that NFATc1–9 (4249 bp A) is the effective m6A methylation site (Fig. 3Q, R). This site determines the m6A methylation level of NFATc1 and is regulated by ZOL or METTL14. We next transfected NFATc1 or si-NFATc1 into RAW264.7 cells for further study. Western blotting and RT-qPCR assays verified the successful construction of cell lines (Fig. S3A, B). As shown in the results of the abovementioned experiments (TRAP staining, F-actin band staining and RT-qPCR), upregulation of NFATc1 rescued osteoclast differentiation after ZOL stimulation when we transfected NFATc1 into RAW264.7 cells (Fig. 4A–C). In addition, downregulation of NFATc1 inhibited osteoclast differentiation after we transfected si-NFATc1 into RAW264.7 cells (Fig. 4D–F). In addition, upregulation of NFATc1 rescued osteoclast differentiation after METTL14 stimulation when we cotransfected METTL14 and NFATc1 into RAW264.7 cells (Fig. 4G–I). RT-qPCR analysis revealed that the expression of NFATc1 mRNA decreased in the alveolar bone from postmenopausal women (P8-P14) with bisphosphonate treatment (Fig. S3C). To analyse the effect of m6A levels on NFATc1 mRNA metabolism, we conducted RNA stability assays. The assays showed that ZOL stimulation or METTL14 overexpression shortened the half-life of NFATc1 mRNA transcripts in RAW264.7 cells (Fig. 4J). These results proved that NFATc1 plays a pivotal role during ZOL/METTL14-mediated inhibition of osteoclast differentiation.

### YTHDF2 shows negative effects on post-transcriptional regulation of NFATc1 after elevated methylation levels by METTL14

**Fig. 5:**
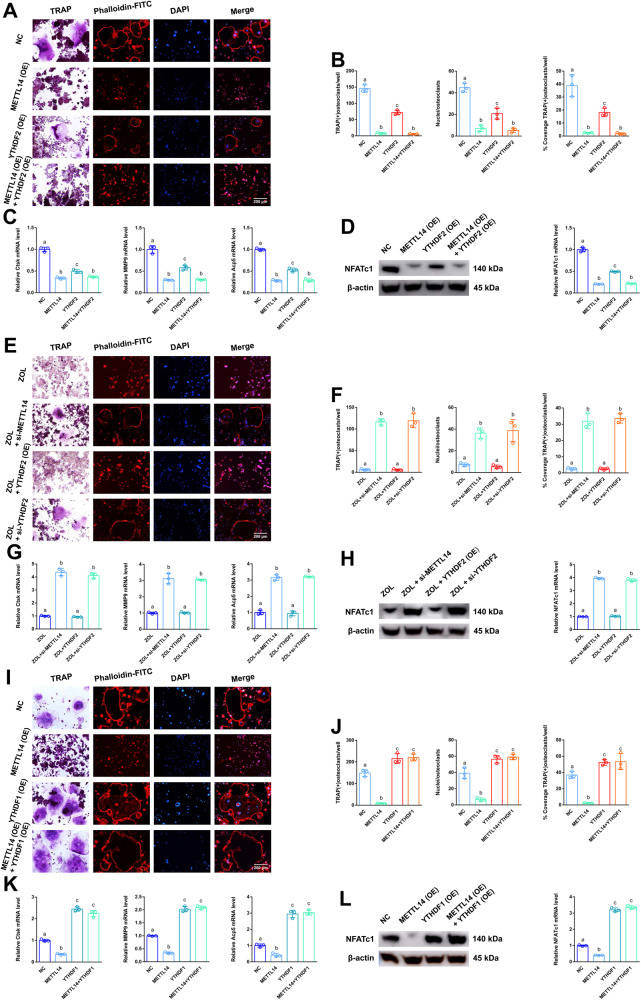
The effects of YTHDF1 and YTHDF2 on the post-transcriptional regulation of NFATc1. YTHDF2 or METTL14 was transfected into RAW264.7 cells separately or simultaneously according to the different groups and prepared for further study. **A** TRAP staining and F-actin band staining were applied to detect osteoclast differentiation among the four groups. Scale bar: 200 μm. **B** Histograms of the number, coverage rate and nuclei of TRAP-positive osteoclasts among the four groups. **C** Relative expression levels of Ctsk, MMP9 and Acp5 in RAW264.7 cells among the four groups. **D** The mRNA and protein expression levels of NFATc1 in RAW264.7 cells among the four groups were analysed by RT-qPCR and western blotting, respectively. After ZOL stimulation, YTHDF2, si-YTHDF2 or si-METTL14 was transfected into RAW264.7 cells separately according to the different groups and prepared for further study. **E** TRAP staining and F-actin band staining were applied to detect osteoclast differentiation among the four groups. Scale bar: 200 μm. **F** Histograms of the number, coverage rate and nuclei of TRAP-positive osteoclasts among the four groups. **G** Relative expression levels of Ctsk, MMP9 and Acp5 in RAW264.7 cells among the four groups. **H** The mRNA and protein expression levels of NFATc1 in RAW264.7 cells among the four groups were analysed by RT-qPCR and western blotting, respectively. Next, YTHDF1 or METTL14 was transfected into RAW264.7 cells separately or simultaneously according to the different groups and prepared for further study. **I** TRAP staining and F-actin band staining were applied to detect osteoclast differentiation among the four groups. Scale bar: 200 μm. **J** Histograms of the number, coverage rate and nuclei of TRAP-positive osteoclasts among the four groups. **K** Relative expression levels of Ctsk, MMP9 and Acp5 in RAW264.7 cells among the four groups. **L** The mRNA and protein expression levels of NFATc1 in RAW264.7 cells among the four groups were analysed by RT-qPCR and western blotting, respectively. Data are representative of three independent experiments expressed as the mean ± SD. Different letters (a and b) indicate significant differences among multiple groups (*p* < 0.05).

To determine the effect of “readers” on NFATc1 mRNA expression, we next transfected YTHDF2 or si-YTHDF2 into RAW264.7 cells for further study. After ZOL stimulation, YTHDF1 and YTHDF2 levels were not changed (Fig. 4K). Western blotting and RT-qPCR assays verified the successful construction of cell lines (Fig. S4A, B). RT-qPCR analysis revealed that the expression of YTHDF2 mRNA were not changed in the alveolar bone from postmenopausal women (P8-P14) with bisphosphonate treatment (Fig. S4C). For “readers” YTHDC2 was markedly increased in the ZOL-stimulated cells (Fig. 2I). It was found that YTHDC2 did not play a role in osteoclast differentiation, so we set out to verify YTHDF2 (Fig. S5). As shown in the results of the abovementioned experiments (TRAP staining, F-actin band staining and RT-qPCR), only downregulation of YTHDF2 enhanced osteoclast differentiation compared to that of the NC group. In addition, downregulation of YTHDF2 rescued osteoclast differentiation after METTL14 stimulation when we cotransfected METTL14 and si-YTHDF2 into RAW264.7 cells (Fig. 4L–N). Western blotting and RT-qPCR assays verified the same trend of NFATc1 at the mRNA and protein levels in the corresponding groups (Fig. 4O, P). Next, as shown in the results of the abovementioned experiments (TRAP staining, F-actin band staining and RT-qPCR), only upregulation of YTHDF2 slightly decreased osteoclast differentiation compared to that of the NC group. In addition, upregulation of YTHDF2 markedly decreased osteoclast differentiation after METTL14 stimulation when we cotransfected METTL14 and YTHDF2 into RAW264.7 cells (Fig. 5A–C). Western blotting and RT-qPCR assays verified the same trend of NFATc1 at the mRNA and protein levels in the corresponding groups (Fig. 5D). As shown in the results of the abovementioned experiments (TRAP staining, F-actin band staining and RT-qPCR), upregulation of YTHDF2 markedly decreased osteoclast differentiation after ZOL stimulation. In addition, downregulation of YTHDF2 increased osteoclast differentiation after ZOL stimulation when we transfected si-YTHDF2 into RAW264.7 cells (Fig. 5E–G). Western blotting and RT-qPCR assays verified the same trend of NFATc1 at the mRNA and protein levels in the corresponding groups (Fig. 5H).

### YTHDF1 and YTHDF2 show antagonistic effects on post-transcriptional regulation of NFATc1 after elevated methylation levels by METTL14

**Fig. 6:**
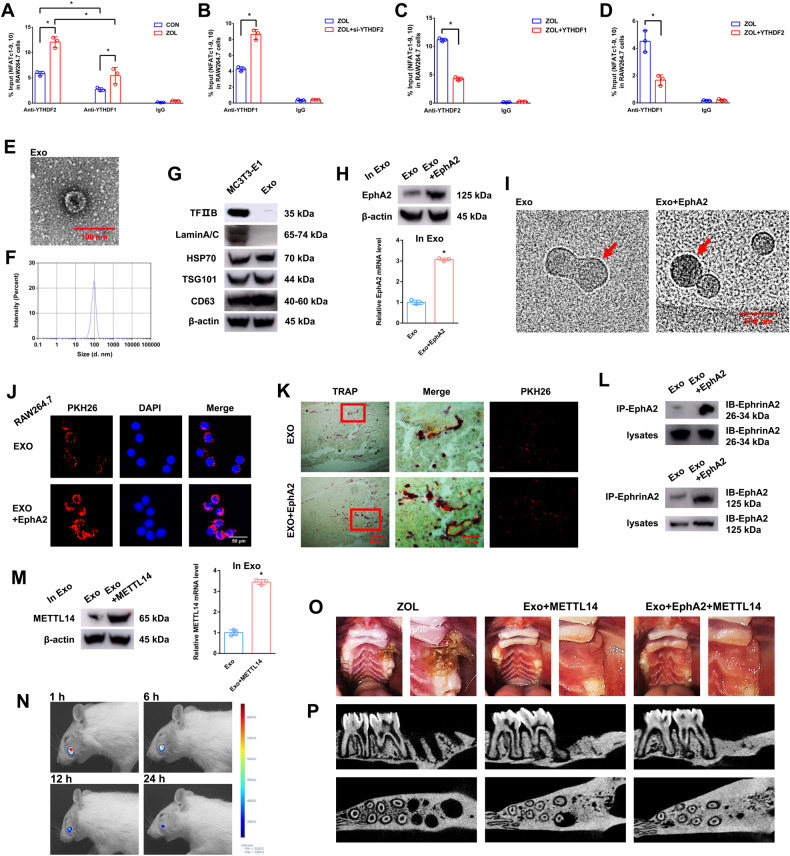
The effects of EphA2-EphrinA2 on osteoclasts receiving exosomes. **A**–**D** RIP assays were performed to detect the enrichment rate of the NFATc1–9, 10 segment under different conditions. **E** Electron microscopy images of exosomes. Scale bar: 100 nm. **F** Size distribution of exosomes secreted by MC3T3-1 cells. **G** Protein levels of TFIIB, Lamin A/C, HSP70, TSG101 and CD63 in MC3T3-E1 cell lysates or exosomes secreted by MC3T3-E1 cells analysed by western blotting. **H** After transfection of EphA2 into ME3T3-E1 cells, the mRNA and protein expression levels of EphA2 in exosomes were detected by RT-qPCR and western blotting, respectively. **I** Cryo-transmission electron microscopy images of exosomes. The red arrow indicates that the nanogold probe (1.4 nm) combined with the EphA2 receptor. Scale bar: 100 nm. **J** Immunofluorescence images of exosomes from MC3T3-E1 cells into RAW264.7 cells. Exosomes were labelled with PKH26 and overexpressed with or without EphA2. Scale bar: 50 nm. **K** Fluorescence microscopy and TRAP staining analysis revealing PKH26-labelled exosomes injected into bone marrow. Red boxes indicate that exosomes were absorbed by osteoclasts. Scale bars: 200 μm, 50 μm. **L** Coimmunoprecipitation (co-IP) analysis indicating the binding effects of EphA2-EphrinA2 and EphrinA2-EphA2. **M** After transfection of METTL14 into ME3T3-E1 cells, the mRNA and protein expression levels of METTL14 in exosomes were detected by RT-qPCR and western blotting, respectively. **N** Rat in vivo imaging analysis showed that exosome labelling with Cy5.5 was specifically located in the socket after the left maxillary first molar was removed. **O** After 8 weeks of tooth extraction, the representative pictures of rat bisphosphonate-related osteonecrosis of the jaw (BRONJ) and two exosome treatment groups (Exo+METTL14 and Exo+EphA2 + METTL14). **P** After 8 weeks of ZOL or exosome injection, micro-CT was applied to present the bone histological changes among the groups. Data are representative of three independent experiments expressed as the mean ± SD (*p < 0.05).

To detect the effect of the “reader” (YTHDF1) on NFATc1 mRNA expression, we next transfected YTHDF1 into RAW264.7 cells for further study. Western blotting and RT-qPCR assays verified the successful construction of cell lines (Fig. S4D). As shown in the results of the abovementioned experiments (TRAP staining, F-actin band staining and RT-qPCR), upregulation of YTHDF1 increased osteoclast differentiation compared to that of the NC group. In addition, upregulation of YTHDF1 markedly increased osteoclast differentiation after METTL14 stimulation when we cotransfected METTL14 and YTHDF1 into RAW264.7 cells (Fig. 5I–K). Western blotting and RT-qPCR assays verified the same trend of NFATc1 at the mRNA and protein levels in the corresponding groups (Fig. 5L). RNA immunoprecipitation (RIP) assays revealed that the NFATc1–9, 10 segment was enriched in the anti-YTHDF2 and anti-YTHDF1 groups after ZOL stimulation. In addition, this value was higher in the anti-YTHDF2 group than in the anti-YTHDF1 group under both CON and ZOL conditions (Fig. 6A). This finding proves that the binding efficiency of YTHDF2 to the NFATc1–9, 10 segment is higher than that of YTHDF1 in both CON and ZOL conditions. Next, when we applied si-YTHDF2 or YTHDF1 or YTHDF2 overexpression, the binding efficiency of YTHDF2 with the NFATc1–9, 10 segment showed an opposite trend compared with that of YTHDF1 (Fig. 6B–D). These results demonstrated that YTHDF2 played a major role in the process of NFATc1 mRNA degradation compared to YTHDF1 after ZOL stimulation, even though the expression levels of YTHDF1 and YTHDF2 did not change after the increase in NFATc1 m6A methylation levels. Furthermore, upstream, ZOL or METTL14 increases the m6A methylation level of the NFATc1 gene through its methylation functional site. Downstream, YTHDF1 and YTHDF2 show competitive effects on the post-transcriptional regulation of NFATc1 after the m6A methylation level is elevated. Specifically, YTHDF2 downregulates NFATc1 through m6A-dependent mRNA decay. In contrast, YTHDF1 upregulates NFATc1 in an m6A-dependent manner.

### Osteoclasts receive more exosomes from osteoblasts through the EphA2-EphrinA2 pathway

Representative exosomes were observed under transmission electron microscopy (Fig. 6E). The size distribution of exosomes indicated that the diameter of particles was mostly concentrated at 100 nm (Fig. 6F). Western blotting assays verified that cytomembrane characteristic proteins (HSP70, TSG101 and CD63) were present in both MC3T3-E1 cell lysates and exosomes, and cell nucleus characteristic proteins (TFIIB and Lamin A/C) were present in MC3T3-E1 cell lysates but absent in exosomes (Fig. 6G). We next transfected EphA2 into MC3T3-E1 cells and then extracted exosomes for further study. Western blotting and RT-qPCR assays verified that EphA2 was overexpressed in exosomes (Fig. 6H). Then, we applied a nanogold antibody to prove that EphA2 was overexpressed on the surface of exosomes through cryo-transmission electron microscopy with immunohistochemistry (Fig. 6I). Next, the immunofluorescence assay demonstrated that exosomes overexpressing EphA2 could be highly efficiently absorbed by osteoclast precursor cells compared to that of the EXO group (Fig. 6J). In an in vivo study, different exosomes labelled with PKH26 were injected into bone marrow. Fluorescence microscopy and TRAP staining analysis revealed that exosomes overexpressing EphA2 could be highly efficiently absorbed by osteoclasts compared to that of the EXO group (Fig. 6K). Furthermore, coimmunoprecipitation (co-IP) analysis indicated that the binding between EphA2 and EphrinA2 was significantly increased in the Exo+EphA2 group compared to the Exo group. Moreover, co-IP analysis indicated that the binding between EphrinA2 and EphA2 showed the same trend (Fig. 6L). These data suggested that EphA2 and EphrinA2 are responsible for exosome-targeted delivery from osteoblasts to osteoclasts.

### METTL14 in exosomes does not induce osteonecrosis of the jaw in vivo

To detect the effect of METTL14 in exosomes, we next transfected METTL14 into MC3T3-E1 cells and then extracted exosomes for further study. Western blotting and RT-qPCR assays verified the successful METTL14 overexpression in exosomes (Fig. 6M). In an in vivo study, exosomes labelled with Cy5.5 were injected into the socket after the left maxillary first molar was removed. Fluorescence activity was visualised at different time points (Fig. 6N). The establishment of the rat BRONJ model was confirmed by the fact that the socket was not covered with mucosa, and the alveolar bone was exposed after 8 weeks of tooth extraction. However, osteonecrosis of the jaw did not occur in either the Exo+METTL14 group or the Exo+EphA2 + METTL14 group compared to the ZOL group (Fig. 6O). In a comparison of each sample by micro-CT, there was less hard tissue in the extraction socket of the ZOL group than in the other two groups (Fig. 6P).

### METTL14 in exosomes displays a clear rescue effect on bone loss in vivo

**Fig. 7:**
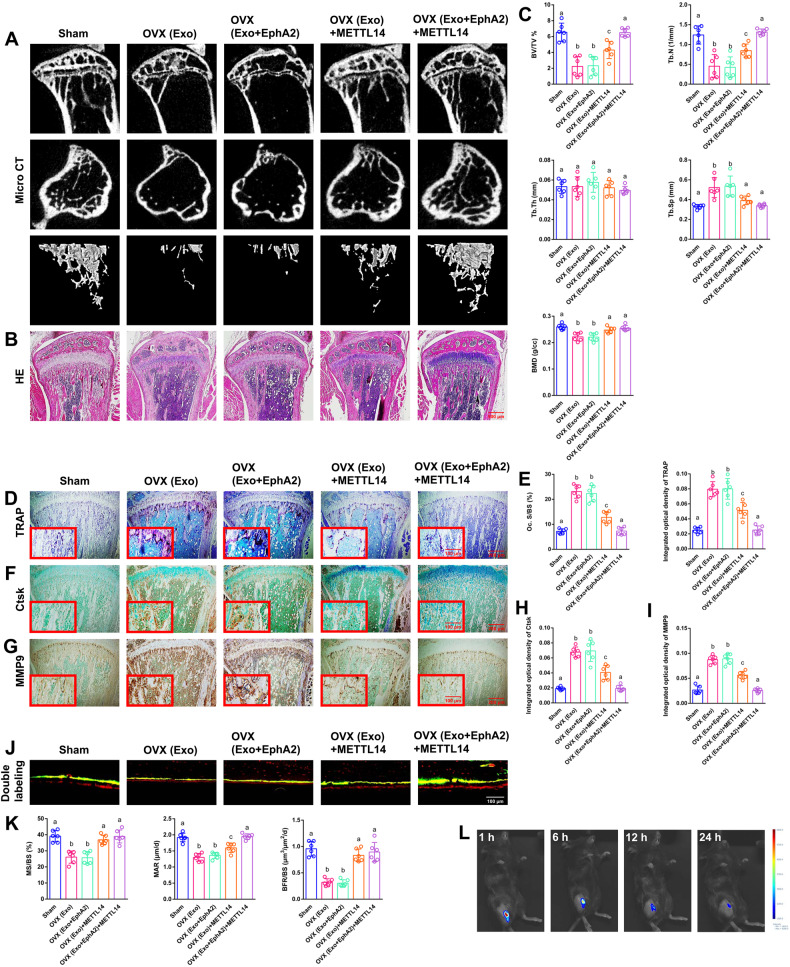
The effects of METTL14 in vivo. EphA2 or METTL14 was transfected into MC3T3-E1 cells and collected from cell supernatant exosomes separately or simultaneously according to the different groups. **A**, **B** After 8 weeks of exosome injection, micro-CT and H&E staining were applied to measure the bone histological parameters. Scale bar: 500 nm. **C** Histograms of BV/TV, Tb.N, Tb.Th, Tb.Sp and BMD among the five groups. **D** TRAP staining was applied to evaluate osteoclast differentiation and bone resorption ability. Scale bars: 200 μm, 100 μm. **E** Histograms of osteoclast Oc. S/BS and integrated optical density of TRAP among the five groups. **F**, **G** Immunohistochemistry of Ctsk and MMP9 staining was applied to evaluate osteoclast differentiation and bone resorption ability. Scale bars: 200 μm, 100 μm. **H**, **I** Histograms of integrated optical density of Ctsk and MMP9 among the five groups. **J**, **K** Representative images of dynamic histomorphometry of cortical bone with quantification of MS/BS, MAR and BFR/BS. Scale bar: 100 μm. **L** Mouse in vivo imaging analysis showed that exosomes labelled with Cy5.5 were specifically located in the tibia bone marrow. Data are representative of six independent experiments expressed as the mean ± SD. Different letters (a, b and c) indicate significant differences among multiple groups (*p* < 0.05).

**Fig. 8:**
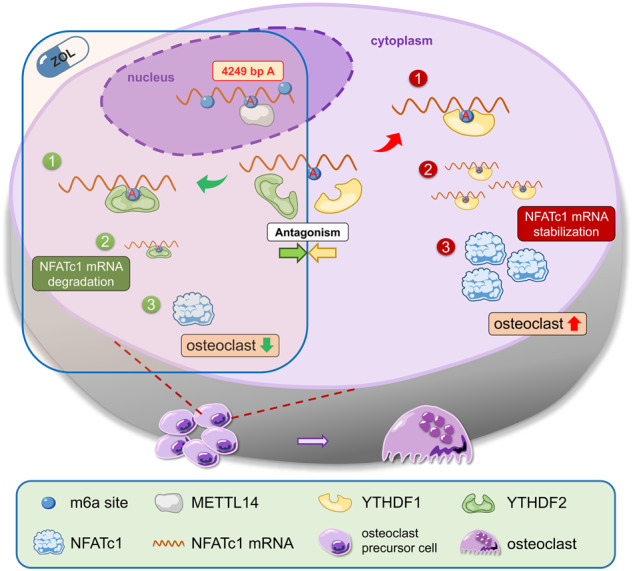
Schematic diagram of this study. The increased level of NFATc1 m6A methylation caused by ZOL, in which 4249 A is the functional site, is highly correlated with the decreased function of bone resorption of osteoclasts. Upstream, METTL14 regulates osteoclast bone absorption through the methylation functional site of NFATc1. Downstream, YTHDF1 and YTHDF2 show competitive effects on the post-transcriptional regulation of NFATc1 after the m6A methylation level is elevated by METTL14.

EphA2 or METTL14 was transfected into MC3T3-E1 cells and collected from cell supernatant exosomes separately or simultaneously according to the different groups. Micro-CT and haematoxylin and eosin (H&E) staining were applied to measure bone histological parameters. After 8 weeks of exosome injection, the sham-ovariectomized (OVX) group and the OVX+EphA2 group displayed clear bone loss by histology compared to the other groups, including decreased trabecular bone number, thinner metaphyseal trabeculae and increased trabecular spacing. The OVX+EphA2 + METTL14 group displayed a clearer rescue effect on bone loss than the OVX + METTL14 group (Fig. 7A, B). Compared to the other groups, the OVX group and the OVX+EphA2 group exhibited observably decreased bone volume/total volume (BV/TV), bone mineral density (BMD) and trabecular number (Tb.N) and significantly increased trabecular separation (Tb.Sp). Compared to the OVX + METTL14 group, the OVX+EphA2 + METTL14 group exhibited observably increased BV/TV and Tb.N (Fig. 7C). As expected from TRAP staining, the OVX group and the OVX+EphA2 group showed a considerably increased number of TRAP-positive osteoclasts compared to the other groups. However, the OVX + METTL14 group and the OVX+EphA2 + METTL14 group showed a notable reversal of this phenomenon. The OVX+EphA2 + METTL14 group was the most similar to the sham group (Fig. 7D). Several significant differences among the five groups with regard to the osteoclast surface/bone surface (Oc. S/BS) and the integrated optical density of TRAP are presented in the histograms (Fig. 7E). Immunohistochemistry assays of Ctsk and MMP9 verified the same trend in the corresponding groups (Fig. 7F, G). Several significant differences among the five groups with regard to the integrated optical density of Ctsk and MMP9 are presented in the histograms (Fig. 7H, I). Dynamic histomorphometry of double fluorescence labelling showed that both OVX groups had remarkably lower cortical bone. The OVX + METTL14 group and the OVX+EphA2 + METTL14 group showed a notable rescue of this phenomenon. The OVX+EphA2 + METTL14 group was the most similar to the sham group (Fig. 7J). Mineralising surface/bone surface (MS/BS), mineral apposition rate (MAR) and bone formation rate/bone surface (BFR/BS) verified the same trend in the corresponding groups (Fig. 7K). Next, exosomes labelled with Cy5.5 were injected into the bone marrow. Fluorescence activity was visualised at different time points (Fig. 7L). A schematic diagram of this study is presented in Fig. 8.

## Discussion

NFATc1 is the most effective transcription factor during the later period of RANKL-induced osteoclast differentiation [41]. Normally, NFATc1 has a low affinity for DNA and is found in stationary cytoplasm in a highly phosphorylated state. However, when the concentration of Ca^2+^ increases after cell activation, calcineurin activity is enhanced, and NFATc1 can remain activated for a long time in the nucleus. When Calcineurin activity is inhibited, NFATc1 rapidly reverts to its phosphorylated state, thus returning to the cytoplasm and reducing its affinity for DNA [42, 43]. In the process of osteoclast differentiation, RANKL binds to RANK to activate the downstream NFκB signalling pathway, resulting in increased intracellular Ca^2+^ concentration, followed by increased calcineurin activity that dephosphorylates NFATc1 into the nucleus [44, 45]. NFATc1 induces the expression of osteoclast-specific genes in the nucleus, including DC-STAMP, which promotes the fusion of osteoclast precursor cells, and genes related to bone resorption, such as TRAP, CK and β3 integrin [46–48].

ZOL, a third-generation and nitrogen-containing bisphosphonate, is currently recognised as the first-line pharmacologic treatment for osteoporosis. It selectively suppresses osteoclastic bone resorption by effectively inhibiting farnesyl pyrophosphate synthase activity in the mevalonate pathway. It has strong anti-resorptive effects, and simultaneously inhibits osteoclast differentiation and triggers osteoclast apoptosis [49–53]. Compared with previous research, this study elucidates the mechanism of osteoclast differentiation from the perspective of RNA methylation. Specifically, the NFATc1 gene is involved in the process by which ZOL inhibits osteoclasts in osteoporosis. RNA m6A methylation plays a key role as an important epigenetic modification in the post-transcriptional regulation of NFATc1, which affects osteoclast differentiation through its methylation functional site (4249 bp A). After ZOL stimulation, the highly expressed METTL14 enhances the NFATc1 m6A methylation level through the 4249 A site. Downstream, YTHDF1 and YTHDF2 show antagonistic effects on post-transcriptional regulation of NFATc1, with the elevated m6A methylation level acting as a precursor condition. YTHDF2 induced the degradation of NFATc1 mRNA by binding to the m6A modification sites mediated by METTL14. Conversely, at the NFATc1 mRNA translation level, recognition and binding of m6A modification sites by YTHDF1 result in enhanced protein synthesis. Under normal conditions, when ZOL is added, YTHDF2 plays a leading role in reducing NFATc1 mRNA. If YTHDF2 is decreased or YTHDF1 is overexpressed at the same time, YTHDF1 will play a dominant role in promoting NFATc1 protein synthesis. This finding explained the phenomenon that NFATc1 decreased after ZOL stimulation or METTL14 overexpression. In addition, YTHDF1 and YTHDF2 did not change after ZOL stimulation or METTL14 overexpression. Thus, the basal expression of YTHDF2 in cells still plays a dominant role. Next, exosomes were used as carriers for osteoporosis correction in vivo, which can transport METTL14 into osteoclasts. High levels of METTL14 play the same role as ZOL, increasing the methylation level of NFATc1 and inhibiting osteoclast differentiation.

This study found a strong correlation between m6A methylation level of NFATc1 gene and osteoclast-induced bone resorption. Moreover, 4249 A is the m6A methylation functional site of the NFATc1 gene. A similar conclusion has been reported in previous studies. m6A alters the local structure in mRNA and lncRNA to facilitate binding of heterogeneous nuclear ribonucleoprotein C (hnRNP C). The 2,577 m6A residue specifically destabilises the lncRNA MALAT1 hairpin-stem to increase the accessibility or single-strandedness of the opposite U-tract and improve the interaction with hnRNP C. The mechanism that regulates RNA-protein interactions through m6A-dependent RNA structural remodelling is termed an “m6A switch” [54]. Moreover, another study confirmed that m6A methylation regulates the molecular sponge effect between lincRNA1281 and the let-7 miRNA family, thus affecting the normal differentiation of mouse embryonic stem cells. This RNA-RNA interaction is m6A methylation dependent, yet the functional site mutation (917 bp A to G, 1025 bp A to G, 1056 bp A to G) at lincRNA1281 leads to loss of the molecular sponge effect [55]. Furthermore, we found a similar phenomenon in our previous study. METTL3 acts on the m6A functional site of 1956 bp in circ_0008542, promoting competitive binding of miRNA-185–5p by circ_0008542, leading to an increase in the target gene RANK and initiating osteoclast bone absorption. Circ_0008542 is generated from osteoblasts after tension stimulation, and 1956 A is the “m6A switch” of circ_0008542 [40]. Based on the previous conclusions, we named 4249 A the “m6A switch” in this study. The m6A functional site is the core of relevant biological effects. This “m6A switch” at NFATc1 4249 A is closely related to osteoclast differentiation and bone resorption. METTL14, YTHDF1 or YTHDF2 appears to be a node that controls the “m6A switch” to varying degrees. Both results of our studies suggest that m6A methylation-related bone resorption of osteoclasts is collectively regulated by different key molecules (RANK upstream and NFATc1 downstream) in the same signalling pathway (NFκB). This finding proves that m6A methylation plays a pivotal role in the process of osteoclast differentiation, and each key molecule has m6A methylation functional sites on its gene. This theoretical underpinning offers a fresh idea to treat osteoporosis by precisely controlling osteoclast differentiation via m6A methylation functional sites.

In an in vivo study, exosome-released METTL14 enhanced NFATc1’s m6A methylation level to inhibit osteoclasts. This phenomenon is a novel molecular mechanism in the process of osteoclast differentiation after ZOL stimulation. In addition, compared with bisphosphonates, METTL14 released by exosomes did not trigger osteonecrosis of the jaw in vivo. Therefore, METTL14 will be a safer biologic treatment for osteoporosis than traditional medicine ZOL. Furthermore, we have discovered a novel approach to boost exosome-targeted delivery’s effectiveness. Osteoclasts can take up more METTL14 from exosomes both in vitro and in vivo through EphA2 receptor overexpression on the surface of exosomes from osteoblasts. This phenomenon is because of the corresponding EphrinA2 ligand on the surface of osteoclasts. This finding suggests that osteoblasts and osteoclasts can transmit biological signals to each other through the EphA2-EphrinA2 pathway. Combined with the clinical problem that needs to be solved in this study, we think that osteoporosis has a profound influence on public health, and inhibition of osteoclast-induced bone resorption is the core solution. We believe that EphA2 overexpression on exosomes is an effective biological vector for targeted inhibition of osteoclasts to help postmenopausal osteoporosis patients preserve bone mass while avoiding the side effects of bisphosphonates. Importantly, this study provides a method to prevent osteoclast differentiation through the use of exosomes releasing METTL14.

## Materials and methods

### Cell line, antibodies, human subjects and ethics statement

Murine RAW264.7 monocytic and MC3T3-E1 cell lines were purchased from the Shanghai Cell Center (Shanghai, China). Recombinant soluble mouse RANKL was purchased from R&D Systems (Minneapolis, USA). Specific antibodies against NFκB p65 (#8242; 1:1000), phospho-NFκB p65 (#3033; 1:1000), NFATc1 (#8032; 1:1000), c-fos (#2250; 1:1000), METTL14 (#51104; 1:1000), YTHDF1 (#57530; 1:1000), YTHDF2 (#71283; 1:1000), EphA2 (#6997; 1:1000) and β-actin (#4970; 1:1000) were obtained from Cell Signaling Technology. Specific antibodies against HSP70 (ab181606; 1:1000), TSG101 (ab133586; 1:1000), CD63 (ab217345; 1:1000), TFIIB (ab109106; 1:1000), RANK (ab200369; 1:1000), m6A (ab284130; 1:5000), Ctsk (ab19027; 1:100), MMP9 (ab38898; 1:100) and GAPDH (ab181602; 1:10000) and goat anti-rabbit (Alexa Fluor) (ab150077; 1:1000) and horseradish peroxidase-conjugated goat anti-rabbit antibodies (ab205718; 1:10000) were obtained from Abcam. Lamin A/C (NBP2–19324; 1:1000) was obtained from Novus. EphrinA2 (PA5–85778; 1:1000) was obtained from Thermo Fisher.

The alveolar bone in females was harvested from volunteer donors who underwent dental implantation due to molar loss for at least 1 year in the affiliated hospital of stomatology of Nanjing Medical University. During dental implantation surgery, we used a ring drill to obtain alveolar bone from postmenopausal (65–75 years old) women with at least 1 year of bisphosphonate treatment and postmenopausal (65–75 years old) women without bisphosphonate treatment. All clinical specimens in this study were approved by the Ethics Committee of the School of Stomatology of Nanjing Medical University. Written informed consent was received from all patients. All animal experiments in this study were conducted according to the Guidelines for Animal Experimentation of the School of Stomatology of Nanjing Medical University. The Laboratory Animal Care and Use Committees of the hospital approved all experimental procedures.

### ALP staining and ARS staining

ALP staining was performed using a BCIP/NBT staining kit (Beyotime, China). After osteogenic induction for 7 days, cells were fixed, and ALP staining was performed following the manufacturer’s instructions. Mineralised nodule formation was determined by ARS staining. After osteogenic incubation for 21 days, cells were fixed and stained with 0.1% ARS (Sigma-Aldrich, USA) for 20 min.

### Osteoclast differentiation, TRAP staining and F-actin band staining

RAW264.7 cells were seeded in 24-well plates and cultured in DMEM containing 10% FBS and 1% penicillin/streptomycin. Cells were subjected to treatments according to experimental requirements. Cells were then stimulated with RANKL (20 ng/ml) for 7 days. The culture medium was replaced with fresh medium every other day. After 7 days, TRAP staining was used to evaluate osteoclast differentiation. Cells were fixed and subjected to TRAP staining, which proceeded as described in our previous study [40]. Multinucleated TRAP-positive cells with at least 3 nuclei were scored as osteoclasts. RAW264.7 cells were seeded in 24-well plates and cultured for 7 days as previously described. Cells were fixed and stained for F-actin band staining. Briefly, cells were submerged in 5 μl/ml phalloidin (Yeasen, China) for 30 min after treatment with 0.5% Triton X-100 for 5 min.

### Western blot analysis

RIPA lysis buffer was used to extract total protein from cultured cells. Equal quantities of proteins were separated using SurePAGE gel (Genscript, USA) electrophoresis and transferred onto polyvinylidene difluoride (PVDF) membranes (Millipore, USA). PVDF membranes were incubated with primary antibodies overnight at 4 °C. After washing, PVDF membranes were incubated with secondary antibodies. Protein bands were visualised using ECL chemiluminescence reagent (Millipore, USA).

### RT-qPCR analysis

Total RNA was extracted using TRIzol reagent (Takara, Japan) and was reverse transcribed using PrimeScript^TM^ RT Master Mix RR036A (Takara, Japan). Primer sequences were designed and synthetised by Sangon Biotech (Table S2). RT-qPCR was conducted with SYBR Premix Ex Taq^TM^ RR420A (Takara, Japan). Relative expression levels of the target genes were calculated by the 2^-∆∆Ct^ method. GAPDH was used for normalisation, and the data were compared to normalised control values. In addition, specific siRNA sequences are listed in Table S3.

### M6A immunoprecipitation

The MeRIP assay was conducted with the Magna MeRIP™ m6A Kit (Millipore, USA) to determine m6A modification of individual transcripts. In brief, total RNA was isolated from pretreated cells and randomly fragmented into a size of 100 nucleotides. RNA samples were then immunoprecipitated with magnetic beads precoated with anti-m6A antibody or anti-IgG. N6-methyladenosine 5’-monophosphate sodium salt was applied to elute the m6A-modified RNA fragments for further RT-qPCR analysis. Specific primers were designed for RT-qPCR analysis according to the SRAMP website (m6A loci predictor http://www.cuilab.cn/sramp/) and are listed in Table S4. The relative enrichment of m6A was normalised to the input.

### Dot blot and m6A quantification

Preheated RNA was spotted on a positively charged nylon membrane (Beyotime, China). The m6A status was probed with m6A antibody and detected using ECL detection reagents (Millipore, USA). The same membrane was also stained with methylene blue as a control. For immunofluorescence analysis, the fixed cells were treated with m6A antibody and fluorescent-conjugated secondary antibody. The results were then visualised with a confocal laser scanning microscope (Leica Microsystems, Wetzlar, Germany).

### Luciferase reporter assay

Cells were transfected with pmirGLO-based luciferase vector fused or not fused to wild-type or mutated NFATc1 (3’UTRs). METTL14 or empty vectors were cotransfected. Renilla and firefly luciferase activities were measured separately using the Dual Luciferase Reporter Assay System (Promega, USA) following the manufacturer’s instructions. Renilla luciferase was used to normalise firefly luciferase activity to evaluate reporter translation efficiency. Experiments were performed in triplicate.

### RNA immunoprecipitation

The RIP assay was performed with a Magna RIP Kit (Millipore, USA) according to the manufacturer’s protocol. Briefly, magnetic beads were mixed with anti-YTHDF1, anti-YTHDF2 or anti-IgG before the addition of cell lysates. After the protein beads were removed, RNAs of interest were eluted from the immunoprecipitated complex and purified for further analysis using RT-qPCR. Relative enrichment was normalised to the input.

### Exosome isolation, transmission electron microscopy and particle size analysis

Cell supernatants were centrifuged at 2000 × *g* for 15 min at 4 °C to exclude cell debris. Supernatants were filtered through 0.22 μm filters (Millipore, USA) and then centrifuged at 110,000 × g for 30 min in Amicon Ultra −3 kDa (Millipore, USA). Pellets were resuspended in the appropriate amount of PBS and stored at −80 °C. Freshly prepared exosomes were resuspended in 100 μl of 2% paraformaldehyde, absorbed on Formvar-carbon coated EM grids, washed with PBS, fixed with 1% glutaraldehyde and washed with water. Thereafter, grids were stained with 4% uranyl-oxalate solution, embedded in 1% methyl cellulose-UA, and observed under electron microscopy at 80 kV. A Mastersizer 2000 instrument (Malvern, UK) was used to analyse the distribution of exosome size. Freshly prepared exosome samples were resuspended in PBS and measured according to the manufacturer’s instructions.

### Cryo-transmission electron microscopy with immunohistochemistry

Exosomes were acquired as previously described. Pellets were resuspended and incubated in the primary antibody (EphA2). Then, the pellets were centrifuged in an Amicon Ultra −3 kDa (Millipore, USA). Freshly prepared exosomes were absorbed on EM grids. Then they were incubated in the secondary antibody (1.4 nm nanogold-antibody, Nanoprobes, USA). Thereafter, the grids were observed under a Tecnai G2 F20 electron microscope at 200 kV.

### Animals and exosome injection

Eight-week-old female C57BL/6 mice were used in this study. All mice were randomly distributed into 5 groups: sham, where mice underwent OVX and were injected with exosomes containing mock vehicle by periosteal injection into the marrow cavity of the tibiae for 8 weeks (exosomes 30 μg/week, for 8 weeks, *n* = 6); OVX, where mice underwent standard OVX and were injected with exosomes containing mock vehicle (same dose, *n* = 6); OVX+EphA2, where mice underwent standard OVX and were injected with exosomes containing EphA2 (same dose, *n* = 6); OVX + METTL14, where mice underwent standard OVX and were injected with exosomes containing METTL14 (same dose, *n* = 6); and OVX+EphA2 + METTL14, where mice underwent standard OVX and were injected with exosomes containing EphA2 and METTL14 (same dose, *n* = 6).

Twelve-week-old female Wistar rats were used in this study. All rats were randomly distributed into 3 groups: ZOL, where rats underwent standard OVX, had the left maxillary first molar removed and were injected with ZOL by tail vein injection for 8 weeks (additional four weeks in advance, 35 μg/kg/2 week, *n* = 4); Exo+METTL14, where rats underwent standard OVX, had the left maxillary first molar removed and were injected with exosomes containing METTL14 by socket injection for 8 weeks (exosomes 100 μg/week, for 8 weeks, *n* = 4); and Exo+EphA2 + METTL14, where rats underwent standard OVX, had the left maxillary first molar removed and were injected with exosomes containing EphA2 + METTL14 by socket injection for 8 weeks (same dose, *n* = 4). EphA2 or METTL14 was transfected into MC3T3-E1 cells, and then, different exosomes were extracted and injected according to the corresponding experimental groups.

### Micro-CT, histological detection, immunohistochemistry, TRAP staining and exosome distribution assay

Maxillary bone and tibiae were scanned using Skyscan 1176 Micro-CT (Bruker, USA) with a scanning resolution of 9 μm, a voltage of 50 kV and a current of 450 μA. Trabecular bone data were obtained at a region of interest along the long axis of the proximal tibiae and 1–3 mm away from the growth plate. The bone histomorphometry parameter analysis included BV/TV, Tb.N, trabecular thickness (Tb.Th), Tb.Sp and BMD. Serial sections were made for subsequent histological analysis. H&E staining was applied to assess histological alterations. Prepared sections were incubated with primary antibodies against Ctsk or MMP9. They were then washed and incubated with secondary antibodies. Satisfactory immunostaining was acquired in the presence of diaminobenzidine (DAB) (Sigma-Aldrich, USA). TRAP staining was performed as previously described. For evaluation of the distribution of exosomes in maxillary bone and tibiae, Cy5.5-labelled exosomes were applied in an in vivo study. Images were captured after 1, 6, 12 and 24 h using an in vivo imaging system.

### Fluorescence labelling analysis

For observation of the mineralising front, the mice were given a subcutaneous injection with 20 mg/kg calcein (Sigma, USA) and 30 mg/kg alizarin red S (Sigma, USA) at 8 and 2 days, respectively, before euthanasia. The undecalcified bone samples were dehydrated and embedded in pure resin blocks. The blocks were cut along the center of the tibial axis to obtain thin sections. Pictures were captured to study new bone formation. The histomorphometric analysis of MS/BS, MAR and BFR/BS was quantified to study bone dynamics.

### Statistical analysis

Data analyses were performed using SAS version 9.4 (SAS Institute, USA). Normal tests were performed to determine the normality of continuous data before appropriate statistical descriptions or analyses were chosen. Parametric Student’s *t* test or one-way ANOVA was used if the data were normally distributed. The nonparametric Wilcoxon rank sum test or Kruskal–Wallis (H test) was used if the data did not meet the requirements of the parametric test. *P* < 0.05 was considered statistically significant.

### Supplementary information

Supplementary tables and figures

Figure S1

Figure S2

Figure S3

Figure S4

Figure S5

Figure S6

Figure S7

ZOL vs CON m6A seq

ZOL vs CON mRNA seq

Original Data File

aj-checklist

## Data Availability

Data supporting the findings reported in this manuscript are available from the corresponding author upon reasonable request.
