# Supplementary material for: Exosome-targeted delivery of METTL14 regulates NFATc1 m6A methylation levels to correct osteoclast-induced bone resorption
Source: Cell Death Dis. 2023 Nov 13;14(11):738. doi: 10.1038/s41419-023-06263-4 (PMC10643436; doi:10.1038/s41419-023-06263-4)
Supplement: Supplementary file 1 — Supplementary tables and figures [file 41419_2023_6263_MOESM1_ESM.docx]

**Supplementary figures and tables**

**Fig. S1. A** The mRNA expression levels of METTL14 in human bone samples of postmenopausal women with or without bisphosphonate treatment were detected by RT-qPCR. **B, C** After transfection si-METTL14 or METTL14 into RAW264.7 cells, the mRNA and protein expression levels of METTL14 in RAW264.7 cells were detected by RT-qPCR and western blotting, respectively. **D** KEGG analysis revealing the differentially expressed genes from three intersections: osteoclast differentiation genes, MeRIP hyper genes and mRNA down genes. RT-qPCR analysis revealing the expression levels of 10 differentially expressed genes between ZOL group and ZOL+si-METTL14 group. Data are representative of three independent experiments expressed as the mean ± SD (*p < 0.05).

**Fig. S2.** The potential m6A methylation loci of NFATc1 gene on the SRAMP website.

**Fig. S3. A, B** After transfection si-NFATc1 or NFATc1 into RAW264.7 cells, the mRNA and protein expression levels of NFATc1 in RAW264.7 cells were detected by RT-qPCR and western blotting, respectively. **C** The mRNA expression levels of NFATc1 in human bone samples of postmenopausal women with or without bisphosphonate treatment were detected by RT-qPCR. Data are representative of three independent experiments expressed as the mean ± SD (*p < 0.05).

**Fig. S4.** **A, B** After transfection si-YTHDF2 or YTHDF2 into RAW264.7 cells, the mRNA and protein expression levels of YTHDF2 in RAW264.7 cells were detected by RT-qPCR and western blotting, respectively. **C** The mRNA expression levels of YTHDF2 in human bone samples of postmenopausal women with or without bisphosphonate treatment were detected by RT-qPCR. **D** After transfection YTHDF1 into RAW264.7 cells, the mRNA and protein expression levels of YTHDF1 in RAW264.7 cells were detected by RT-qPCR and western blotting, respectively. Data are representative of three independent experiments expressed as the mean ± SD (*p < 0.05).

**Fig. S5.** **A-D** After transfection si-YTHDC2 into RAW264.7 cells, the mRNA expression levels of YTHDC2 in RAW264.7 cells were detected by RT-qPCR. After ZOL stimulation, si-YTHDC2 was transfected into RAW264.7 cells and prepared for further study. TRAP staining and F-actin band staining were applied to detect osteoclast differentiation between the two groups. Scale bar: 200 μm. Histograms of the number, coverage rate and nuclei of TRAP-positive osteoclasts between the two groups. Relative expression levels of Ctsk, MMP9 and Acp5 in RAW264.7 cells between the two groups. Data are representative of three independent experiments expressed as the mean ± SD (*p < 0.05).

**Fig. S6.** **A-D** After transfection si-METTL14 2# into RAW264.7 cells, the mRNA expression levels of METTL14 in RAW264.7 cells were detected by RT-qPCR. After ZOL stimulation, si-METTL14 2# was transfected into RAW264.7 cells and prepared for further study. TRAP staining and F-actin band staining were applied to detect osteoclast differentiation between the two groups. Scale bar: 200 μm. Histograms of the number, coverage rate and nuclei of TRAP-positive osteoclasts between the two groups. Relative expression levels of Ctsk, MMP9 and Acp5 in RAW264.7 cells between the two groups. **E-H** After transfection si-NFATc1 2# into RAW264.7 cells, the mRNA expression levels of NFATc1 in RAW264.7 cells were detected by RT-qPCR. Next, si-NFATc1 2# was transfected into RAW264.7 cells and prepared for further study. TRAP staining and F-actin band staining were applied to detect osteoclast differentiation between the two groups. Scale bar: 200 μm. Histograms of the number, coverage rate and nuclei of TRAP-positive osteoclasts between the two groups. Relative expression levels of Ctsk, MMP9 and Acp5 in RAW264.7 cells between the two groups. **I-L** After transfection si-YTHDF2 2# into RAW264.7 cells, the mRNA expression levels of YTHDF2 in RAW264.7 cells were detected by RT-qPCR. Next, si-YTHDF2 2# was transfected into RAW264.7 cells and prepared for further study. TRAP staining and F-actin band staining were applied to detect osteoclast differentiation between the two groups. Scale bar: 200 μm. Histograms of the number, coverage rate and nuclei of TRAP-positive osteoclasts between the two groups. Relative expression levels of Ctsk, MMP9 and Acp5 in RAW264.7 cells between the two groups. Data are representative of three independent experiments expressed as the mean ± SD (*p < 0.05).

**Fig. S7.** The grey value analysis of western blotting throughout the whole figures.

**Table S1. Mus musculus nuclear factor of activated T cells, cytoplasmic, calcineurin dependent 1 (Nfatc1), transcript variant 5, mRNA.**

GTCACGCCGGGGACGCGCGCGAGCGCCGGGCGCACCAGTGCCGCGCGACCCCGACATGACGGGGCTGGAGCAGGACCCGGAGTTCGACTTCGATTTCCTCTTCGAGTTCGATCAGAGCGGCGGGGGCGCCGCGGCCGCAGAACACTACAGTTATGTGTCCCCTAGTGTCACCTCGACCCTGCCCCTTCCCACAGCACACTCTGCCTTGCCAGCAGCATGCCACGACCTCCAGACGTCCACCCCGGGTATCTCAGCTGTTCCTTCAGCCAATCATCCCCCCAGTTACGGAGGGGCTGTGGACAGCGGGCCTTCGGGATACTTCCTGTCCTCTGGCAACACCAGACCCAACGGGGCCCCGACTCTGGAGAGTCCGAGAATCGAGATCACCTCCTACCTGGGCCTACACCATGGCAGCGGCCAGTTTTTCCACGACGTGGAGGTGGAAGACGTACTTCCTAGCTGCAAGCGCTCACCGTCTACAGCAACCCTGCACCTGCCCAGCCTGGAAGCCTACAGAGACCCCTCCTGCCTGAGCCCAGCCAGCAGTCTCTCCTCCAGAAGCTGTAACTCTGAGGCCTCCTCCTACGAGTCCAACTACTCCTACCCATACGCGTCCCCCCAGACCTCTCCGTGGCAGTCACCCTGCGTGTCTCCCAAGACCACGGACCCGGAGGAGGGTTTTCCCCGAAGCCTGGGTGCCTGCCACCTGCTAGGATCGCCCAGGCACTCCCCATCCACCTCTCCTCGGGCAAGCATCACGGAGGAGAGCTGGCTCGGTGCCCGCGGCTCCCGGCCCACGTCCCCCTGCAACAAGCGCAAGTACAGTCTCAATGGCCGGCAGCCCTCCTGCTCACCCCACCACTCACCCACACCATCCCCCCATGGCTCCCCTCGGGTCAGTGTGACCGAAGATACCTGGCTCGGTAACACCACCCAGTATACCAGCTCTGCCATTGTGGCAGCCATCAACGCCCTGACCACCGATAGCACTCTGGACCTGGGTGATGGGGTCCCTATCAAGTCTCGAAAGACAGCACTGGAGCATGCGCCCTCTGTGGCCCTCAAAGTAGAGCCAGCTGGGGAAGACCTGGGCACCACTCCACCCACTTCTGACTTCCCACCCGAGGAGTACACCTTCCAGCACCTTCGGAAGGGTGCCTTTTGCGAGCAGTATCTGTCGGTGCCACAGGCCTCGTATCAGTGGGCGAAGCCCAAGTCTCTTTCCCCGACATCATATATGAGCCCATCCTTGCCTGCCCTTGACTGGCAGCTCCCGTCACATTCTGGTCCATACGAGCTTCGGATCGAGGTGCAGCCCAAGTCTCACCACAGGGCTCACTATGAGACGGAAGGCAGCCGGGGGGCTGTGAAGGCTTCAGCTGGAGGACACCCCATTGTGCAGCTACACGGTTACTTGGAGAATGAACCTCTCACGCTACAGCTGTTCATTGGGACGGCTGACGACCGCCTGCTGAGGCCCCACGCCTTCTACCAGGTCCACCGGATCACGGGGAAGACTGTCTCCACCACCAGCCACGAGATCATCCTGTCCAACACCAAAGTCCTGGAGATCCCGTTGCTTCCAGAAAATAACATGCGAGCCATCATCGACTGTGCTGGGATCCTGAAGCTCAGAAACTCTGATATTGAGCTGAGGAAAGGGGAGACAGACATCGGGAGGAAGAACACCAGGGTGAGGCTGGTCTTCCGAGTTCACATCCCACAGCCCAATGGCCGGACGCTGTCTCTCCAGGTGGCCTCGAACCCTATCGAGTGTTCCCAGCGGTCAGCCCAGGAGCTGCCCCTCGTGGAGAAGCAGAGCACAGACAGCTACCCAGTCATCGGCGGGAAGAAGATGGTGCTGTCTGGCCATAACTTTCTGCAAGACTCCAAAGTCATTTTCGTGGAGAAGGCTCCAGATGGCCACCACGTCTGGGAGATGGAAGCAAAGACTGACCGGGACCTGTGCAAGCCAAATTCCCTGGTGGTTGAGATACCACCTTTCCGCAACCAGAGGATAACCAGCCCCGTCCAAGTCAGTTTCTATGTCTGCAACGGGAAACGGAAGAGAAGCCAGTACCAGCGTTTCACGTACCTTCCTGCCAATGTTCCAATTATAAAGACAGAACCCACGGACGACTTTGAGCCAGCTCTGACCTGTGGACCAATGAGCCAGGGGATTAGTCCTCTGCCGAGGCCTTACTACAGCCAACAGCTCACCATGCCTCCCGACCCCGGCTCCTGCCTCGTGGCTGGCTTCGCCCCCTGCTCCCAGAGGAACACGCTGATGCCCACGCCTCCCAACGCAAGCCCGAAGCTCCACGACCTTTCCTCTCCTGCCTACACCAAGGGCCTCACCAACCCGGGCCACAGTGGTCACCTTGGACTTCAGCCACCCGCTTCGGAGGCCCCCACCATGCAGGAAGTGCCGAGACCCATGGCCATCCAACCCAACTCGCCTGAGCAGCCCCCATCCGCCAGGCTACAGCCGCAGGTGAGTCCACATCTGAACAGTAGCTGTCCCCTTGGTCGCCGACAAGTGCTCTGTCCCAACAGCCCCTCTTCTCCACTTCCATCTGCTGCCCAAGAGCCAGCCTGCTTACAGTCCTCAGCCCTCCCTCCTGACATGGGCCACCGGCAGCCACAACCGCAGAAGGTTCAAAGAAATGAATCTCCAGCCGTATTGCCAGAGGTGTGTGAGGACAGTGGCCATAACTTGGCCCCTATTCCTGTAGTGATCAAGCAAGAGCCTGAGGAATTGGACCAGTTGTACTTGGATGATGTAAATGAGATCATACGTAACGACCTCTCCAGCACGATCCCCCACTCCTAATTCAGCACATCGGAGCTGGCAGAAGCTGTGAGGAGTTGGCTCAGTGCAGACAACCGCGCTTCAGCAAGCGAGACTTTGATGAGATAAACTGGACCCACATCTGGTACCACTCAGAAATCCCAACTTACCGAACGAACGGAACGCTAGGAGCTTATGTCTGAAGAAGTAGCTCTCCAACAGGGACGGAGGAGCAGGAGAGGGCCCACTGCATCTCCTGAAAGAAACACCATCTCAAGGAACGAGAAGGGCTGGTCAGTGAGGCCAGGGGCAGACGCTGGTCCACCGTGTGGCAGACTGTCCCCAAGCCTGACCCTCCTGCCCACTGGATCAAAACACTGGAAGTGCCTTATTTAGTCTGACCACACCAGGGCATATGGAAGTGAGCACTGAATTTTCTACCATGAGAGTATTTGTGGGAGCCAAAGCTATTAAAGAACACTTCCTTAGGAGGTTTTATCTTTTGTATTAATTGATAGGATTCTGAAGAGCCTCCTGCGTATCTCTAGCTTTTCTGGCTGCCCTACACAATTTGCCTGCCTTATCCAGTGCATTTTAGAGGTCTTCCAGCCTGTCTTCTTGGCTCTCTCATAGTCAGCCCCCTCCTCTGTGTGACTGAAACCACAGGTCTGTTAATAATAGCATGCTAGGATTCTTGTTCTCAATCCAGCTTCGAACATAGTGAAGGAAGTCGAACAGCACAAGGCAGGGTCCTGGACAGAACAAAACCTGCCTGCTACAGGACAGAGCTTGTGTTTGCATGCATGTACATATAGGCATATATTTATGTATAGATATCCAAGGACAGAGGTGGCAATGAGCAGTCTGTGCCCACCAGGGGCCTTCCTTCCATGTTAGCAATAACCAGTATCCACCTTGAGAGTGCACCTCAGAGTCCAGAACCGGCTTCCCACCATCATGGTCTTTCCTTCCATCAGGGTCTACCTCTGGACAGGCAGGTGGCCTTTTCTTGCCTCACGCAGTGACTGAGCACTCCAGAGCTGTGCTCAAATGCCCATCAGCTACCAAATGGCAAAATCTGAAAGTGGTTGTAAATAACCATTACAGAATGAGTGTAGTATATTTGTTCAATTATAAGATTATTCTTTCACAGAAGCCTTATAGCTCTCTGCTTCATCTAAGAAAACAATTACCAAAAAAAACAACGTTTCTAACTGCAATCTGTGAACTGTGCGTTTTCAGATTGGTTACTGGTAACAGATAAGCTGGTGTCTGCTCTGTGTAATTAGCTGCTTACTTCAGTTACTAGCAGTGACCTATTATTTCTTATAACCAAAAAAAGCATGGTTTAATTAAAACATGTTTAATGATCGTGCCTTAGGAGTTAATGCCCCCTTATGGAACACGCCTGAATTGCACCTGTGGCTGGAAGTTTTAAGTTACTCCCAGACAGATGGACTCATGACAGGAAAAGCTCTCTCACAGGAAGATGCATCTTTAAAATTTTTGTCAGTCTGTATGATGGTGGCTTACCTTTCCCAACGCACAGAAAGAAACAACTGTCTGAAAGCATACTGAATGATTTCGCACGACTGTGAAGAGCTGGCGCGAACTGCCTTGTACACACATAGCTCCTGGCCGCCTGCAGGCTGCCTCCCGCCTGCCTCTCGTCTGTACCCCATGTTTATTAGCATCATGGAGTTGCATGAACCATTCTTAGTAGACTGTCATCTGAAAGCAAGCGTTTGATATTTGTGTCAGCTATCTTTGTAGTTAGGAGATGAATCCAATAAAGCAGTATTTTTTTTCTTTT

Blue words indicated the region of NFATc1 gene 5' or 3' untranslated regions. Red words indicated the m6A methylation sites of NFATc1 gene.

**Table S2. Primer sequences for RT-qPCR.**

| mmu-Ctsk | F: GGTCCCAGACTCCATCG | R: GCTGAAAGCCCAACAGG |
| --- | --- | --- |
| mmu-Mmp9 | F: GACGACATAGACGGCATCC | R: TGGTTCAGTTGTGGTGGTG |
| mmu-Acp5 | F: TTACTACCGTTTGCGCTTC | R: CATTTTGGGCTGCTGACT |
| mmu-ALP | F: ATATGGTAACGGGCCTGGCT | R: TCTTCTCCACCGTGGGTCTC |
| mmu-Bglap | F: CAGTCCCCAGCCCAGAT | R: GCGTTTGTAGGCGGTCTT |
| mmu-Col1a1 | F: CAGAGGCGAAGGCAACA | R: GTCCAAGGGAGCCACATC |
| mmu-Runx2 | F: CATGAGCGGCCACAGAC | R: AGGGCTTTGGGGAGGTT |
| mmu-NFATc1 | F: TATATGAGCCCATCCTTGCCT | R: GGCTGCCTTCCGTCTCATAG |
| mmu-METTL14 | F: TGGATTTGCATTTTGGCGGG | R: ATGCTATCCGCACTCTCAGC |
| mmu-YTHDF1 | F: ACAGCAAACAAAAAGCCCATTC | R: AGGAAACTCAAAAGGGCAGGT |
| mmu-YTHDF2 | F: CCTGTCGAGCATCACTCCAG | R: TGAGTCACTAGGTCACCTCTCA |
| mmu-YTHDC2 | F: GCTCATGCAATGATGACCTGT | R: AATGCCATTGTTGAGTCGCC |
| mmu-Epha2 | F: ACCATTGCTGTCGCTGTCTCC | R: CACCAGCCACTCGCCATCC |
| mmu-Fos | F: TCCCGTGGTCACCTGTACTCC | R: TGCTGCTGCTGCCCTTTCG |
| mmu-Jun | F: AGTCCCTTCTCCCGCCTTCC | R: GGTAGCCGCTCGCCTATTTCC |
| mmu-Mapk3 | F: AAGGAGCGGCTGAAGGAGTTG | R: AGGAGCAGGTAGGAGCAGGAC |
| mmu-Ncf1 | F: GCTGGTGGGTGGTCAGGAAAG | R: TCTGTGCGTTGCGGATGGTC |
| mmu-Plcg2 | F: TGGAGCAAGACCGCAGACAAG | R: GGTGCCGTAGAGGATGGTGAAG |
| mmu-Sirpa | F: GAGGACATCCAGCCAGCCAATC | R: GCCACGGAGGGAAGACAAAGG |
| mmu-Spi1 | F: GGGCATCCAGAAGGGCAACC | R: GCCGCTGAACTGGTAGGTGAG |
| mmu-Tgfbr2 | F: ACGACTTGACCTGTTGCCTGTG | R: CCCACCTGCCCGCTGTTG |
| mmu-Tyrobp | F: TGCGACTGTTCTTCCGTGAGC | R: AGTACACAGCCAGGGCAATCAG |
| mmu-GAPDH | F: GTTGCCATCAACGACCCCTT | R: TCCACGACATACTCAGCACC |
| has-NFATc1 | F: GTCCCACCACCGAGCCCACTACG | R: GACCATCTTCTTCCCGCCCACGAC |
| hsa-METTL14 | F: TTTCTCTGGTGTGGTTCTGG | R: AAGTCTTAGTCTTCCCAGGATTG |
| has-YTHDF2 | F: TGTTGGAGAAGCTTCGGTCC | R: ACCCGGCCATGTTTCAGATT |
| has-GAPDH | F: AATCCCATCACCATCTTCCAG | R: AAATGAGCCCCAGCCTTC |

**Table S3. The siRNA sequences used in this study.**

| mmu-NFATc1 siRNA | 5’-CCCGUCCAAGUCAGUUUCUAU-3’ |
| --- | --- |
| mmu-NFATc1 siRNA 2# | 5’-CUCUCACGCUACAGCUGUU-3’ |
| mmu-METTL14 siRNA | 5’-GGAUGAGUUAAUAGCUAAA-3’ |
| mmu-METTL14 siRNA 2# | 5’-GCAGCACCUCGGUCAUUUATT-3’ |
| mmu-YTHDF2 siRNA | 5’-GACCAAGAAUGGCAUUGCA-3’ |
| mmu-YTHDF2 siRNA 2# | 5’-CCAUGAUUGAUGGACAGUCAGCUUU-3’ |
| mmu-YTHDC2 siRNA | 5’-CCUGUUAGAUGAUUGCUUUTTT-3’ |

**Table S4. Specific primers for the m6A-modified RNA fragments** **in** **NFATc1.**

| Site 1 | F: ACCACGTCTGGGAGATGGAA | R: TGGTTATCCTCTGGTTGCGGAA |
| --- | --- | --- |
| Site 2 | F: GAAGAGAAGCCAGTACCAGCG | R: TGGCTGTAGTAAGGCCTCGG |
| Site 3 | F: TTCCTCTCCTGCCTACACCAAGG | R: ATGGGTCTCGGCACTTCCT |
| Site 4 | F: CACATCGGAGCTGGCAGAAG | R: GTGGTACCAGATGTGGGTCCAG |
| Site 5 | F: ACACCATCTCAAGGAACGAGAAGG | R: TGCCCTGGTGTGGTCAGACTAAA |
| Site 6 | F: GGAAGTCGAACAGCACAAGGC | R: CATTGCCACCTCTGTCCTTGG |
| Site 7 | F: TTCCATCAGGGTCTACCTCTGGAC | R: AGCTGATGGGCATTTGAGCAC |
| Site 8 | F: CACAGAAGCCTTATAGCTCTCTGC | R: CTAATTACACAGAGCAGACACCAGC |
| Site 9-10 | F: GAATTGCACCTGTGGCTGGAAG | R: CGGCCAGGAGCTATGTGTGT |
