# Supplementary figures and images for: Exosome-targeted delivery of METTL14 regulates NFATc1 m6A methylation levels to correct osteoclast-induced bone resorption
Source: Cell Death Dis. 2023 Nov 13;14(11):738. doi: 10.1038/s41419-023-06263-4 (PMC10643436; doi:10.1038/s41419-023-06263-4)

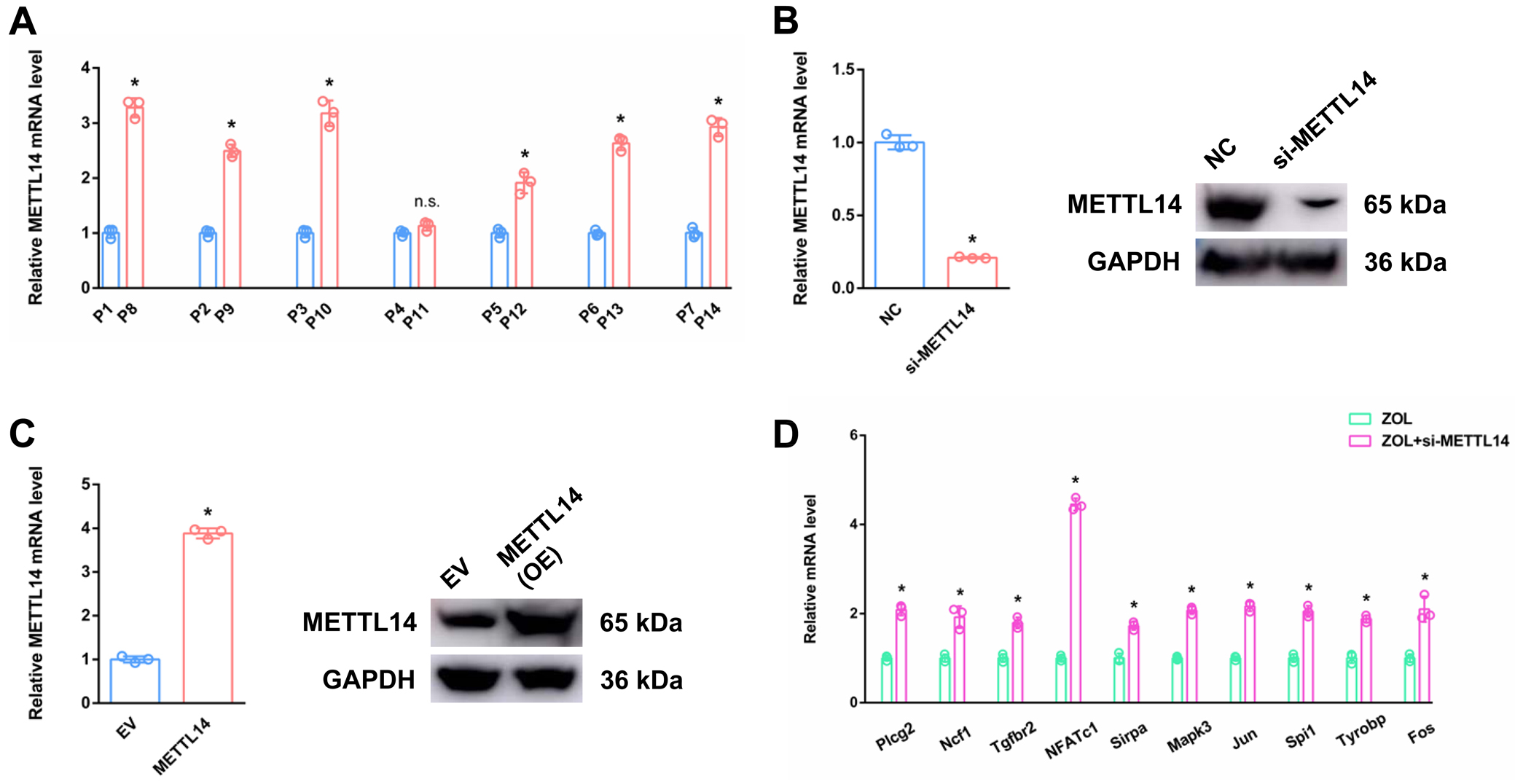

Supplement: Supplementary file 2 — Figure S1 [file 41419_2023_6263_MOESM2_ESM.jpg]

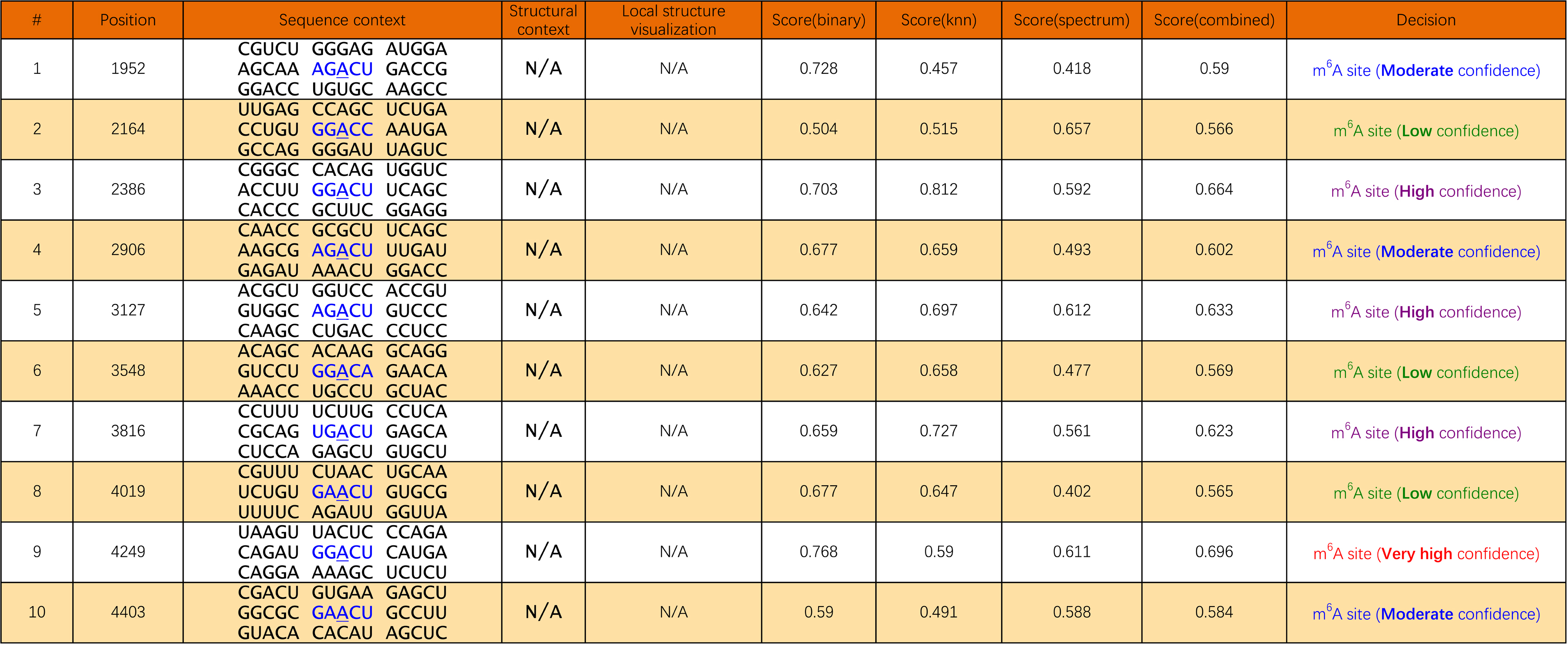

Supplement: Supplementary file 3 — Figure S2 [file 41419_2023_6263_MOESM3_ESM.jpg]

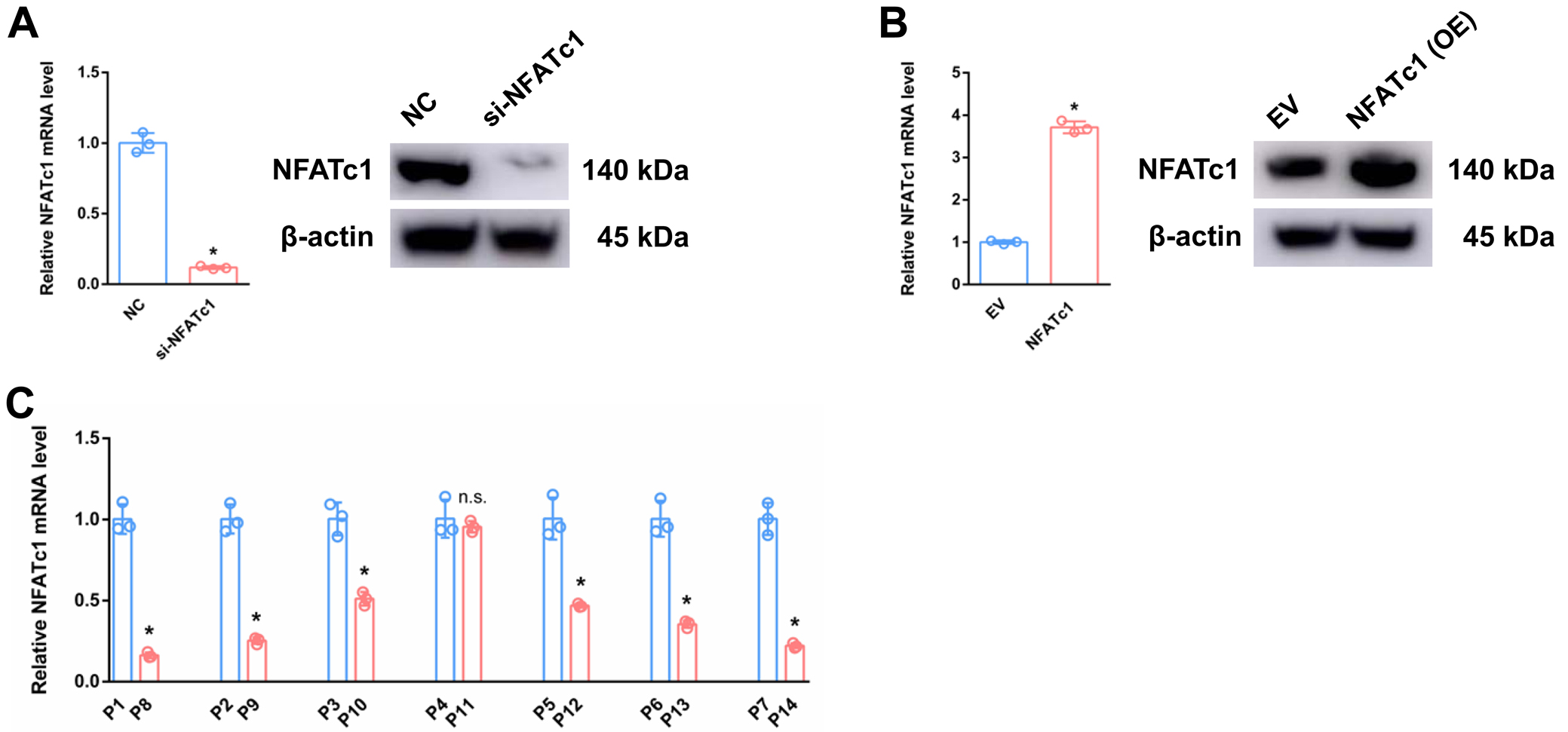

Supplement: Supplementary file 4 — Figure S3 [file 41419_2023_6263_MOESM4_ESM.jpg]

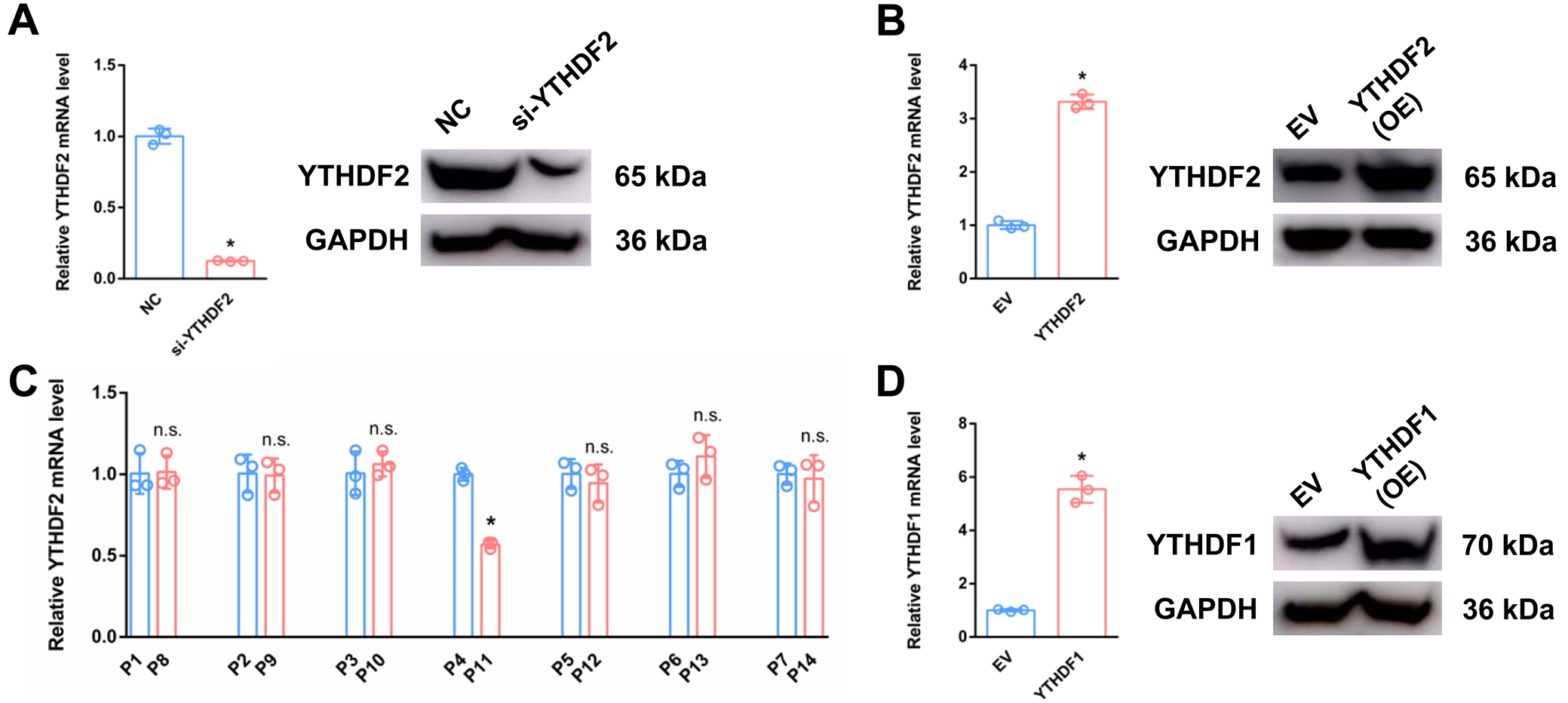

Supplement: Supplementary file 5 — Figure S4 [file 41419_2023_6263_MOESM5_ESM.jpg]

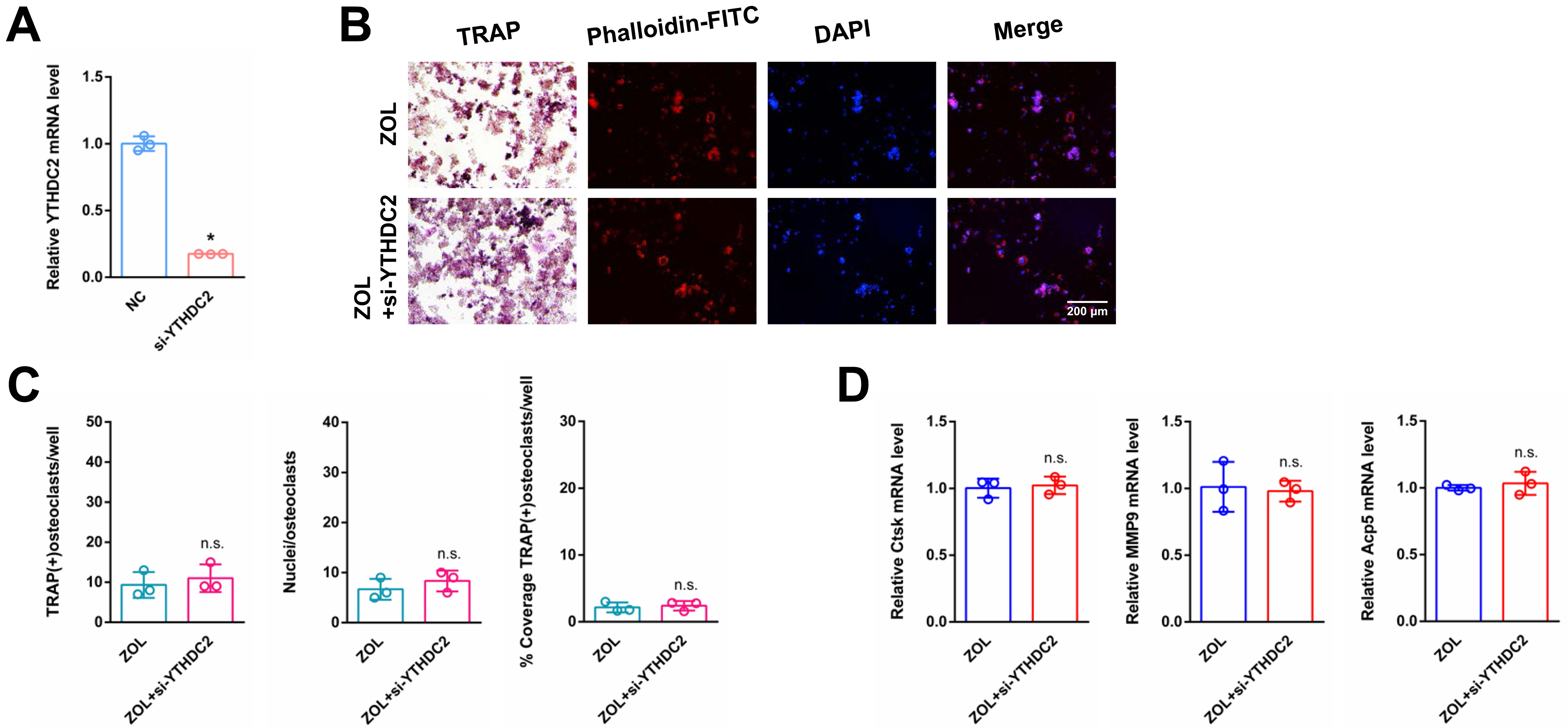

Supplement: Supplementary file 6 — Figure S5 [file 41419_2023_6263_MOESM6_ESM.jpg]

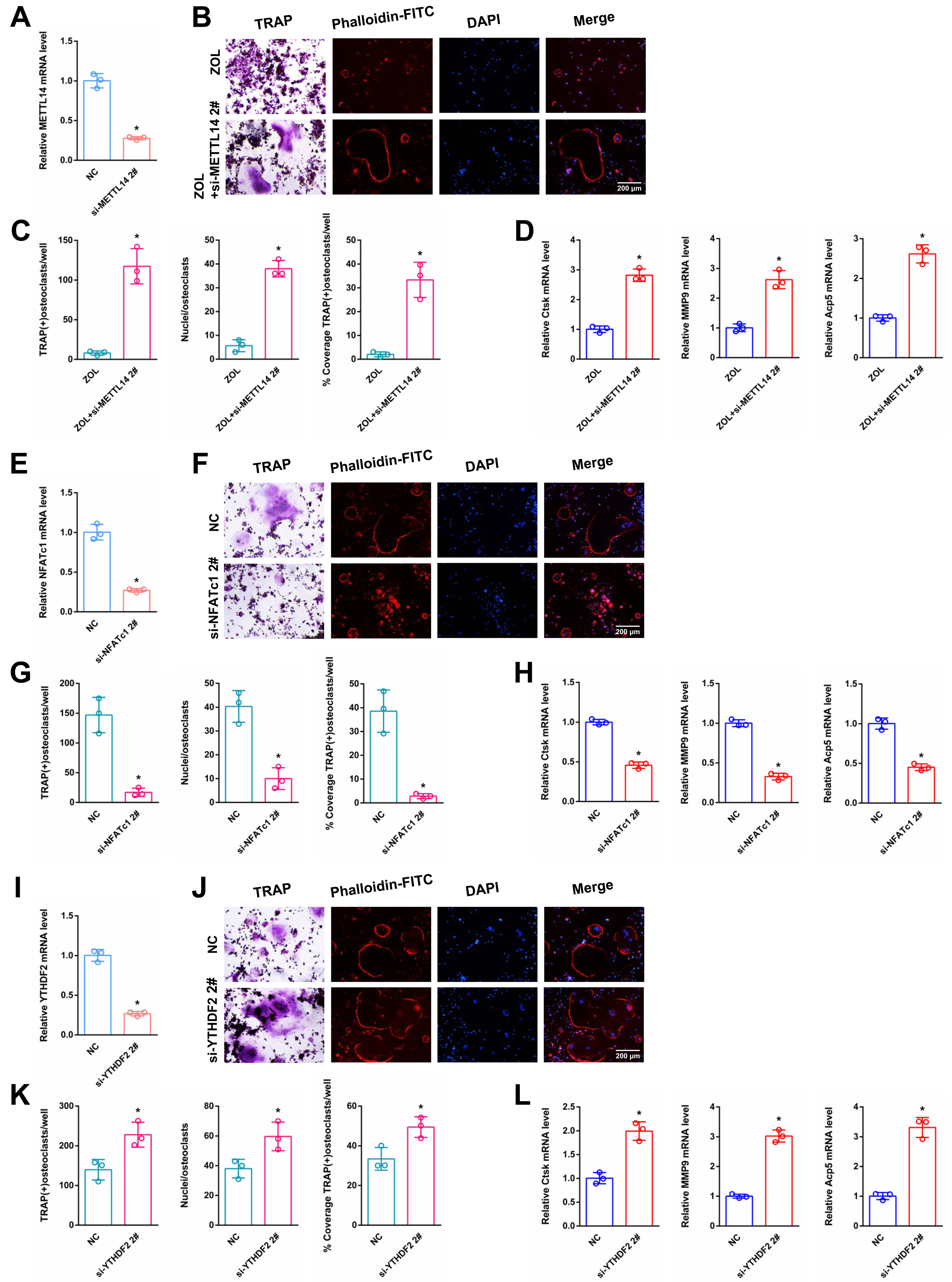

Supplement: Supplementary file 7 — Figure S6 [file 41419_2023_6263_MOESM7_ESM.jpg]

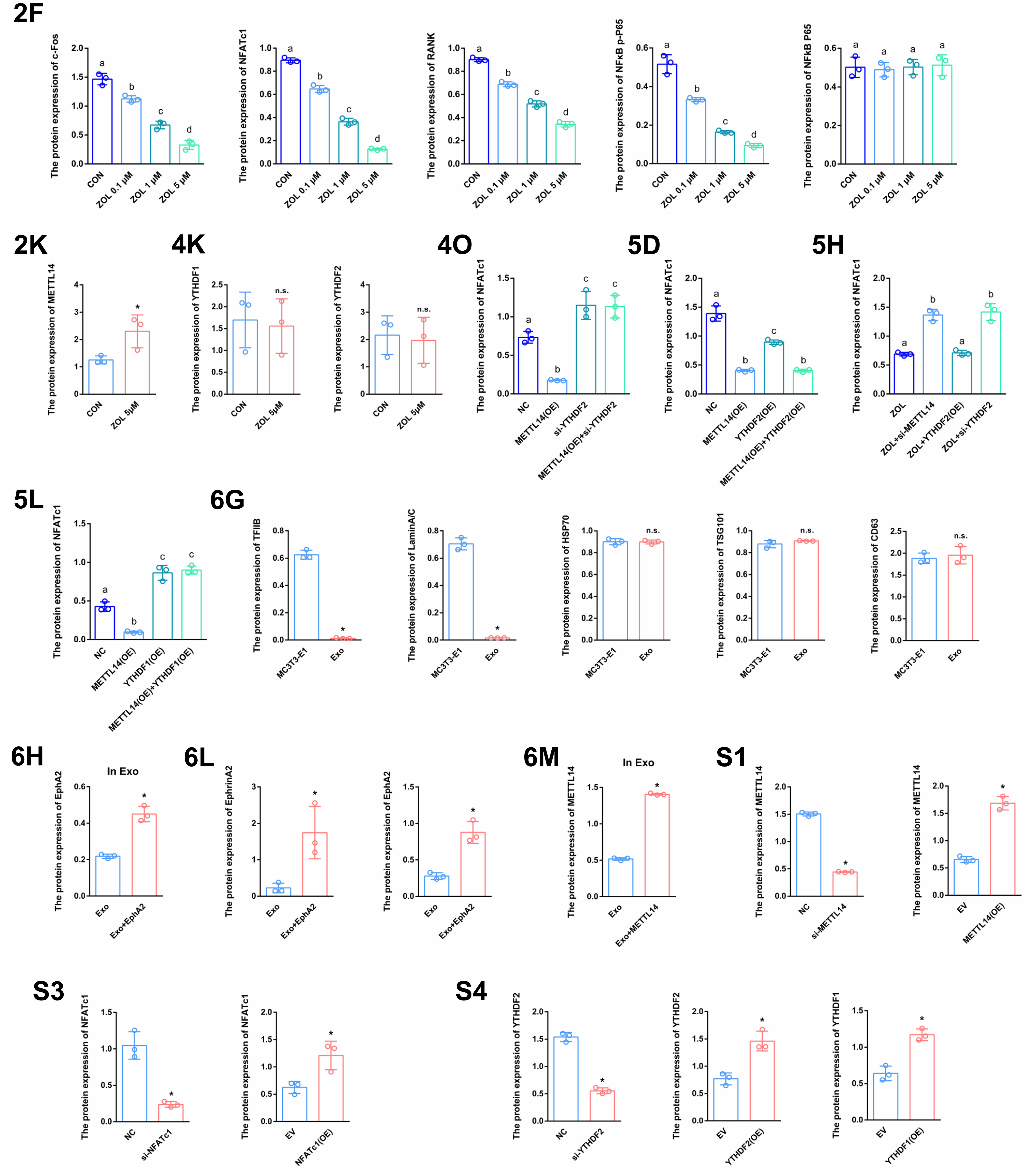

Supplement: Supplementary file 8 — Figure S7 [file 41419_2023_6263_MOESM8_ESM.jpg]
