# Supplementary material for: Exosome-targeted delivery of METTL14 regulates NFATc1 m6A methylation levels to correct osteoclast-induced bone resorption
Source: Cell Death Dis. 2023 Nov 13;14(11):738. doi: 10.1038/s41419-023-06263-4 (PMC10643436; doi:10.1038/s41419-023-06263-4)

**Supplementary Figures**

**Fig. 2F**

β-actin c-Fos


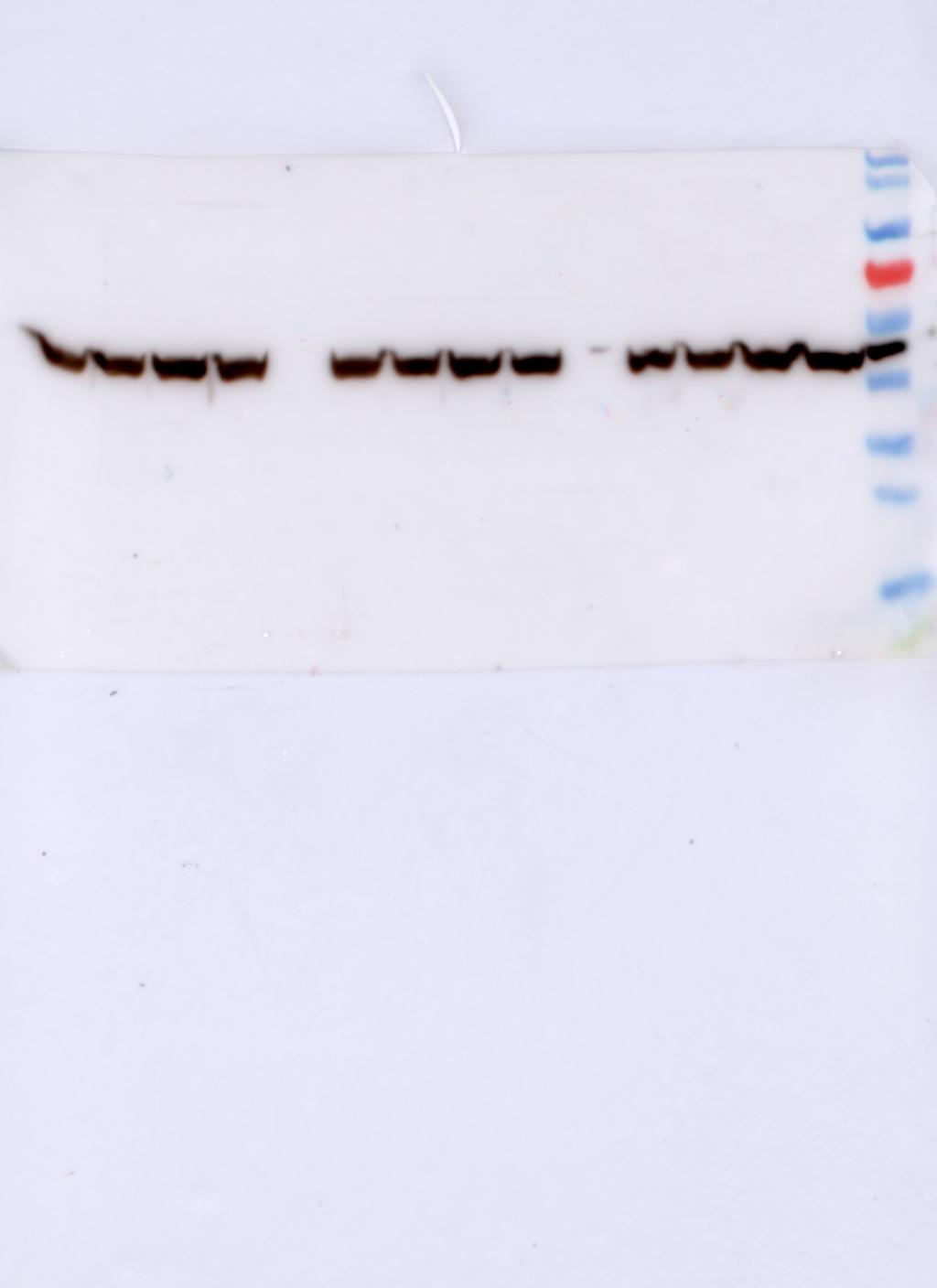

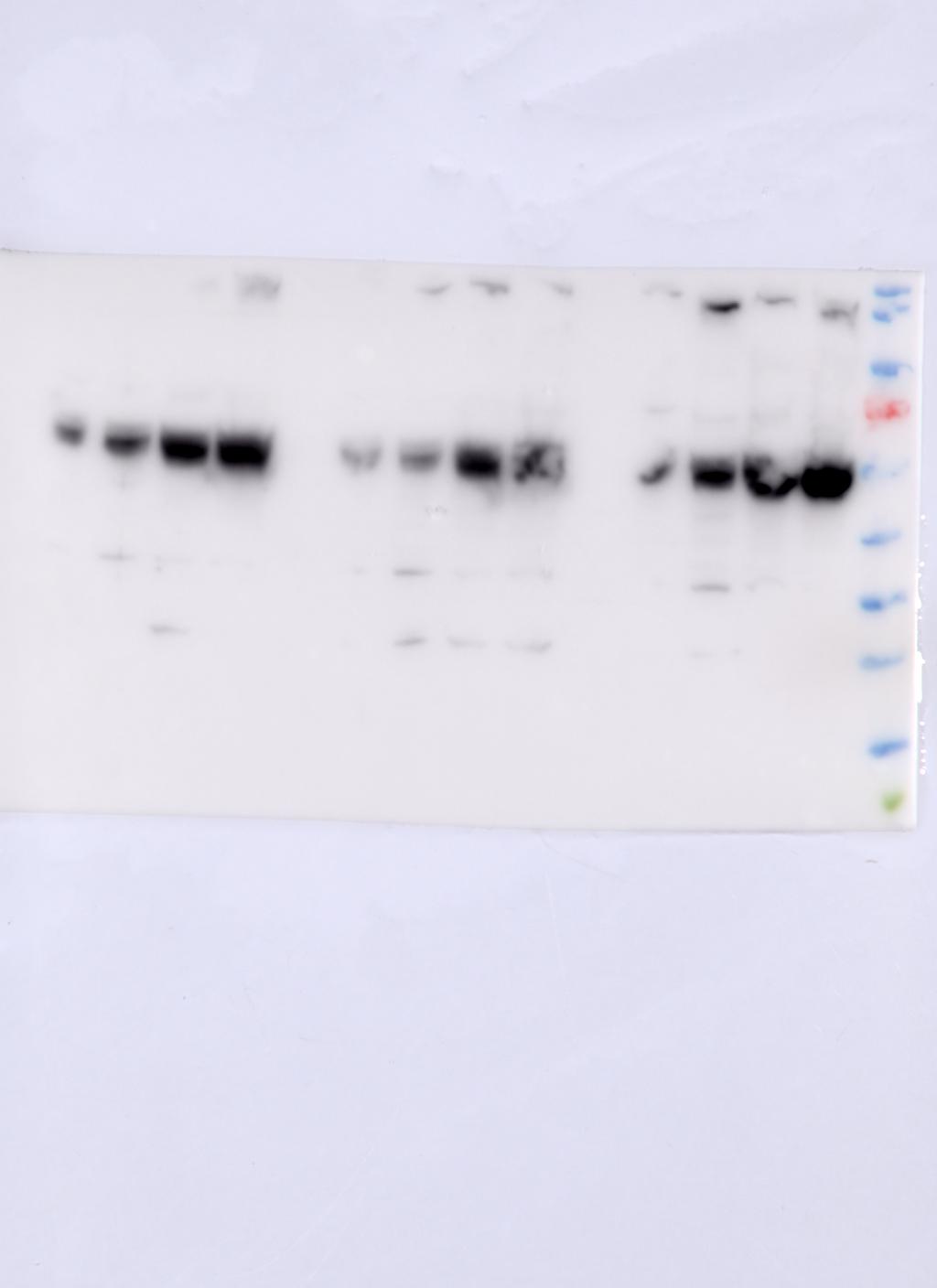


NFATc1 RANK


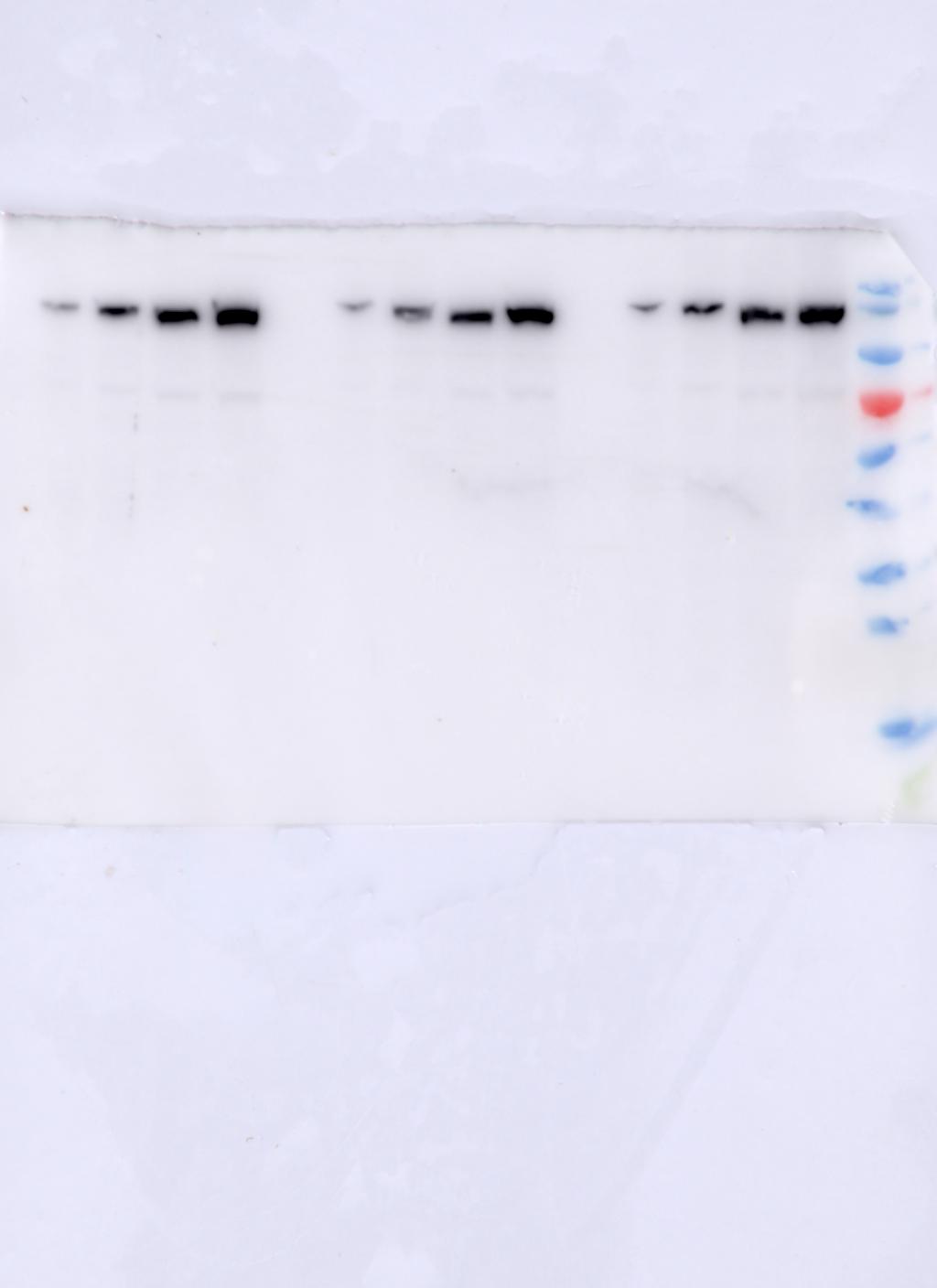

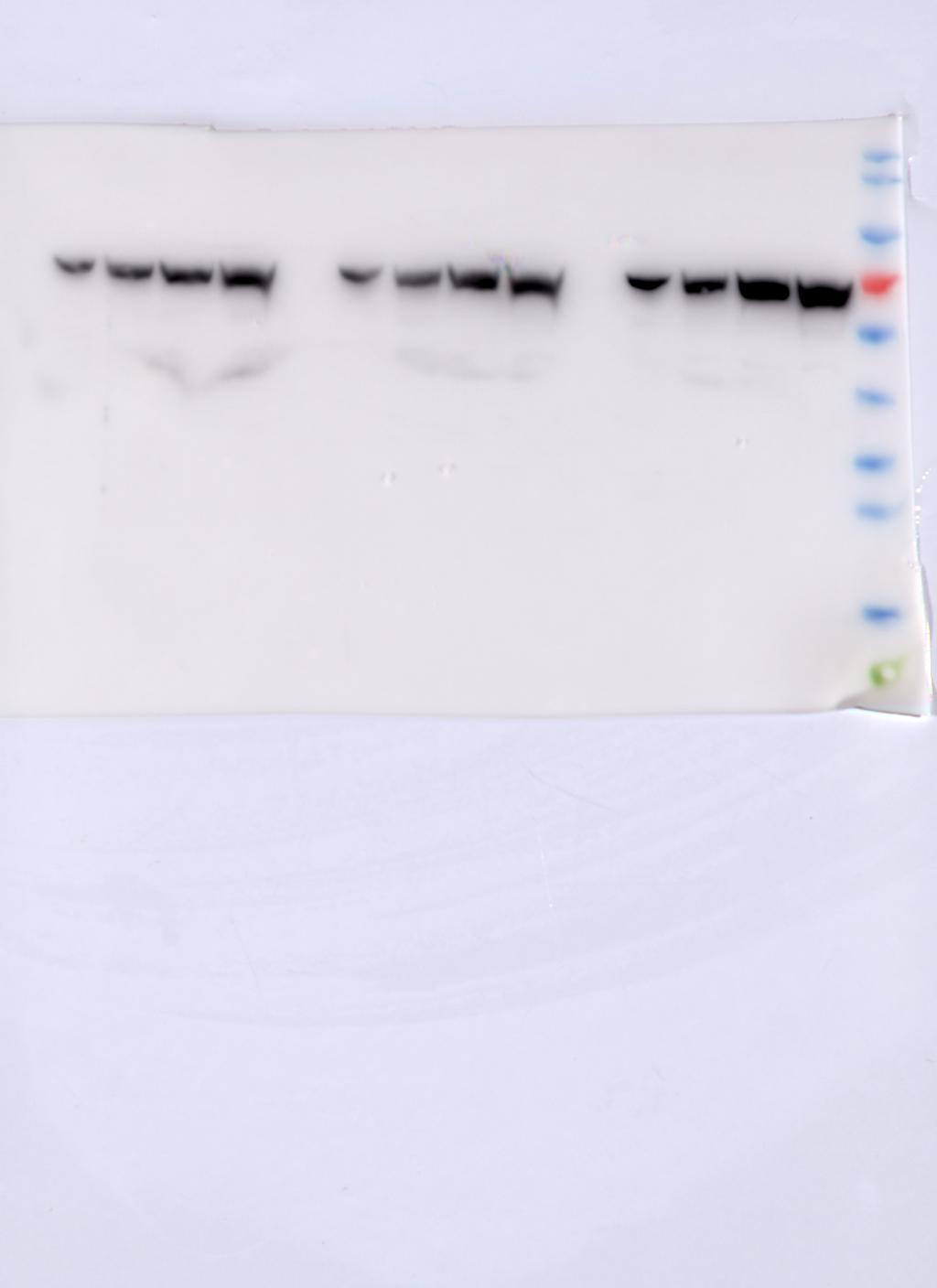


NFκB P65 NFκB p-P65


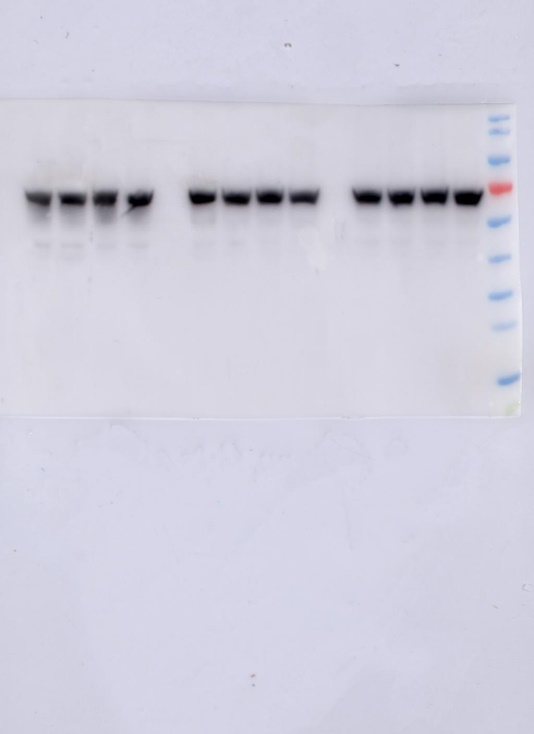

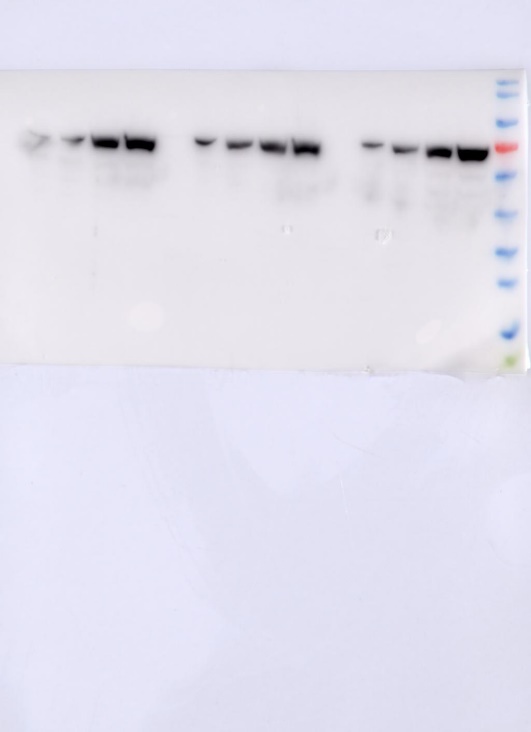


**Fig. 2K**

GAPDH METTL14


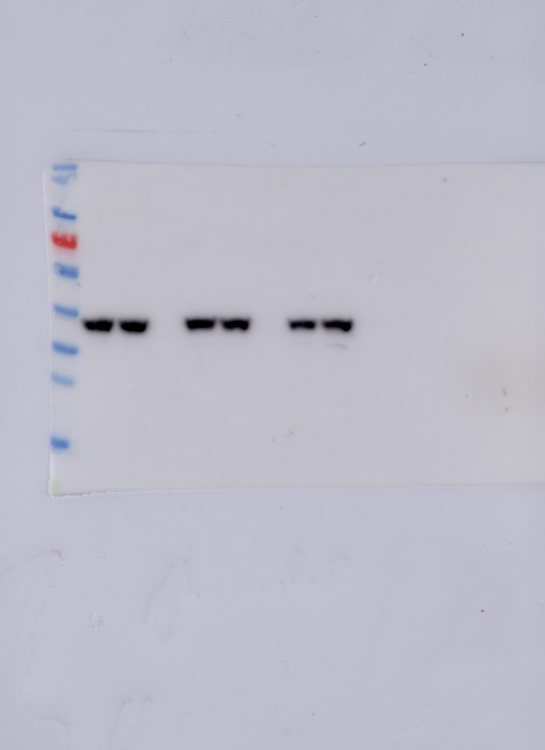

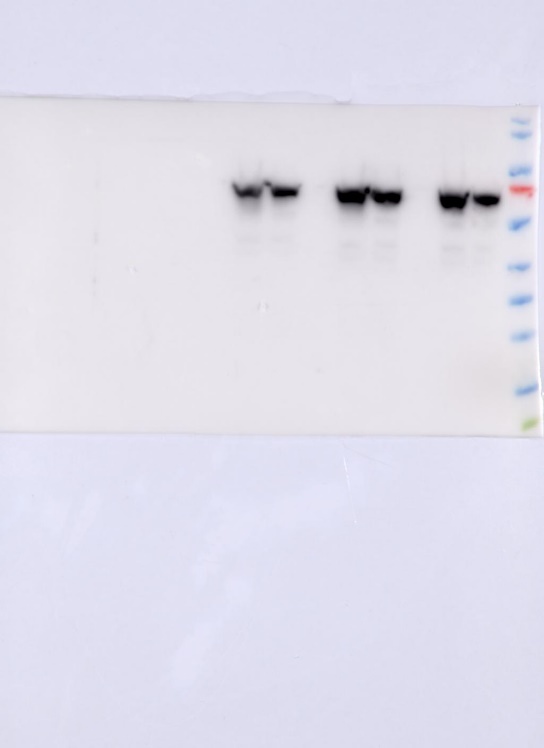


**Fig. 4K**

GAPDH YTHDF1


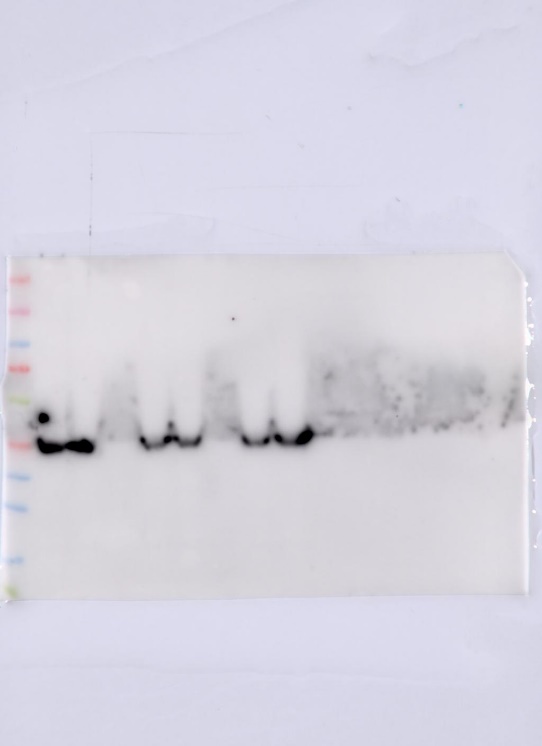

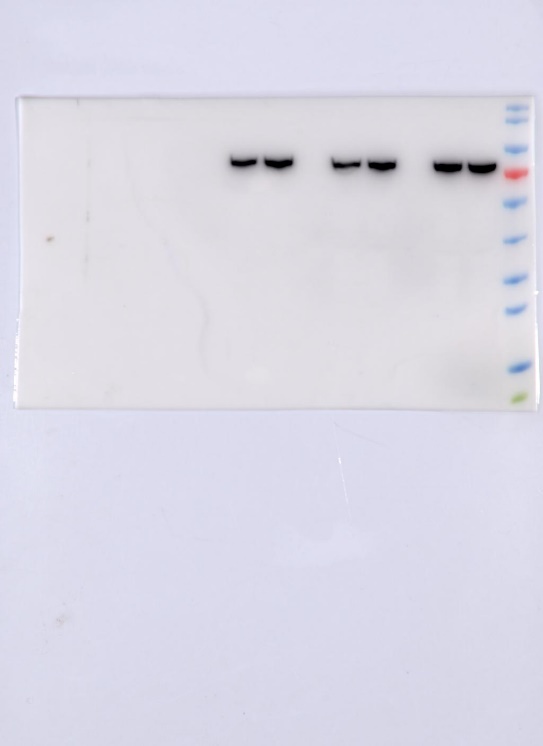


YTHDF2


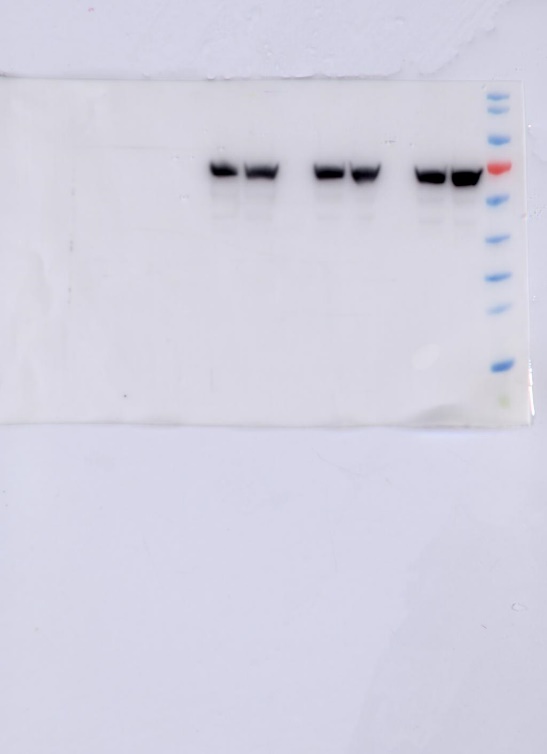


**Fig. 4O**

β-actin NFATc1


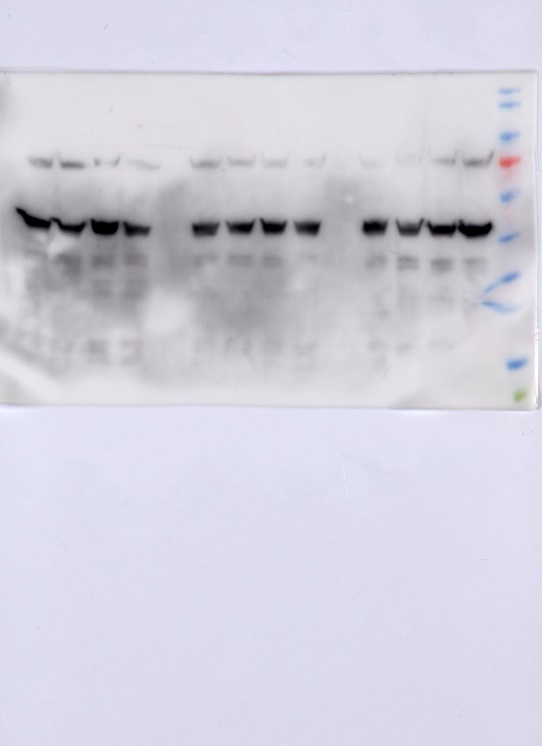

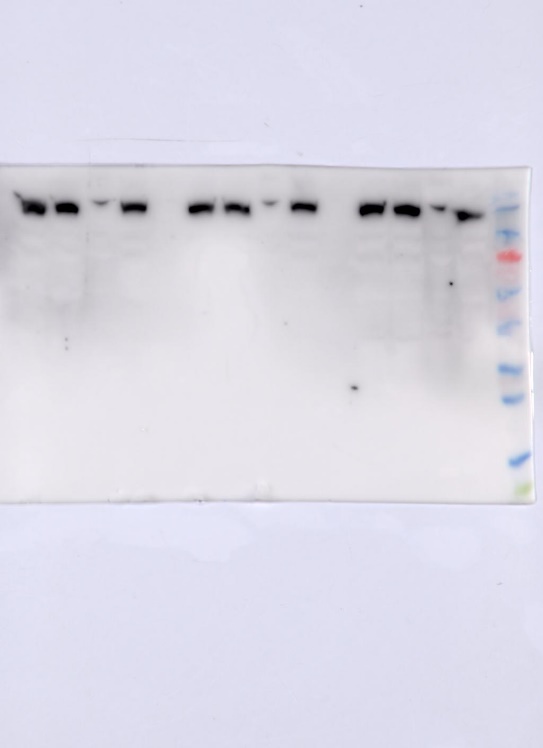


**Fig. 5D**

β-actin NFATc1


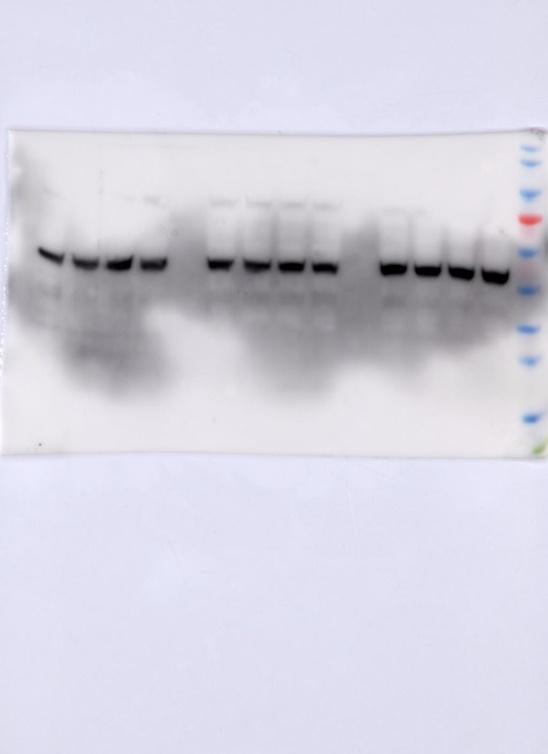

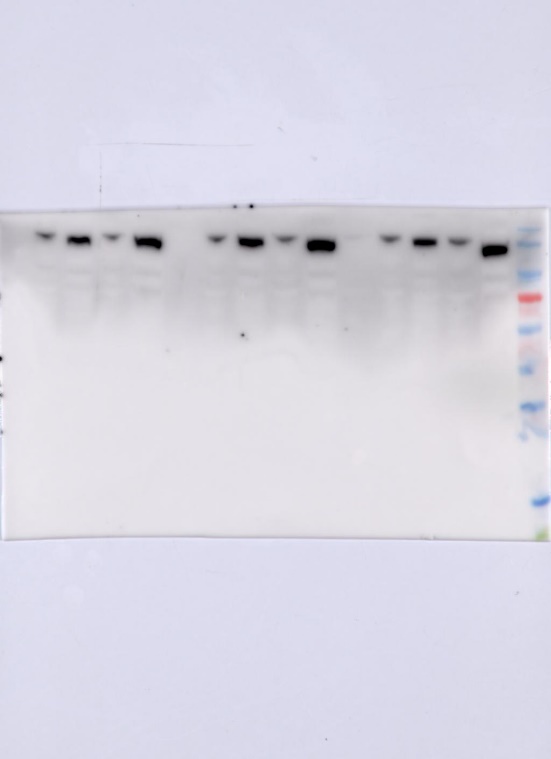


**Fig. 5H**

β-actin NFATc1


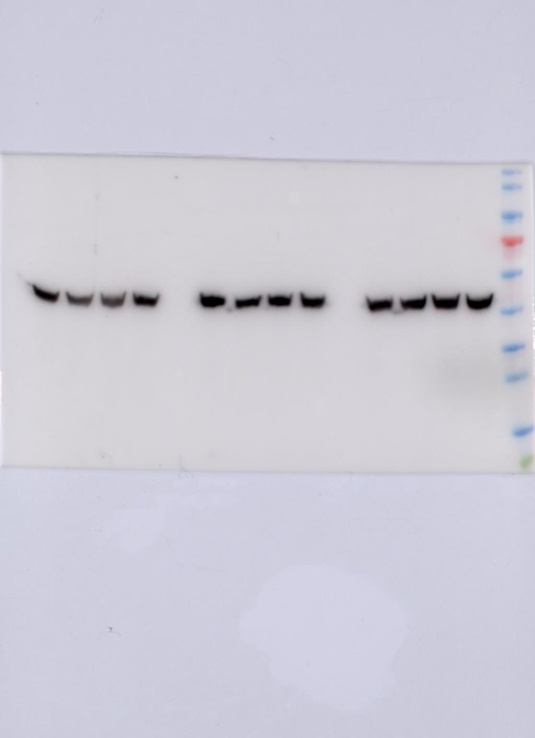

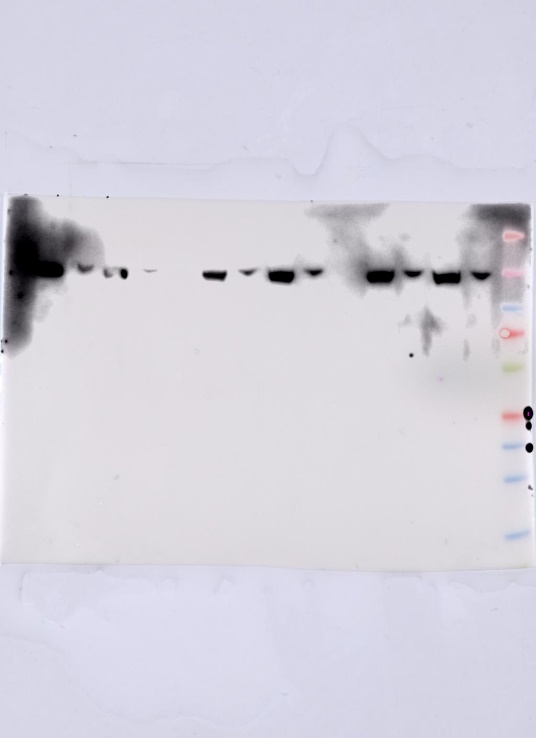


**Fig. 5L**

β-actin NFATc1


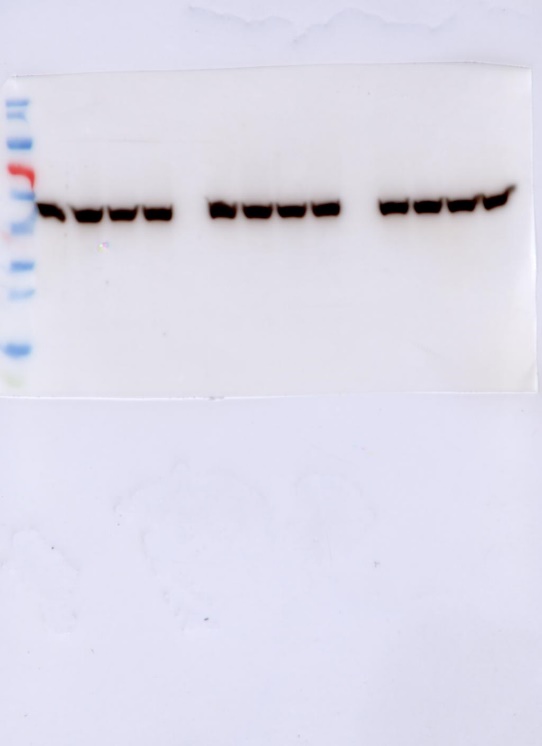

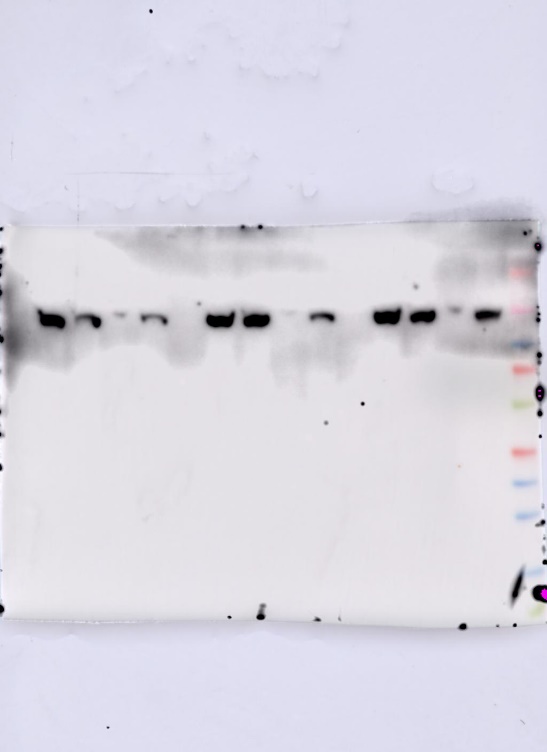


**Fig. 6G**

HSP70 β-actin


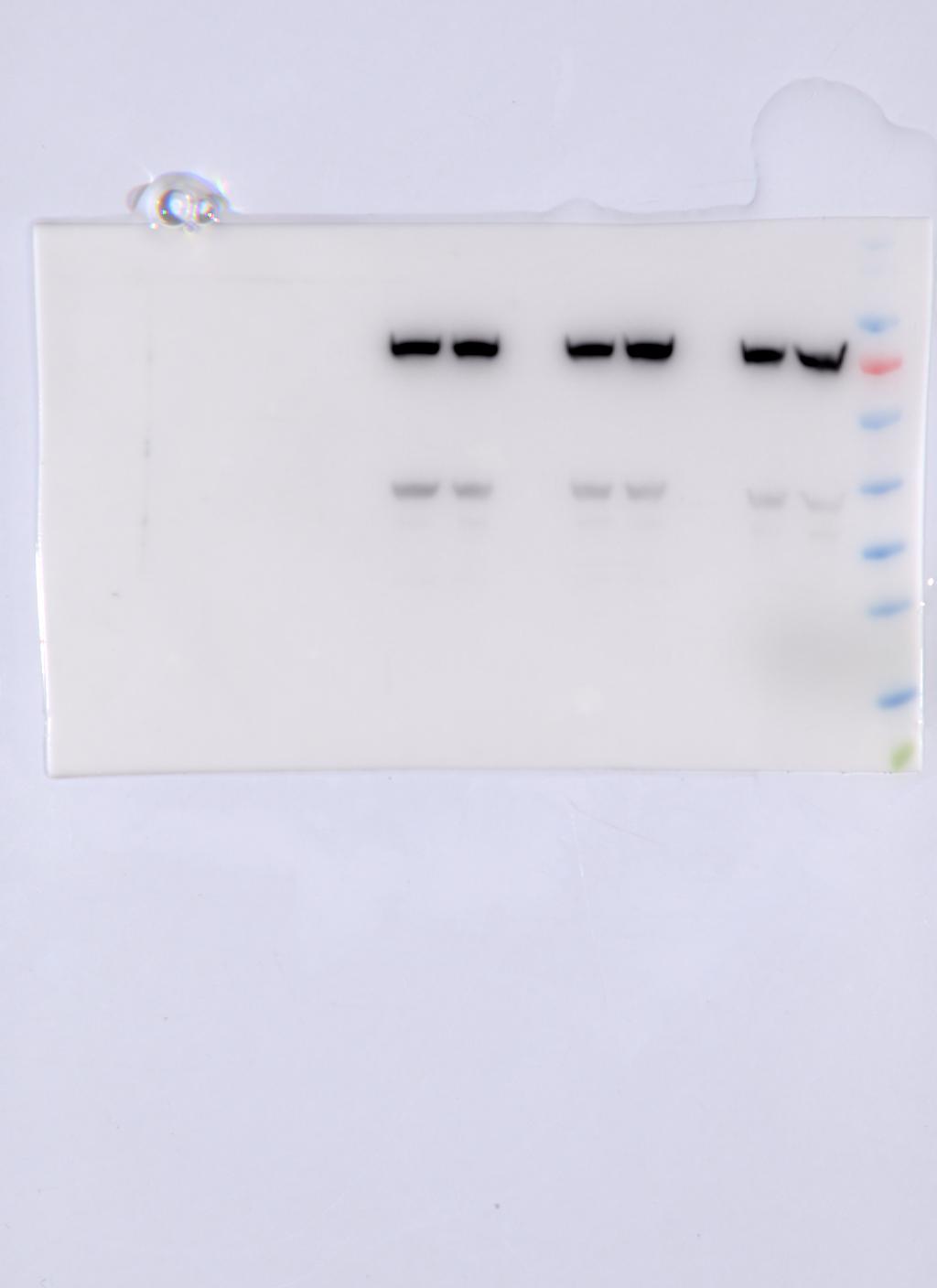

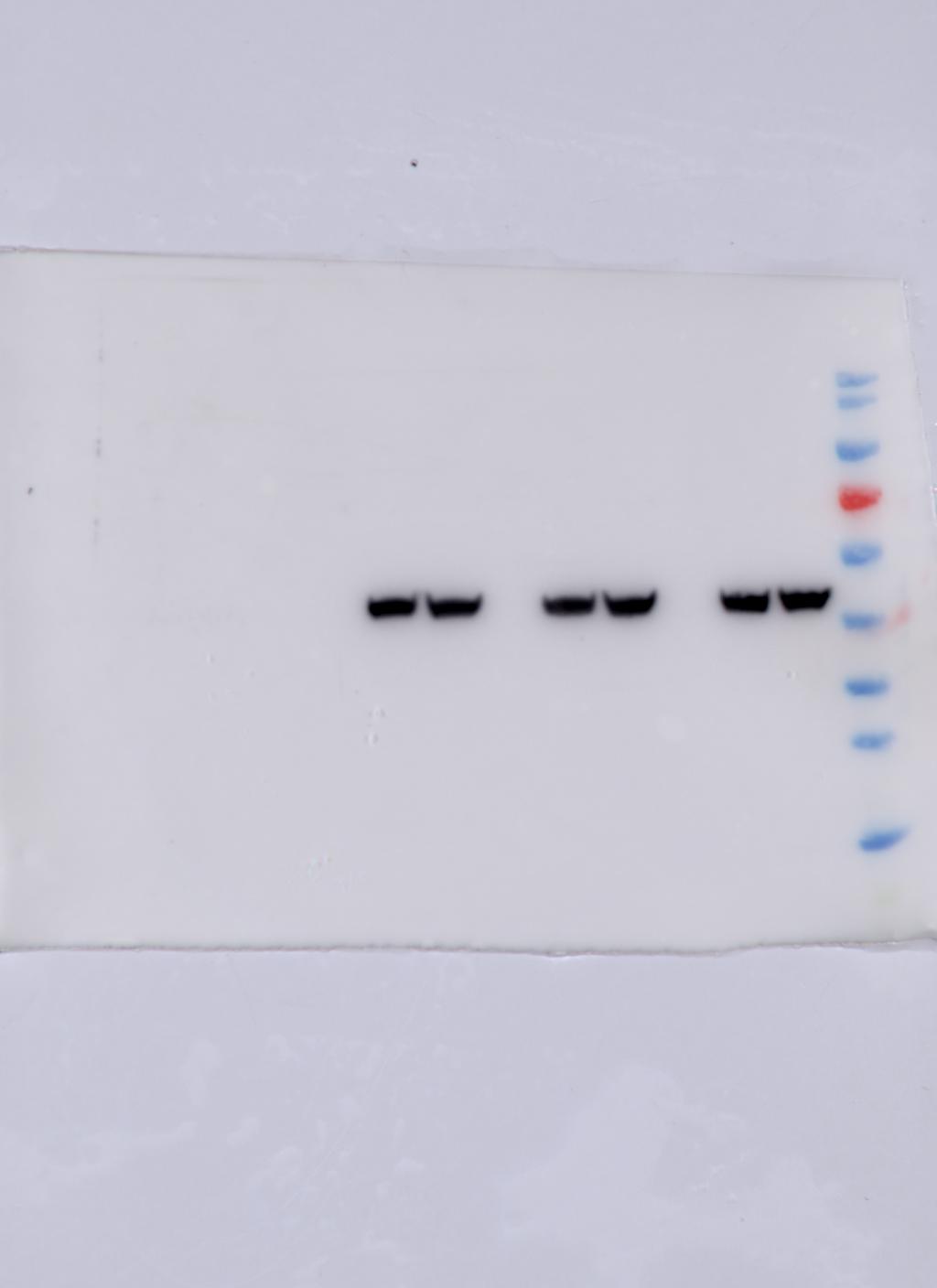


TSG101 CD63


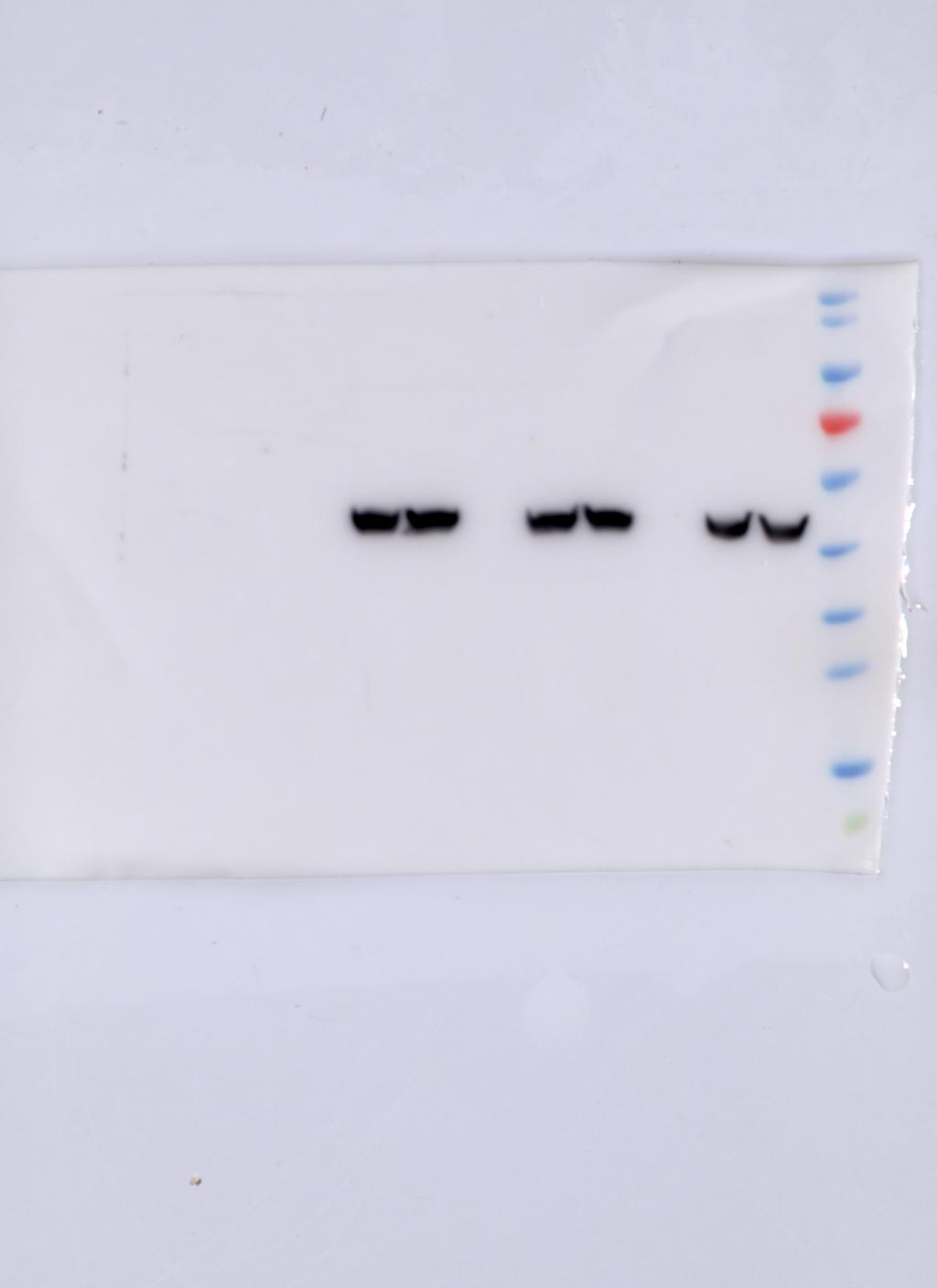

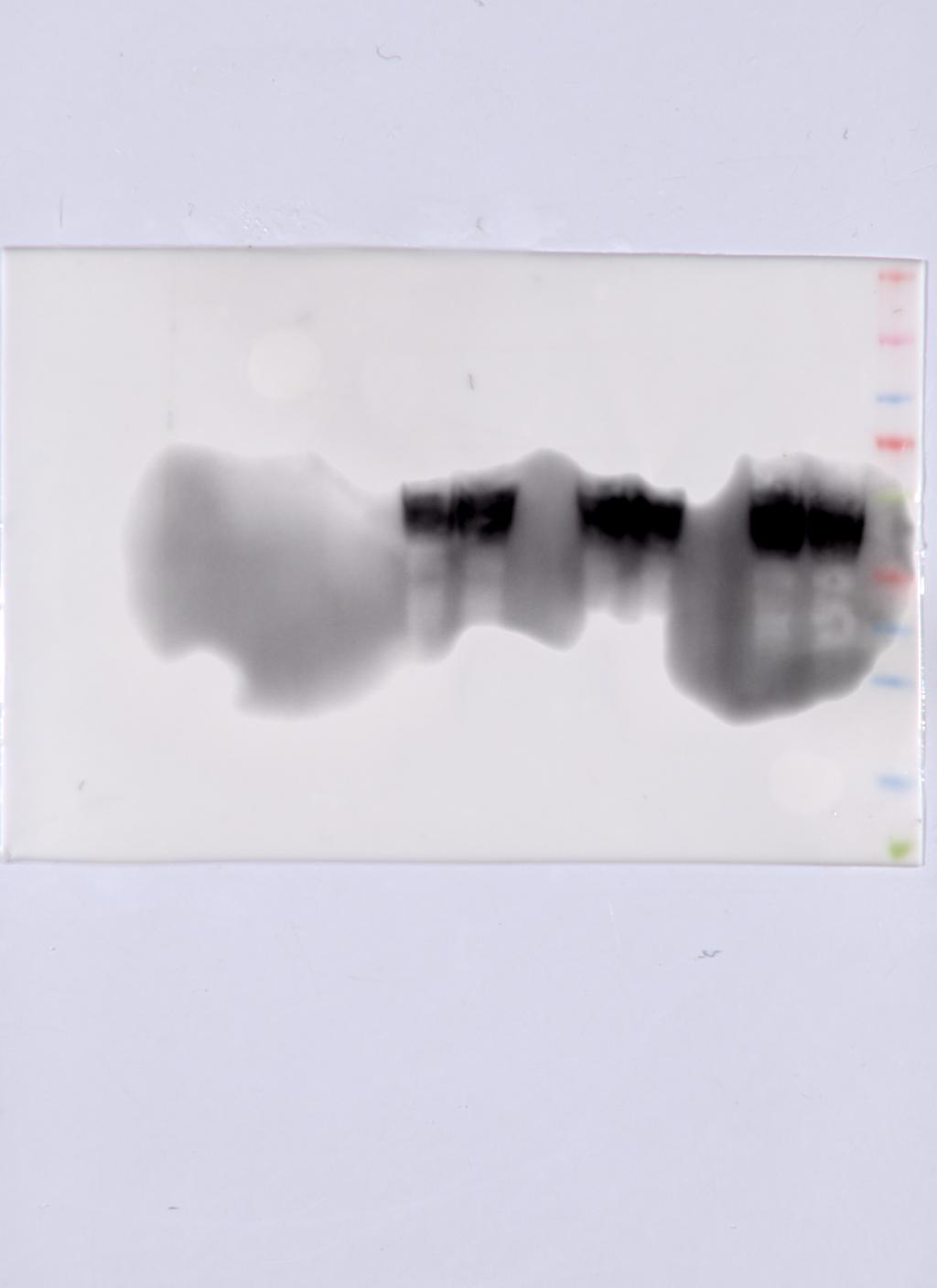


TFIIB LaminA/C


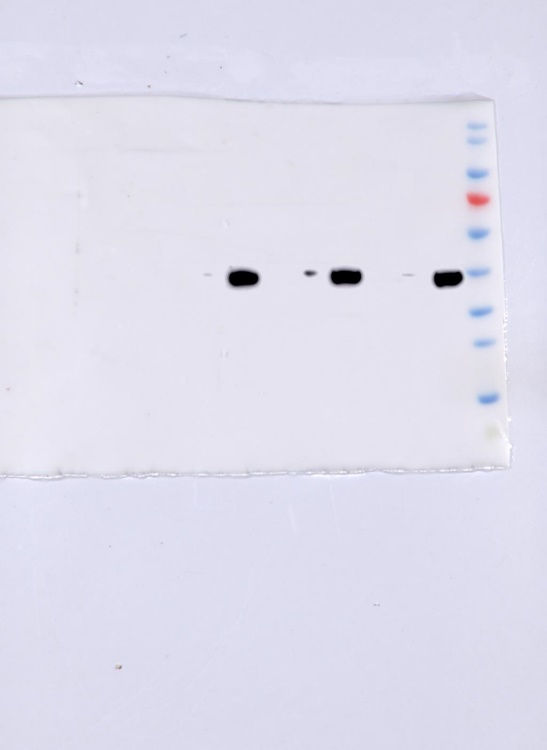

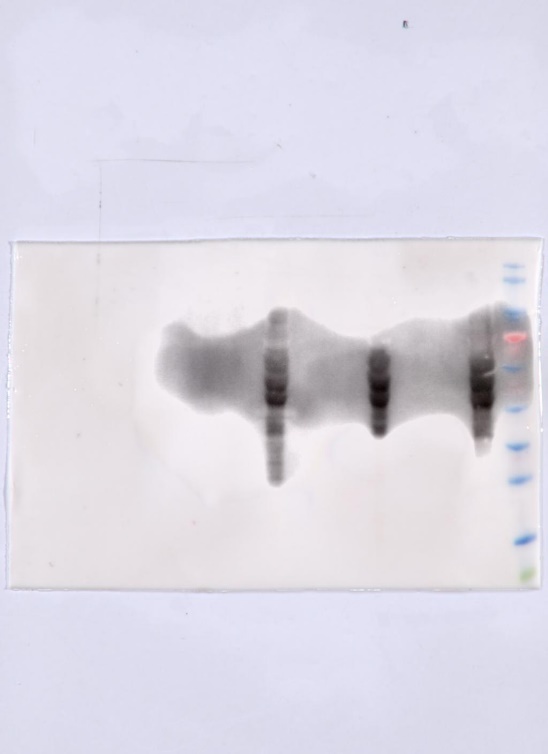


**Fig. 6H**

EphA2 β-actin


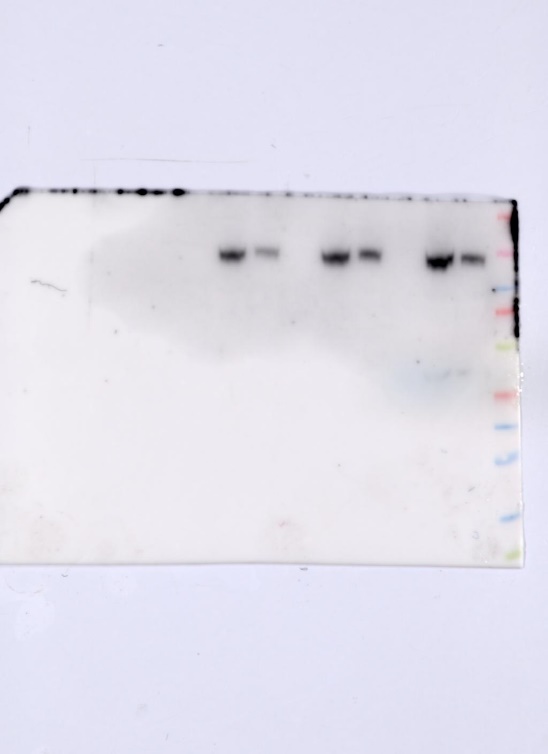

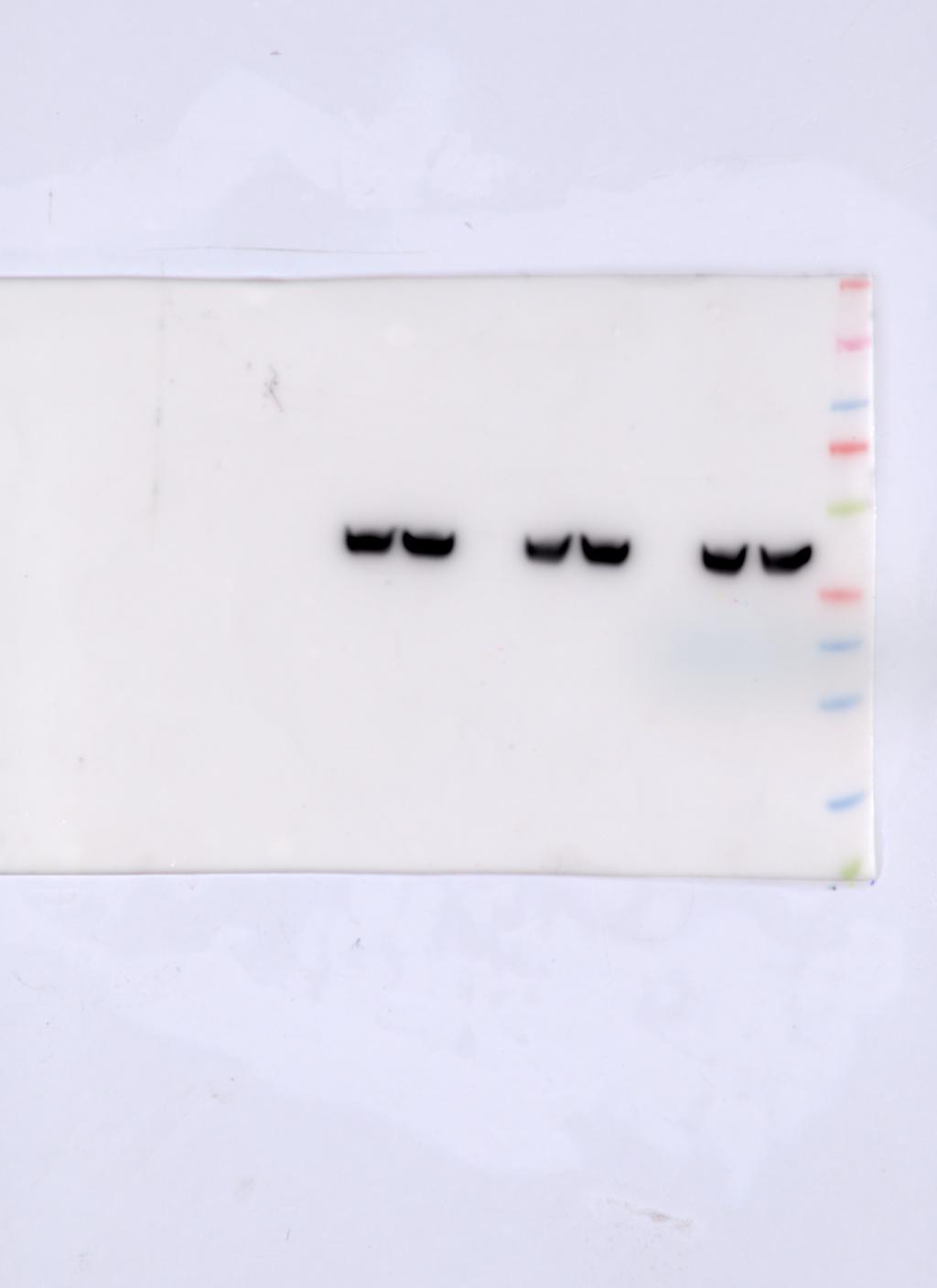


**Fig. 6L**

IB-EphrinA2 IP-EphA2 IB-EphrinA2 lysates


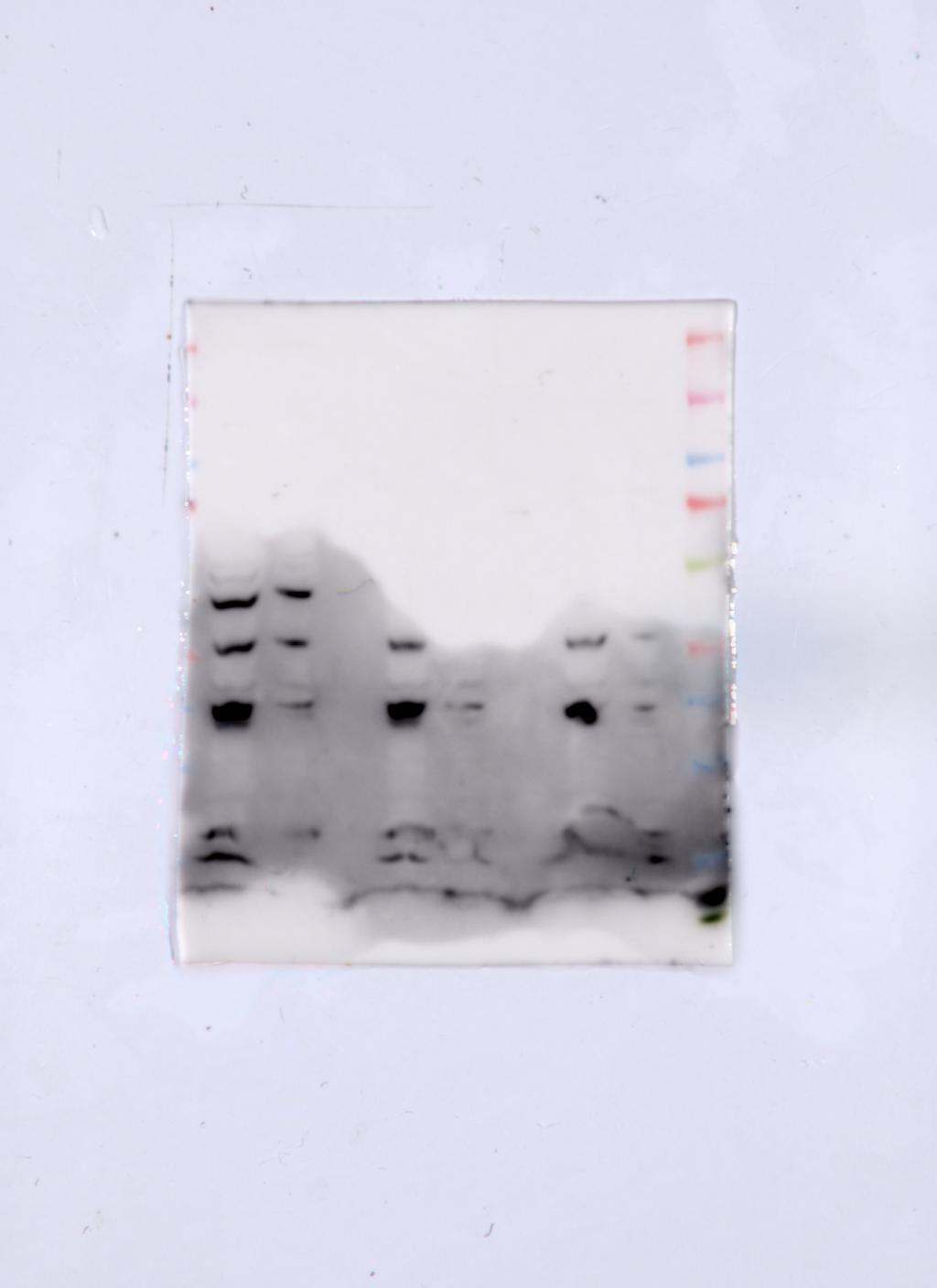

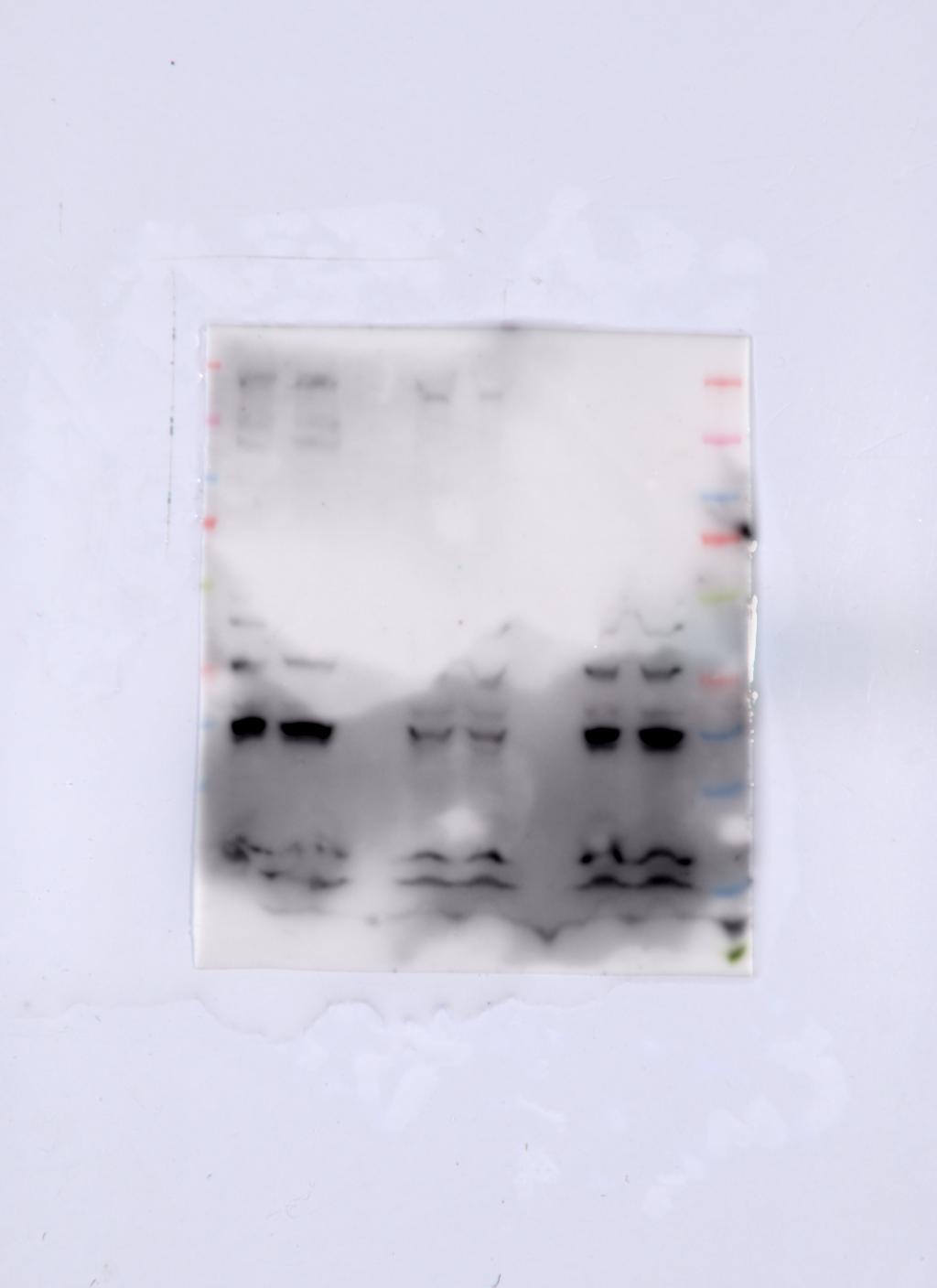


IB-EphA2 IP-EphrinA2 IB-EphA2 lysates


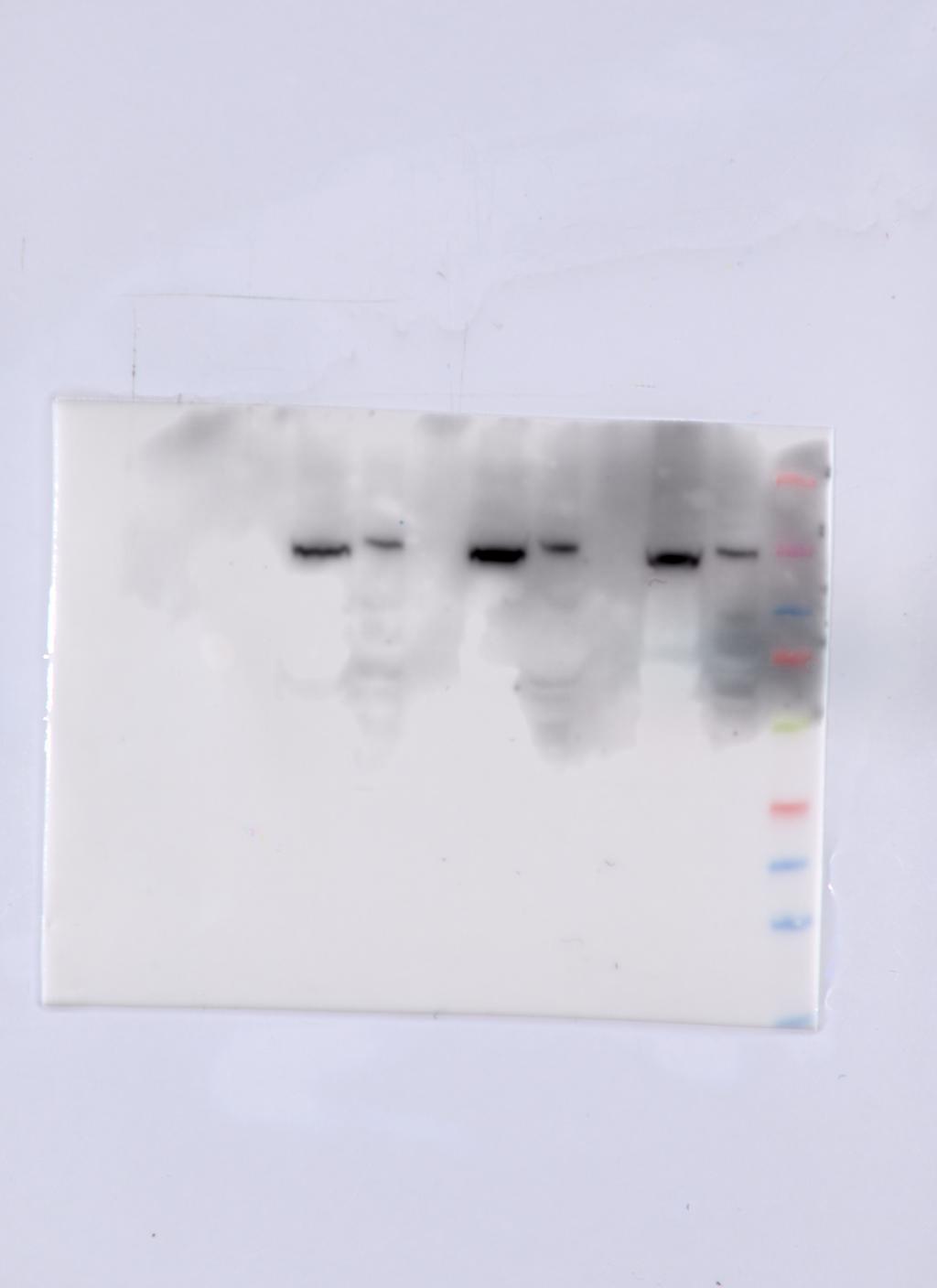

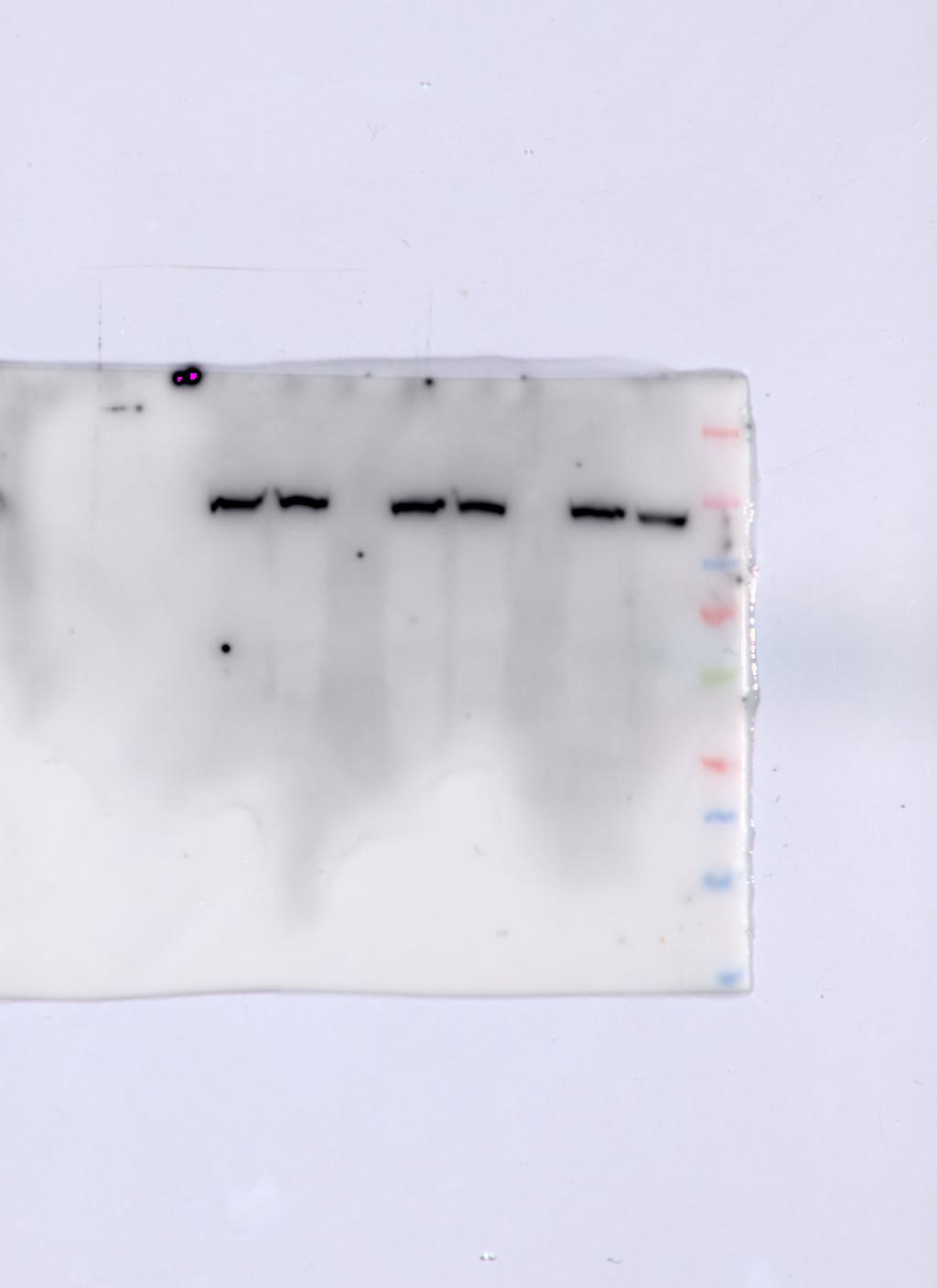


**Fig. 6M**

METTL14 β-actin


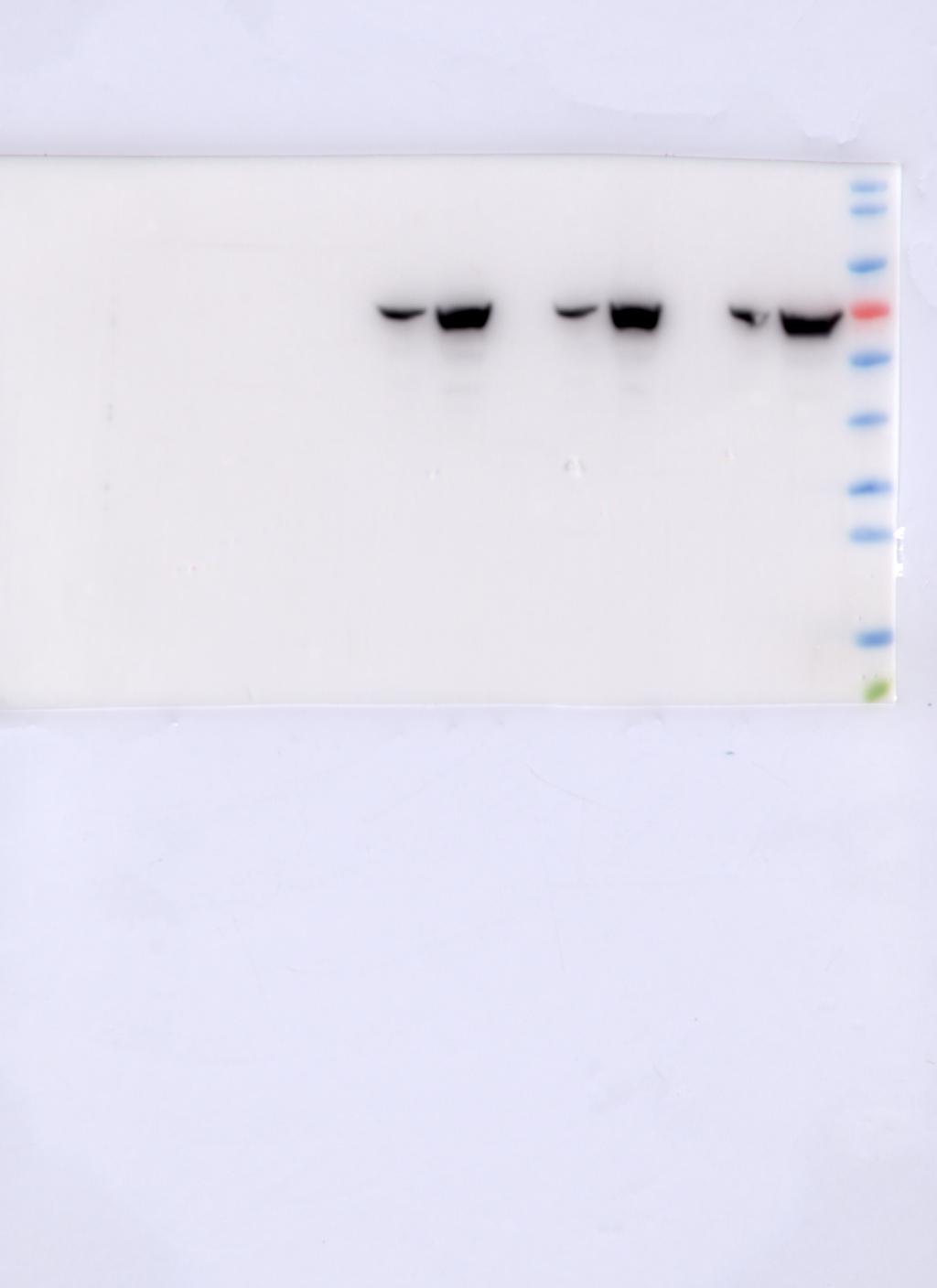

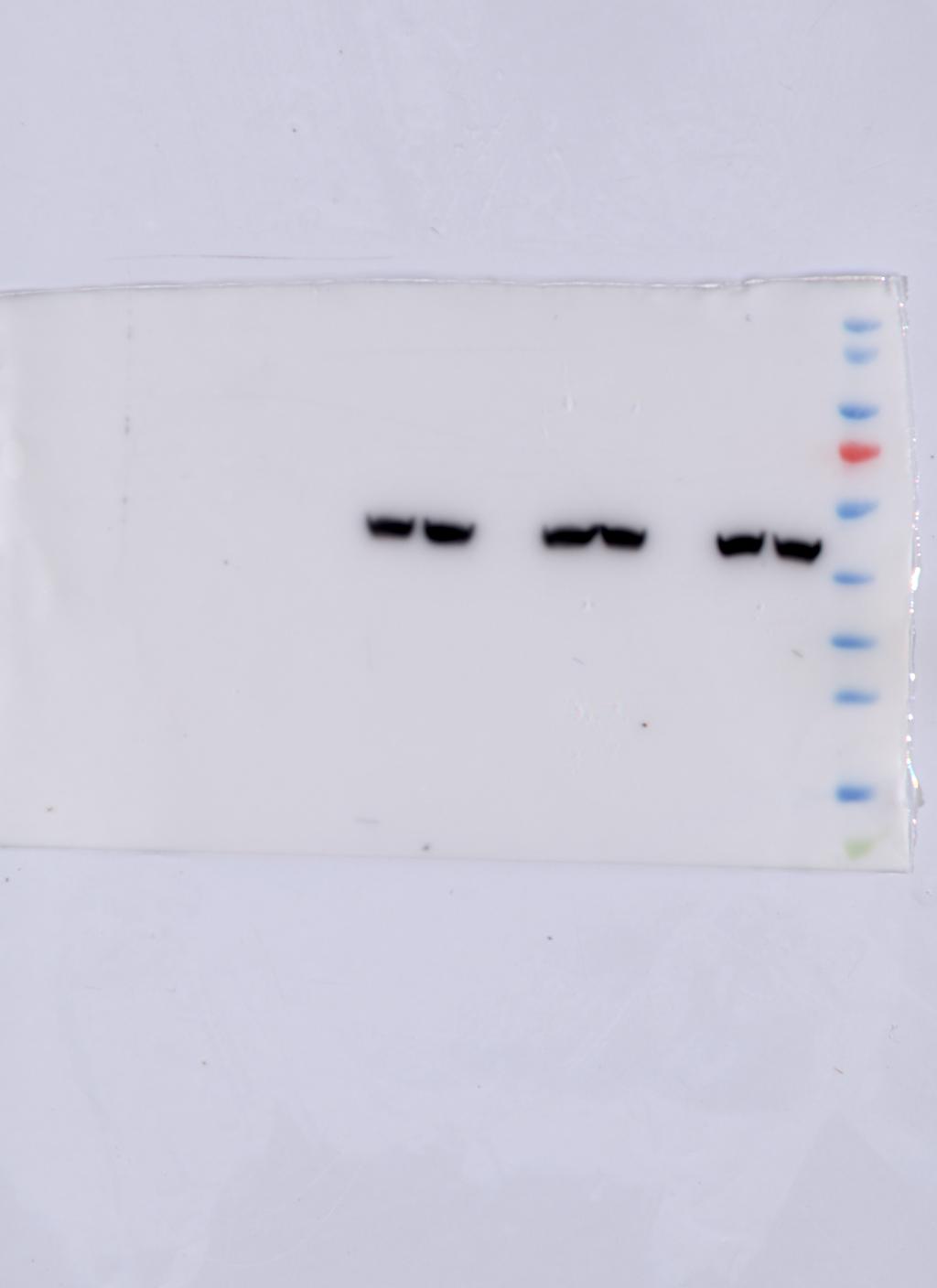


**Fig. S1B**

METTL14 GAPDH


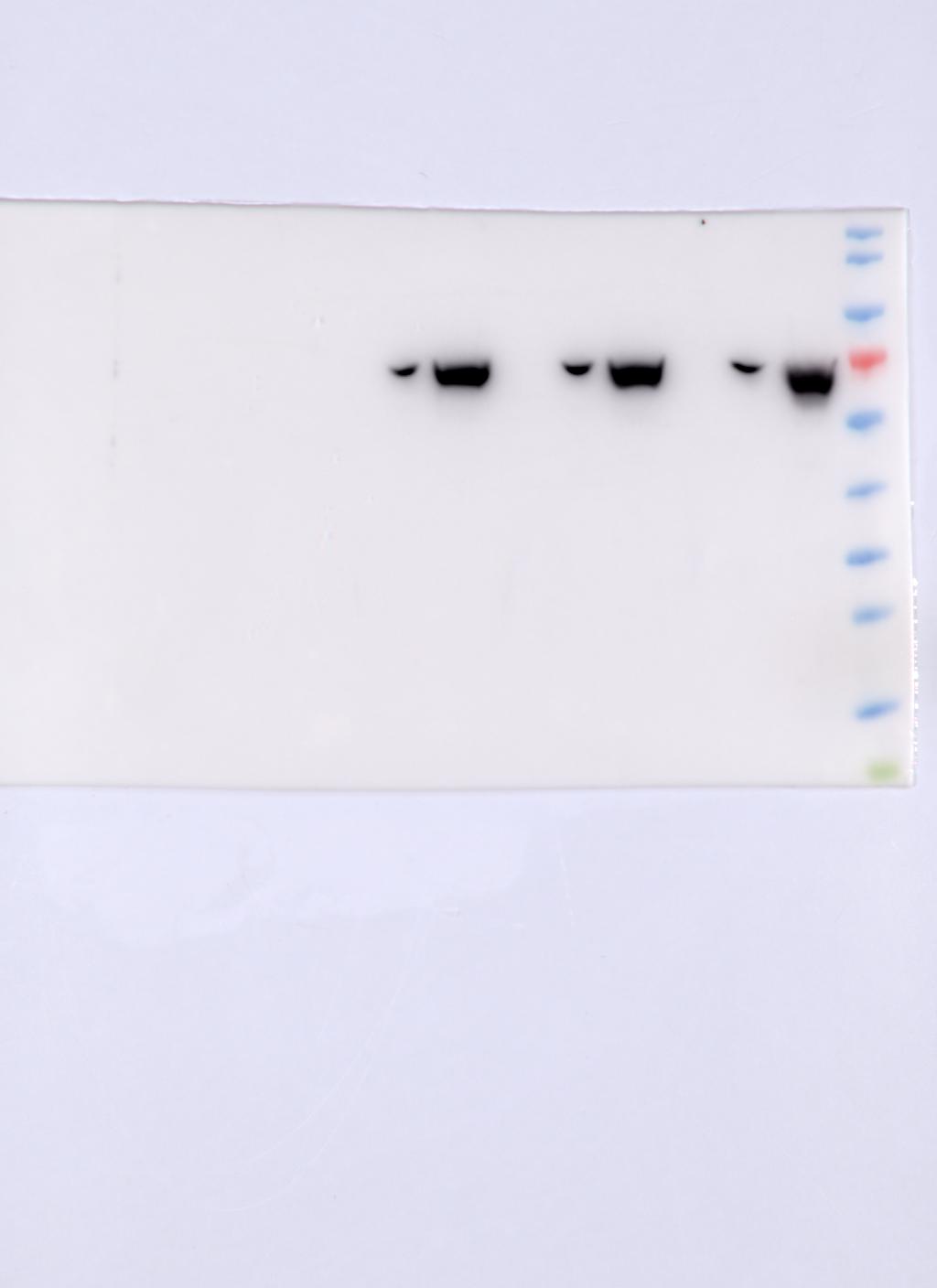

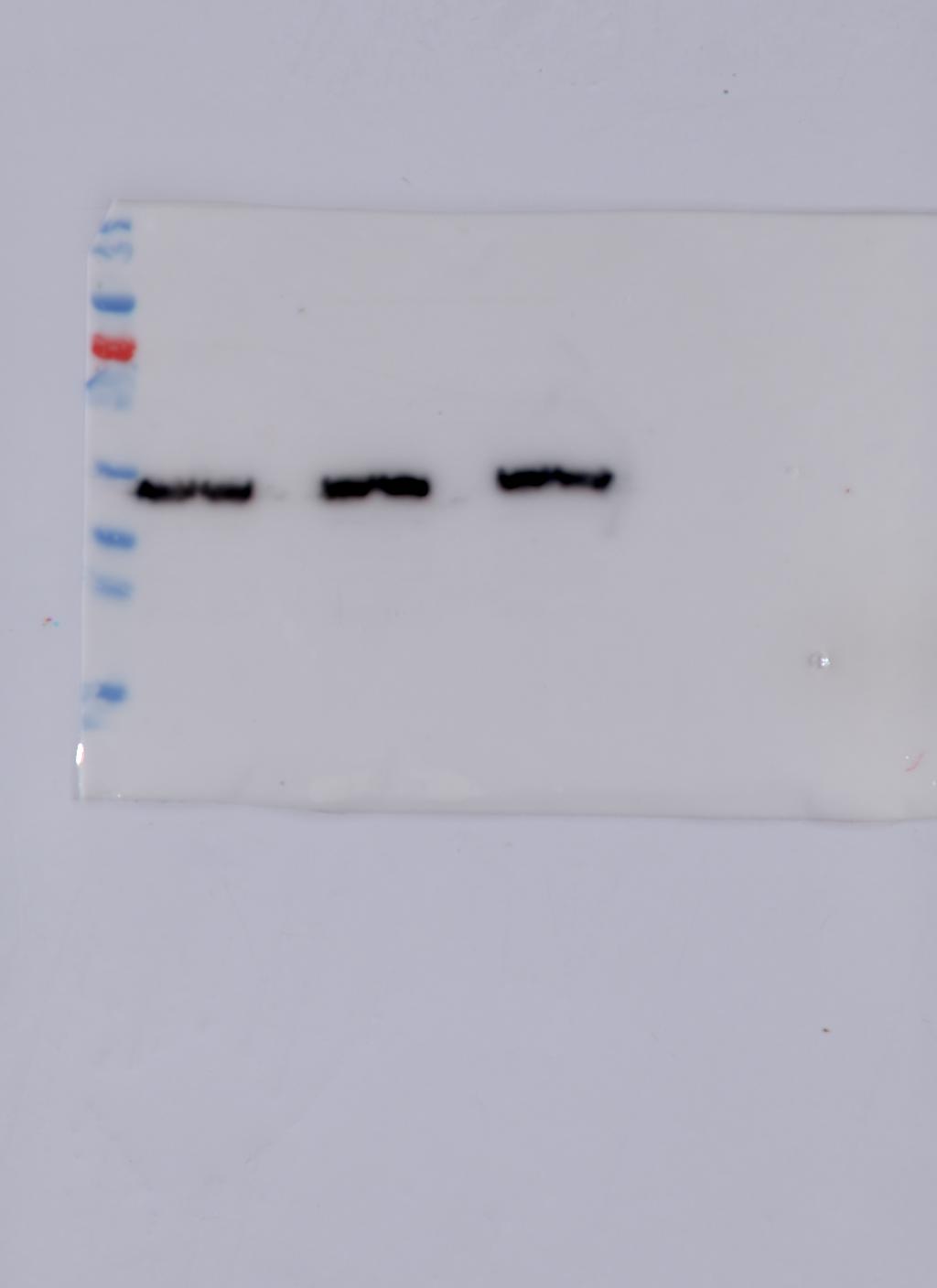


**Fig. S1C**

METTL14 GAPDH


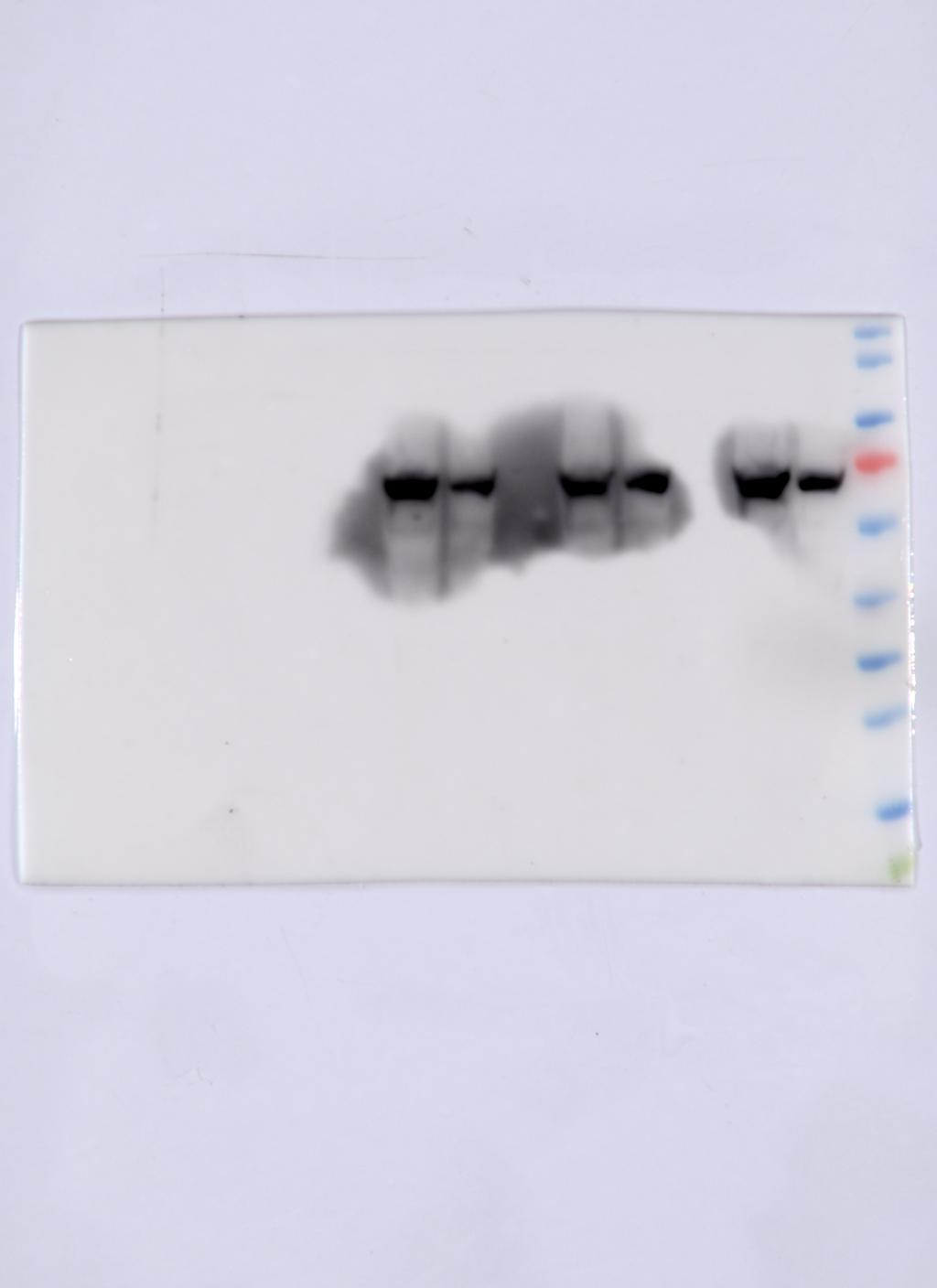

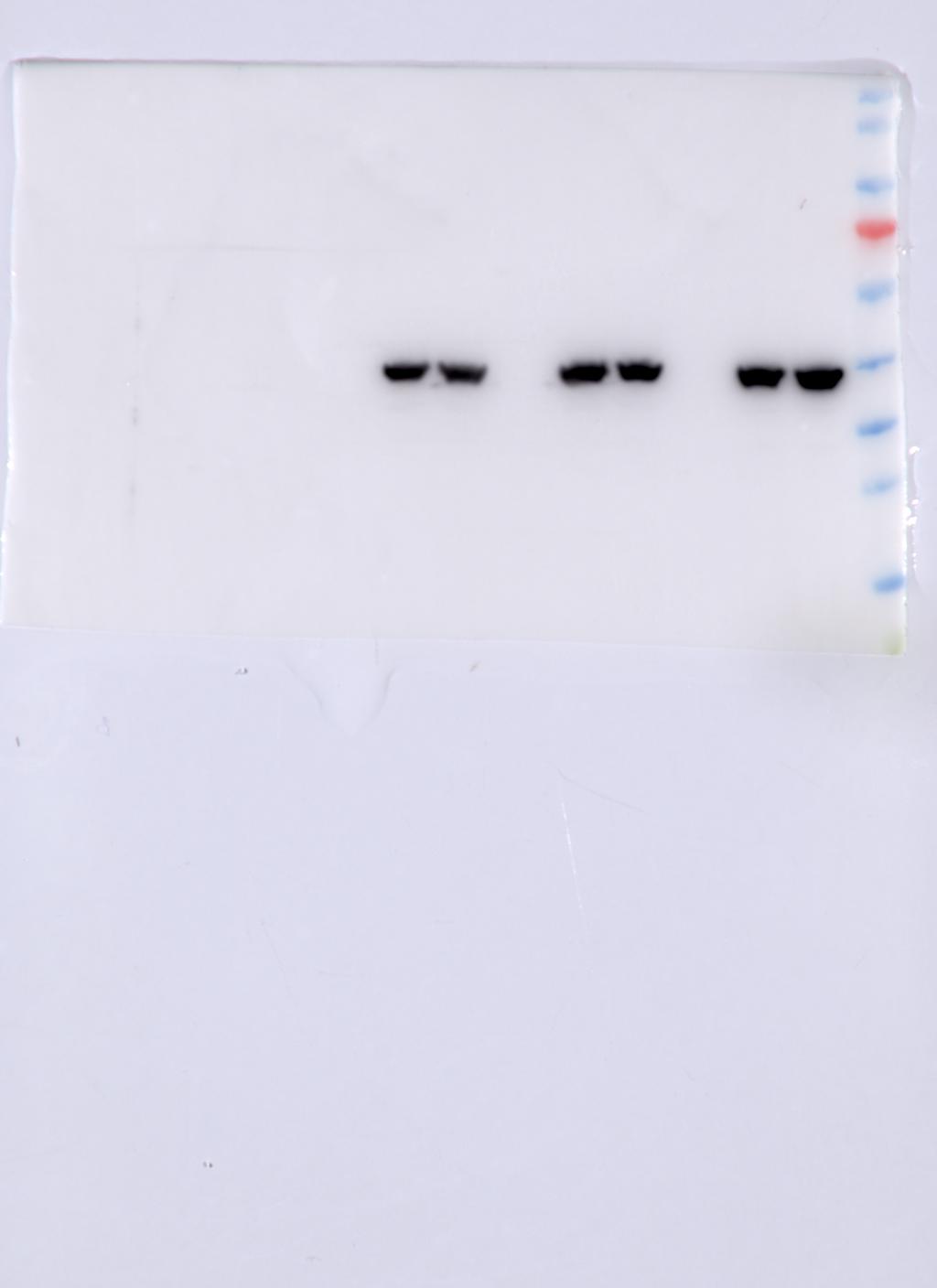


**Fig. S3A**

NFATc1 β-actin


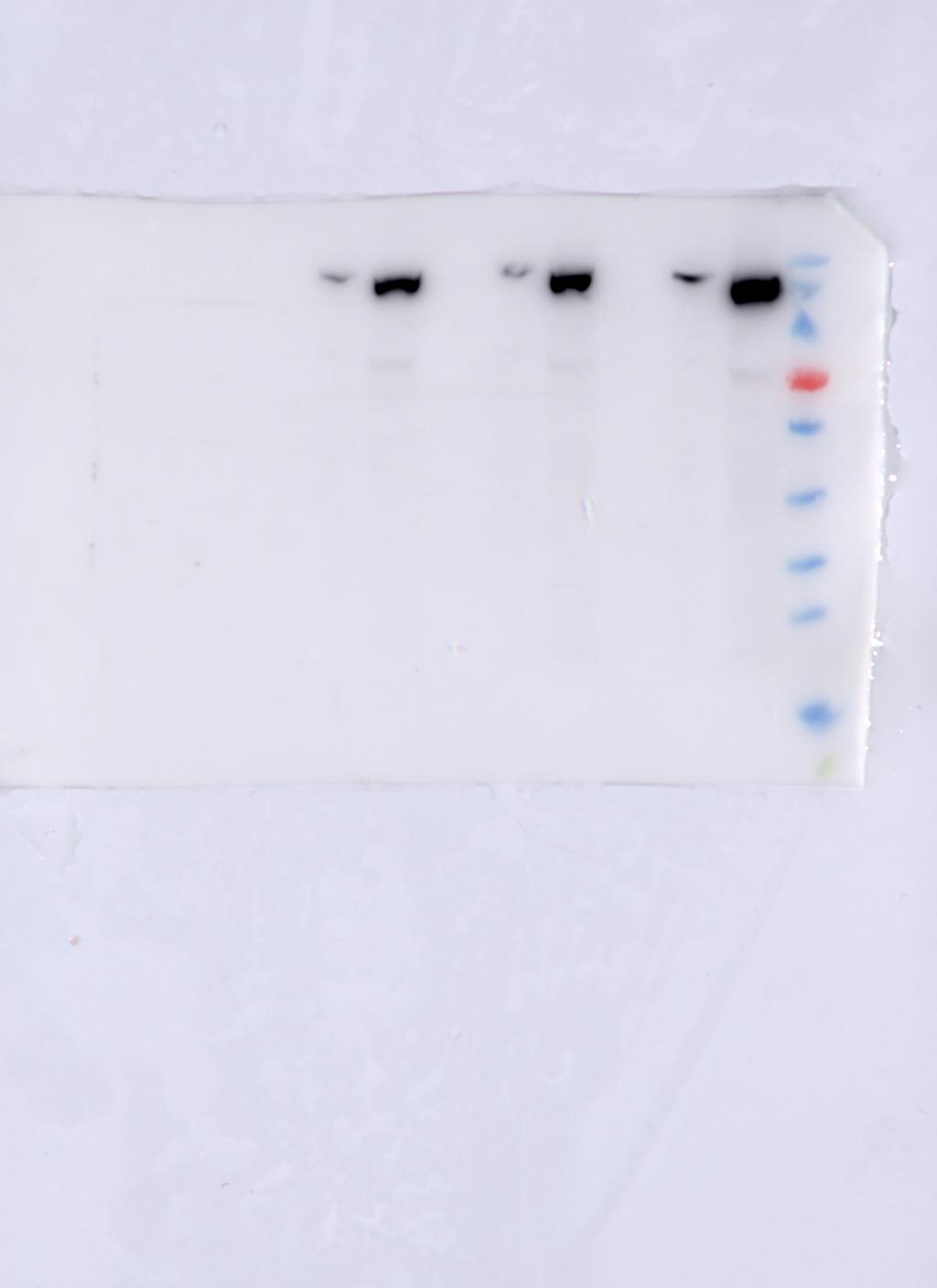

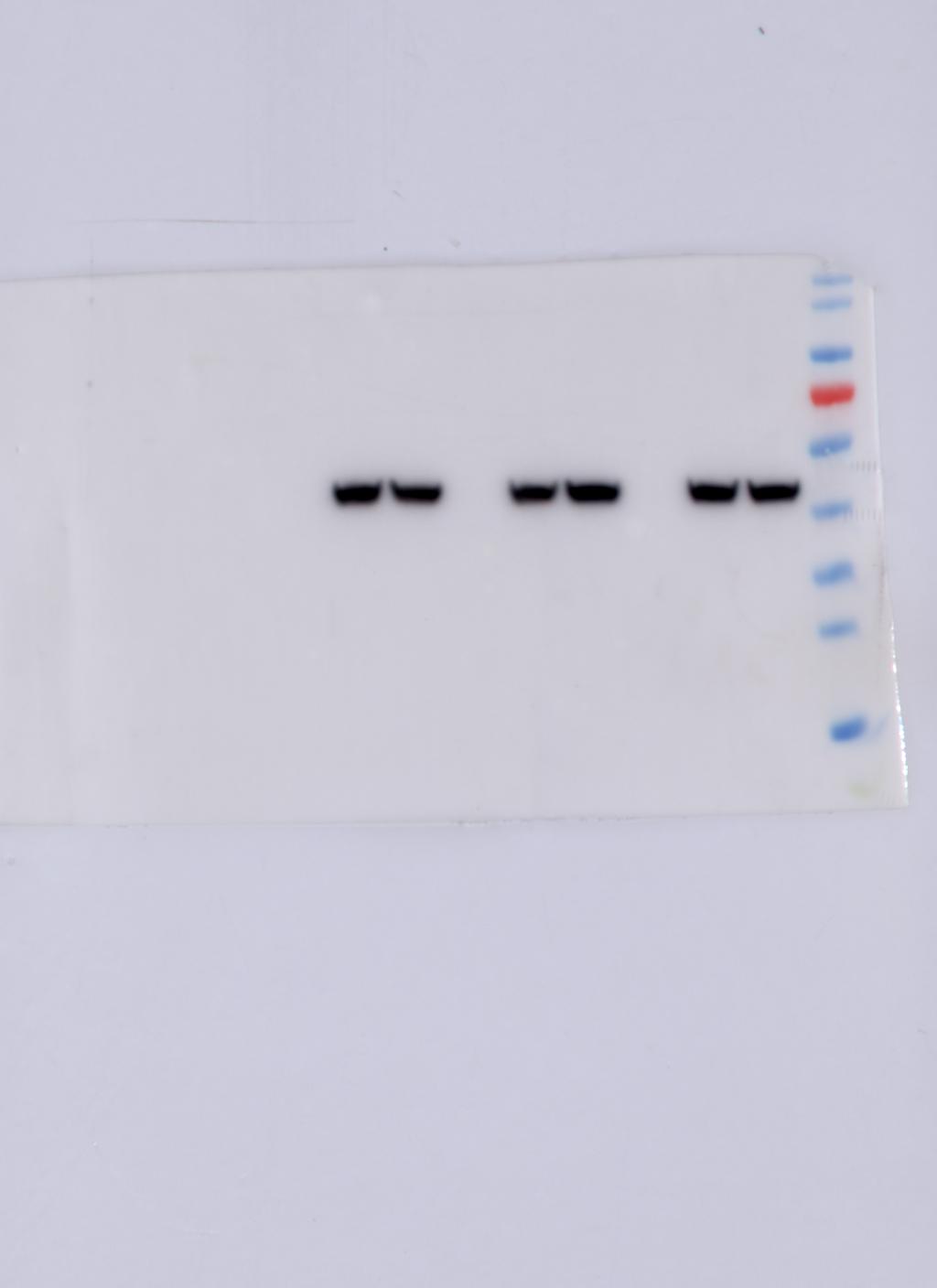


**Fig. S3B**

NFATc1 β-actin


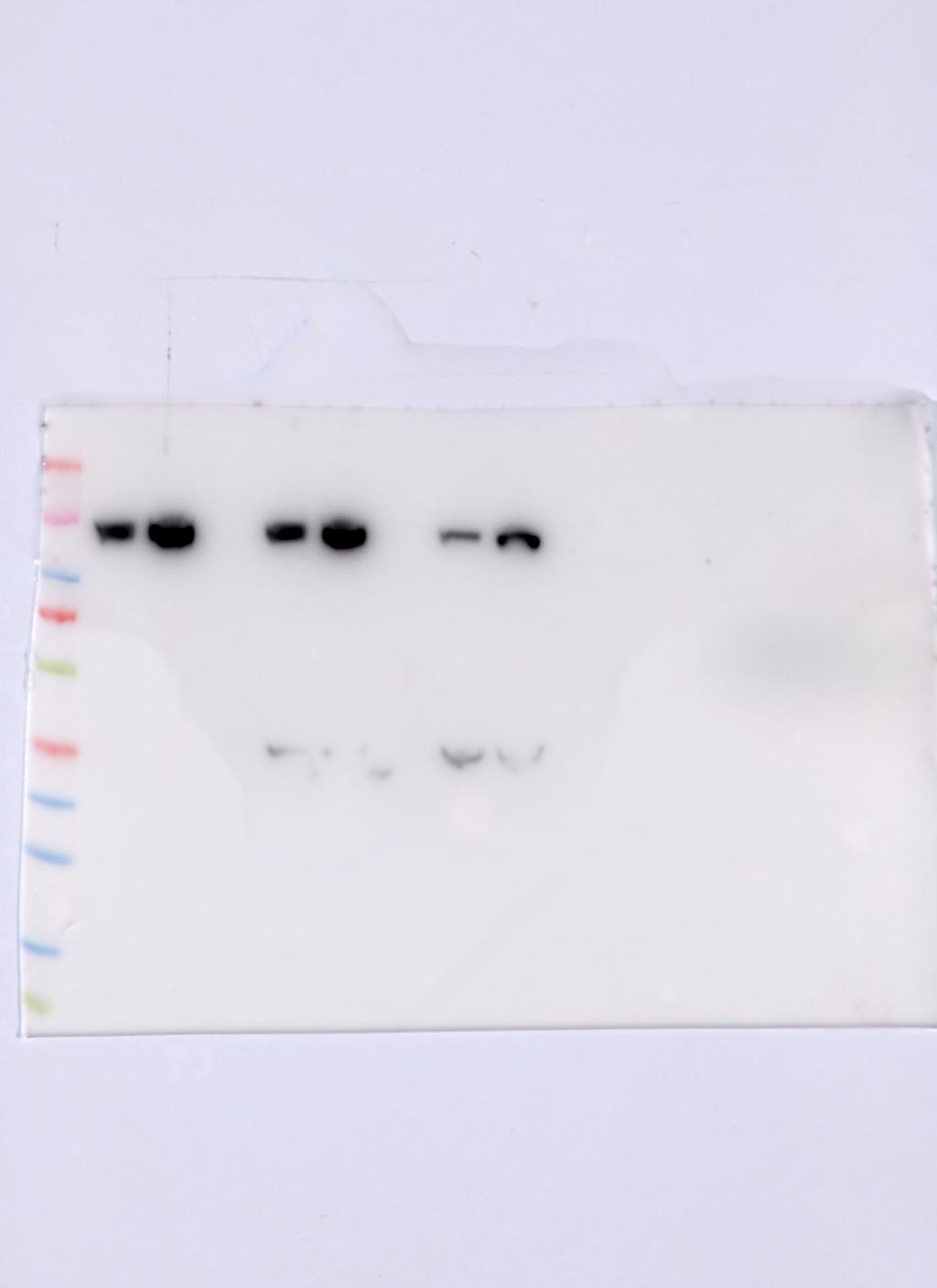

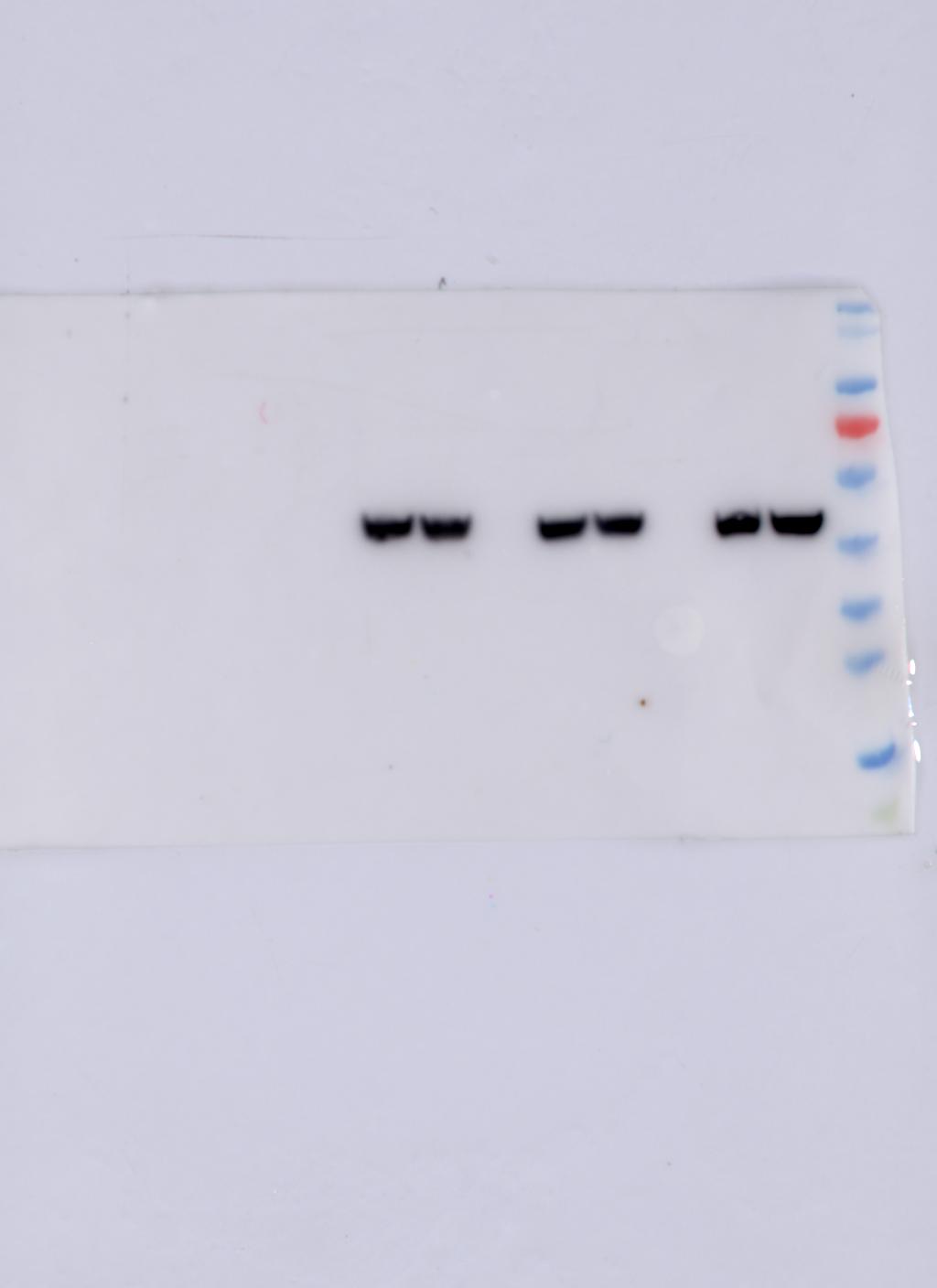


**Fig. S4A**

YTHDF2 GAPDH


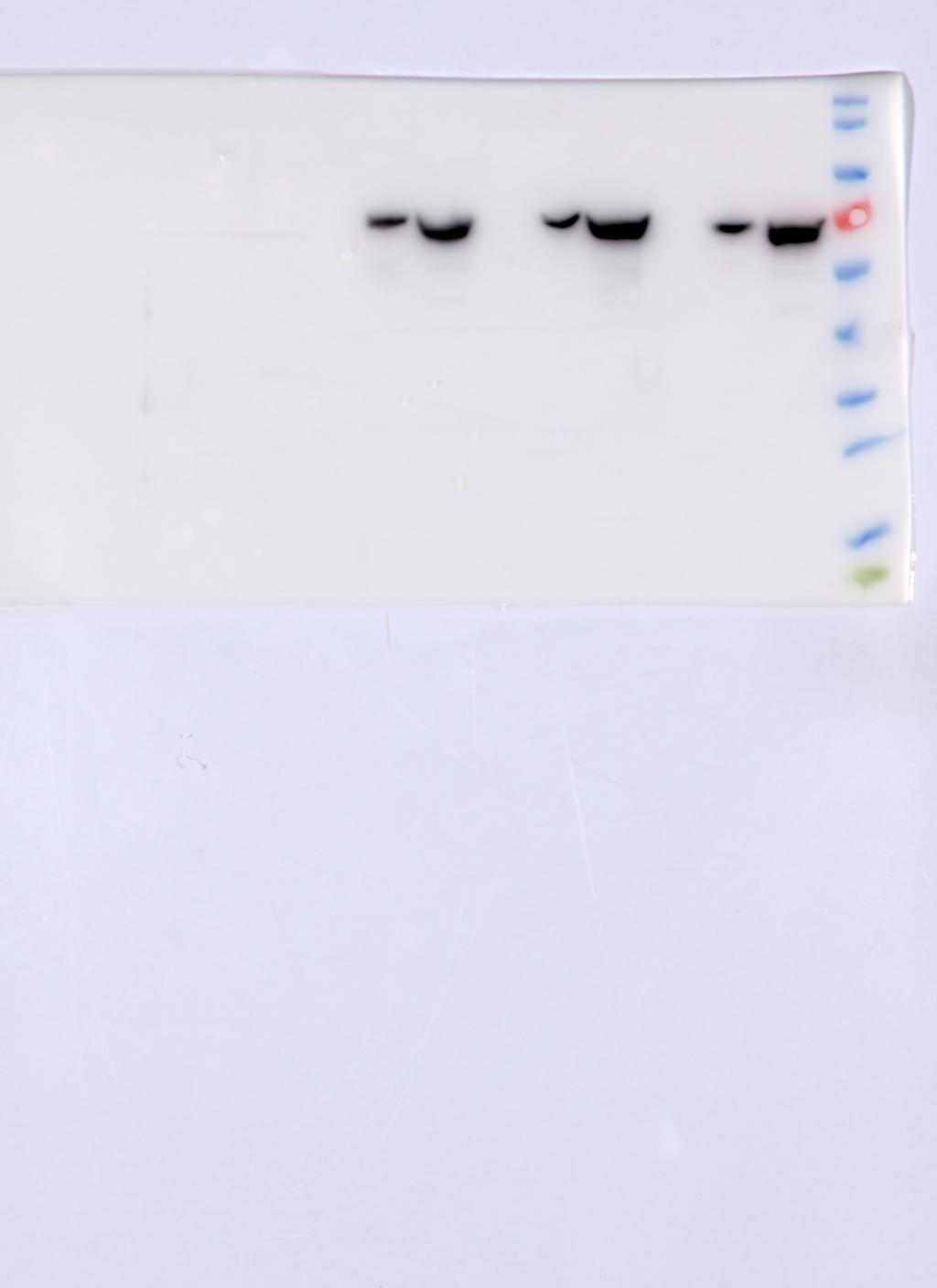

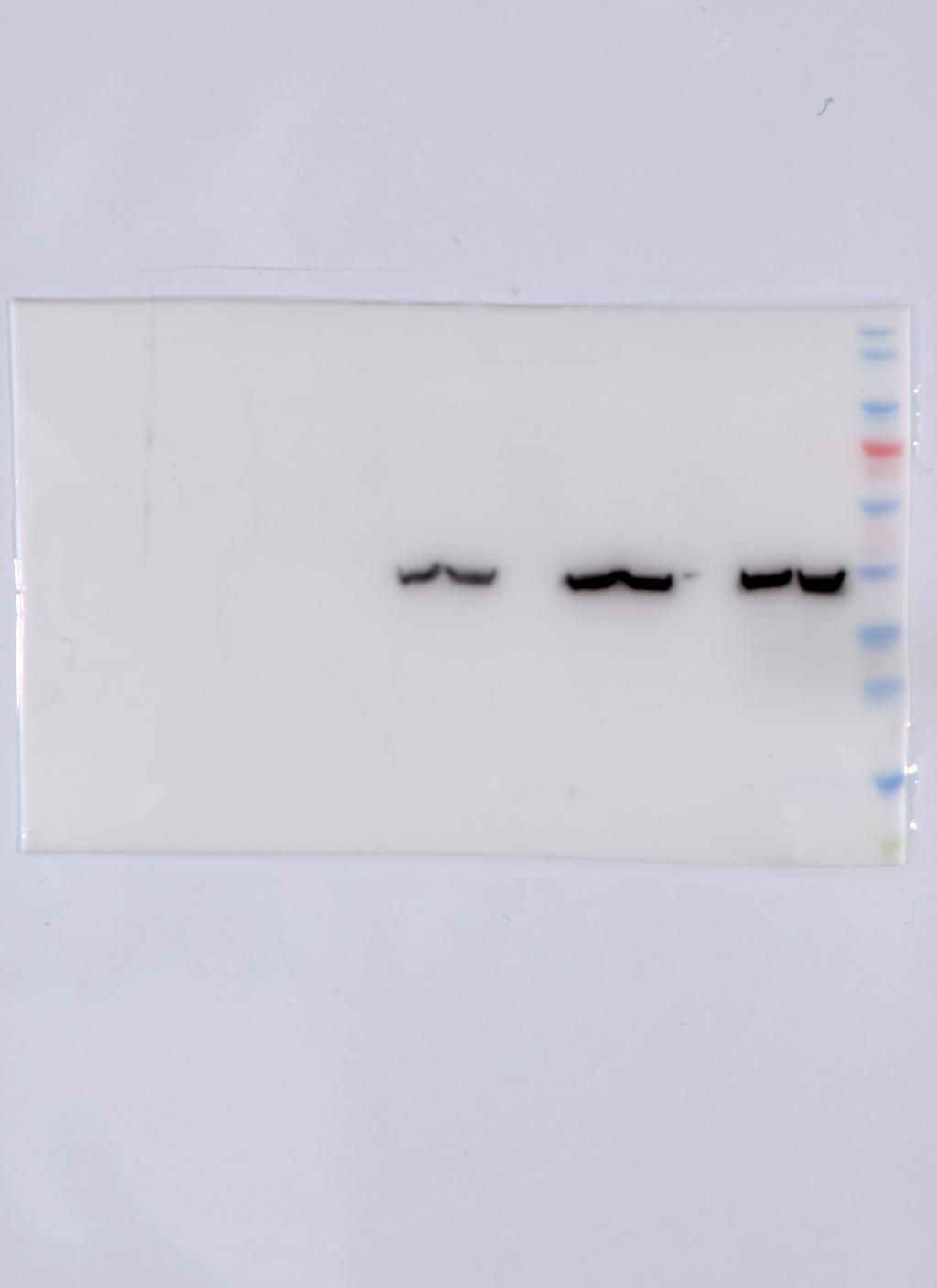


**Fig. S4B**

YTHDF2 GAPDH


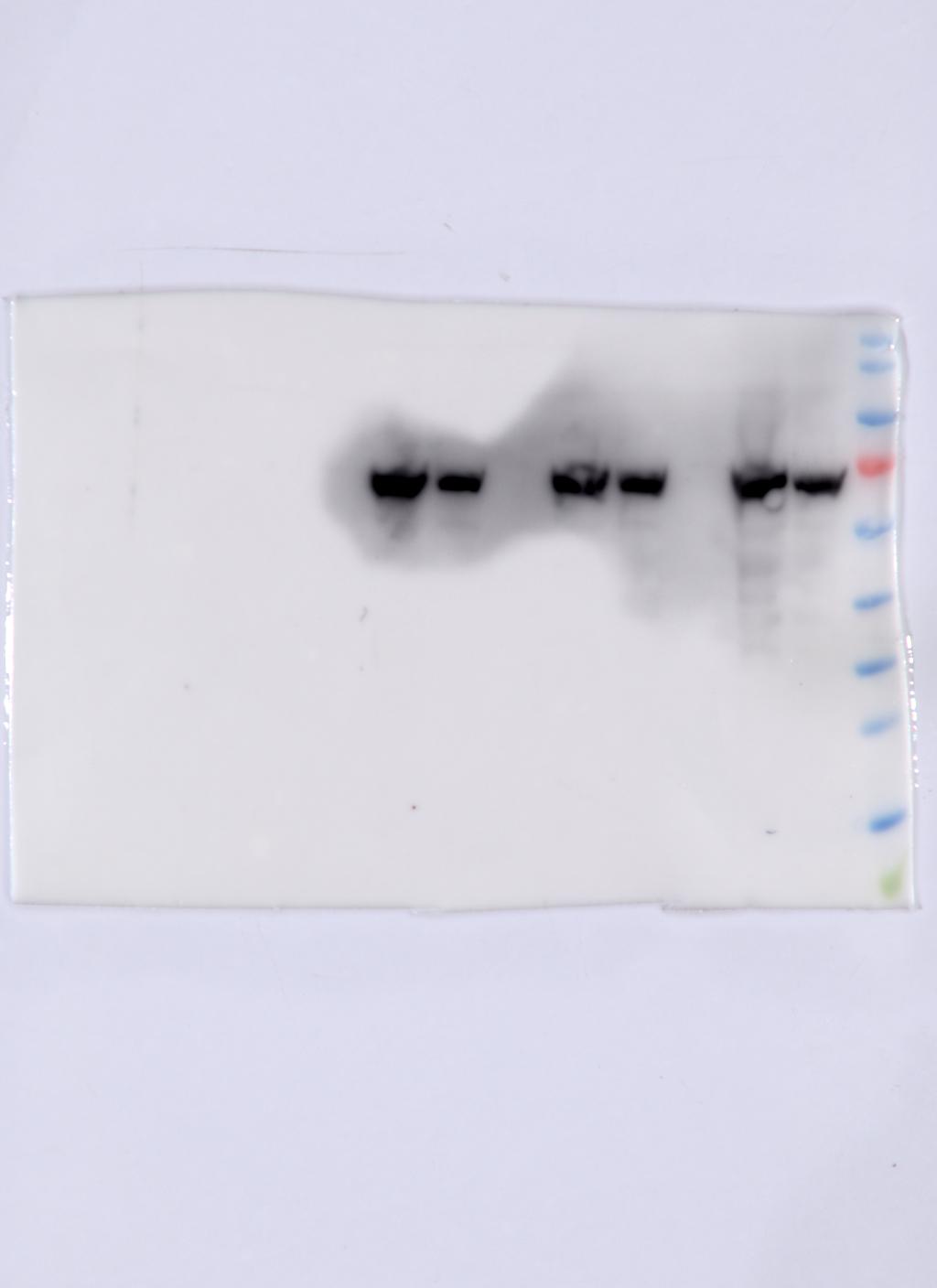

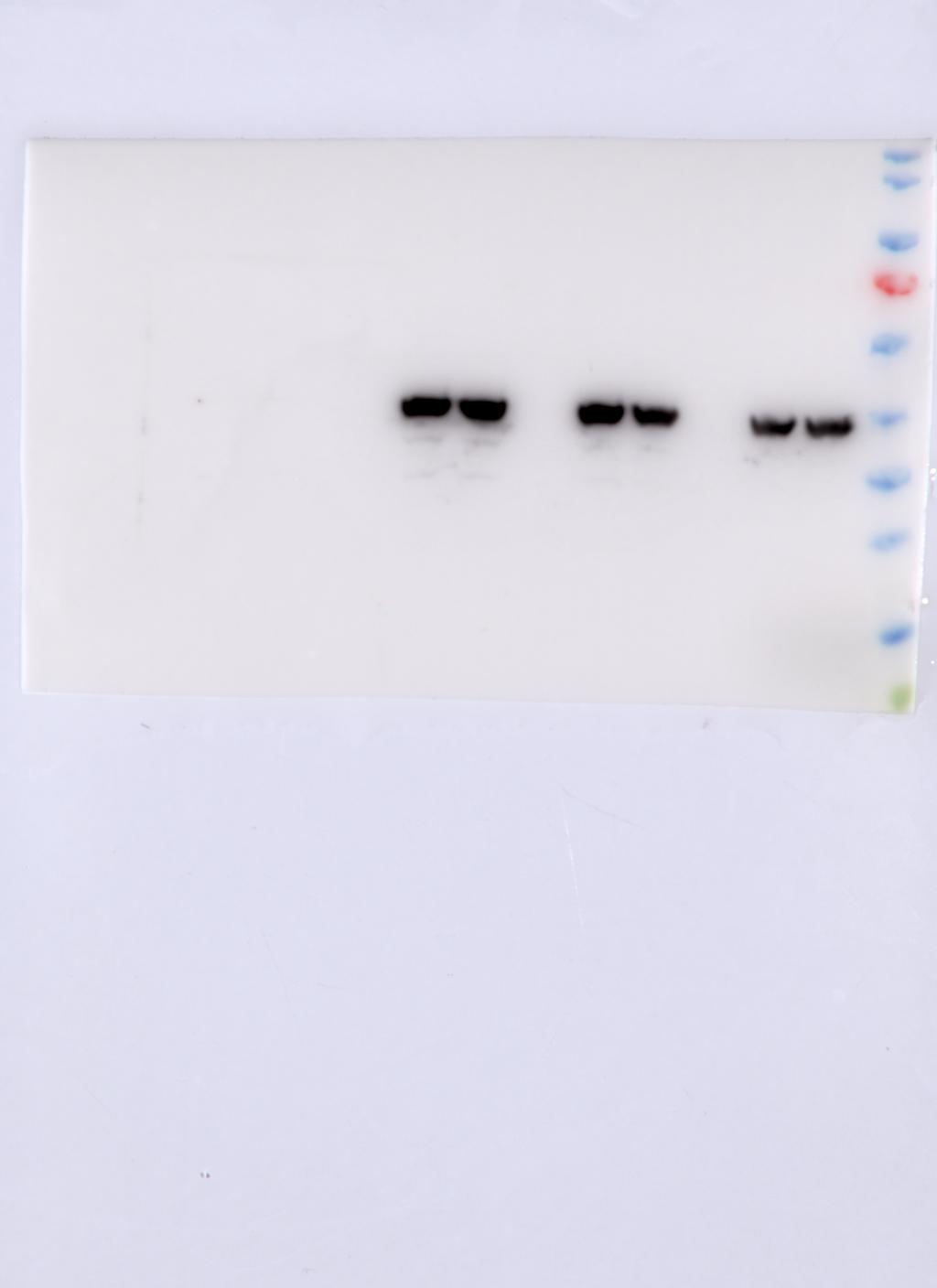


**Fig. S4D**

YTHDF1 GAPDH


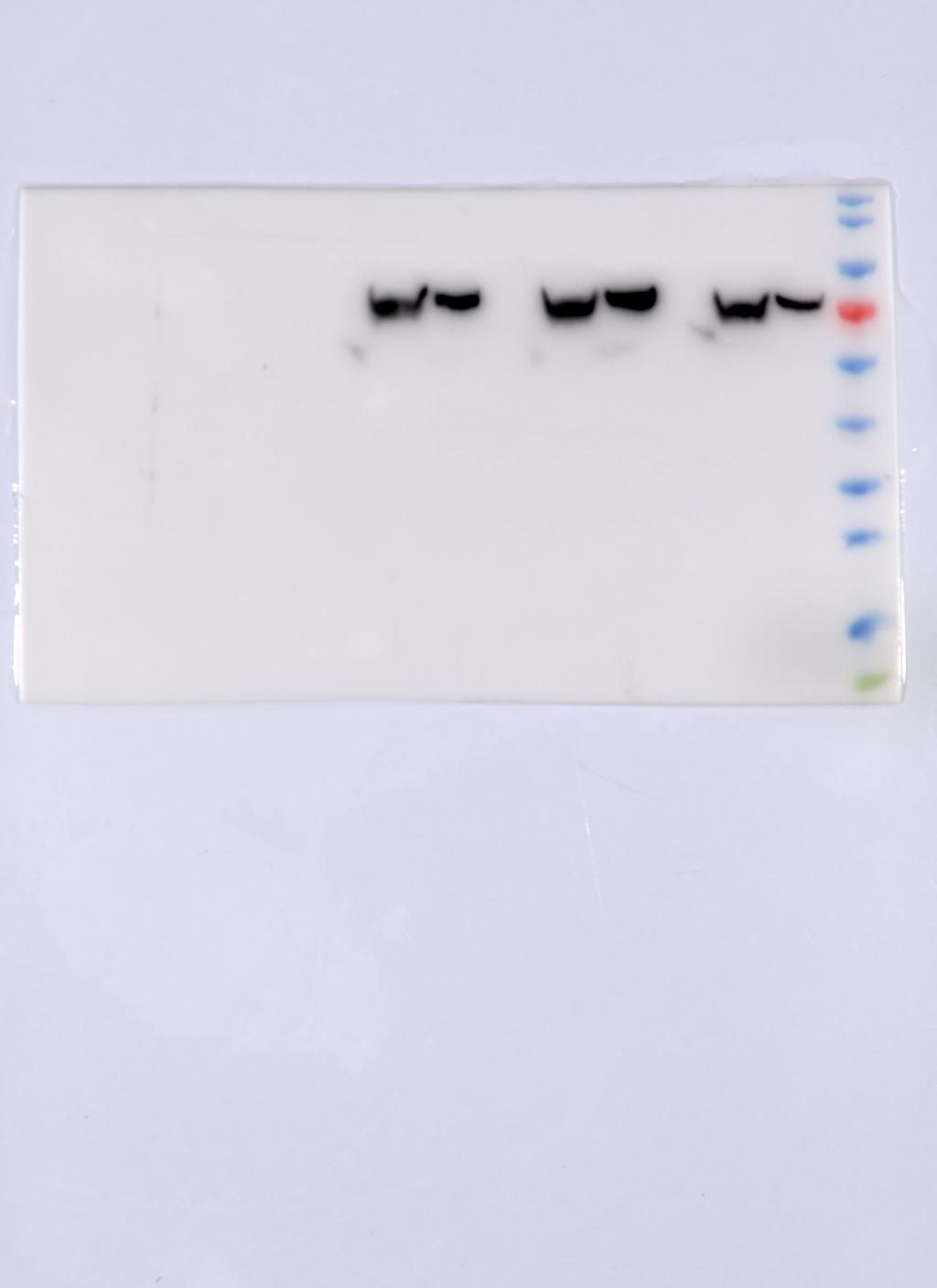

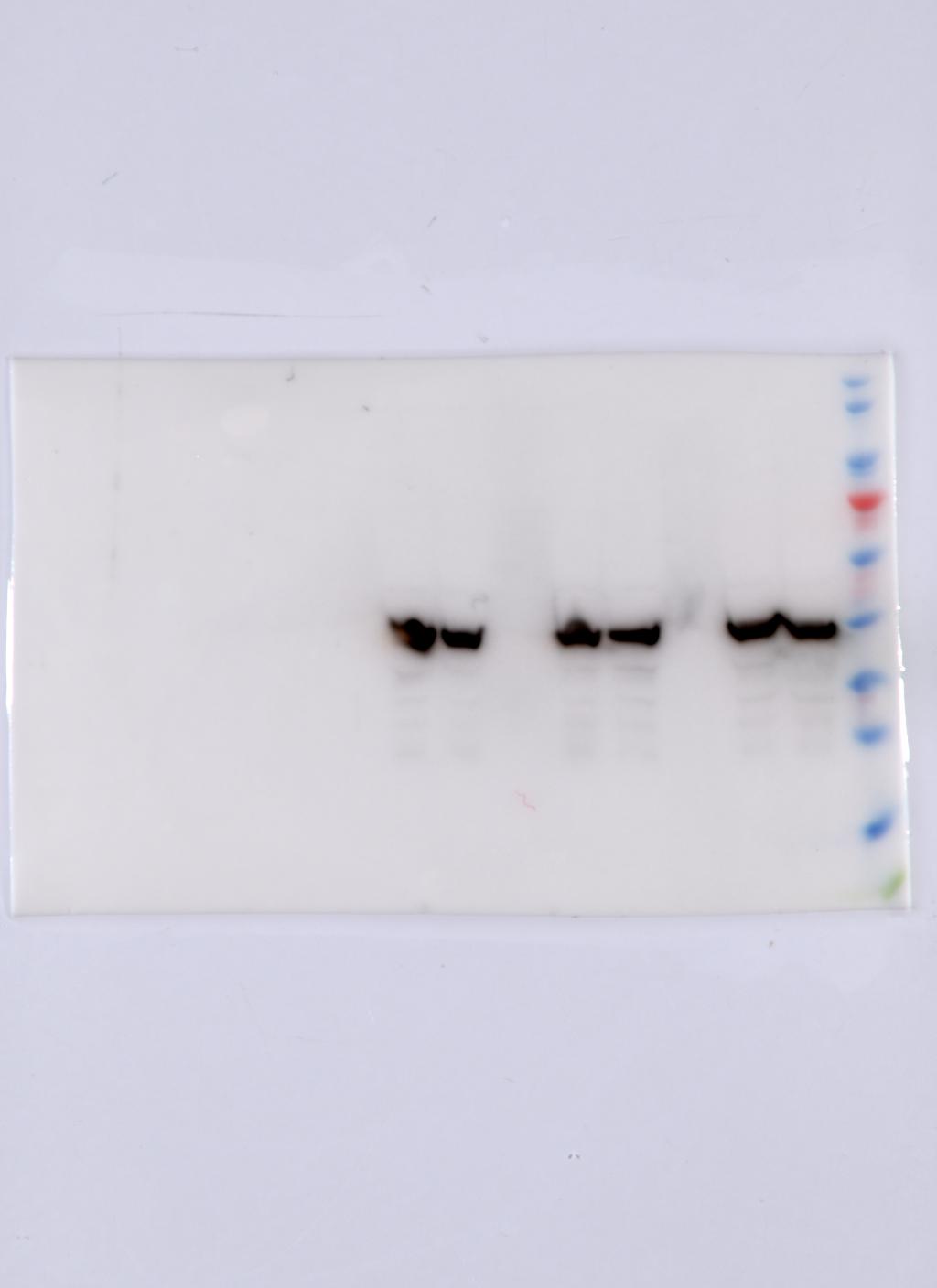

Supplement: Supplementary file 11 — Original Data File [file 41419_2023_6263_MOESM11_ESM.docx]
